# Low-carbon innovation induced by emissions trading in China

**DOI:** 10.1038/s41467-019-12213-6

**Published:** 2019-09-09

**Authors:** Junming Zhu, Yichun Fan, Xinghua Deng, Lan Xue

**Affiliations:** 1 School of Public Policy and Management, Tsinghua University, 100084 Beijing, China; 2 Department of Urban Studies and Planning, Massachusetts Institute of Technology, Cambridge, MA 02139 USA; 3 School of International Business, Southwestern University of Finance and Economics, 611130 Chengdu, Sichuan China

**Keywords:** Climate-change policy, Environmental economics

## Abstract

Emissions trading scheme (ETS) has been adopted by an increasing number of countries and regions for carbon mitigation, but its actual effect depends on specific program design and institutional context. Before launching the world largest ETS, China experimented with seven independent regional pilots, whose effects are only indirectly explored. Here we provide firm-level evidence of the innovation effect directly from China’s pilot emissions trading, based on latest patenting information and a quasi-experimental design. China’s pilots increase low-carbon innovation of ETS firms by 5–10% without crowding out their other technology innovation. The increase from ETS firms accounts for about 1% increase of the regional low-carbon patents, while a similar increase from large non-ETS firms is also induced by the ETS. Most importantly, the effect is not associated with permit price, auction, or firm characteristics, but is driven by mass-based allowance allocation. A rate-based approach, however, is adopted by China’s national market.

## Abstract (web summary)

It is unclear what opportunities for policy evaluation can be created by various independent Emission Trading Scheme (ETS). Here the authors presented the firm-level evidence of policy effects directly from emissions trading and differential program designs in China and find that China’s pilots largely induced low-carbon innovation of ETS firms without crowding out other technology innovation.

## Introduction

Emissions trading scheme has been an increasingly popular policy for climate mitigation: ETS programs cover almost 15% of global carbon emissions at present; 20 programs are in operation, in the European Union (EU), New Zealand, China, South Korea, Kazakhstan, Switzerland, and 22 subnational regions, including California and other states in the Regional Greenhouse Gas Initiative in the United States; 15 more programs have been planned or under consideration^1^. The popularity of ETS may also help to link national and regional climate policies for more efficient mitigation globally^2^. The idea to minimize abatement cost via trading under an emission cap is theoretically appealing, but the actual effect of an ETS requires a closer look. Its performance and relative desirability to alternative policy instruments can be affected by existing policy^3,4^, program coverage^5^, endogenous innovation^6^, and policy specification. Evaluation of the existing ETS programs is therefore important to the advancement in instrument choice and policy design for climate mitigation.

The use of emissions trading in China is of particular interest to researchers and practitioners. China is not only the largest CO_2_ emitter, but also has the largest amount of emissions regulated under ETS. Following its tradition of policy experimentation^7^, China initiated seven regional ETS pilots in two provinces and five cities during 2013–2014^8,9^ before starting the national market in 2017. The seven ETS pilots represent the country’ first explicit use of a market-based instrument for climate mitigation. They have been independently designed and operated, featuring a variety of differences. They are part of the broader low-carbon pilot scheme for climate policy experimentation and potentially interact with other national endeavor for energy efficiency and climate mitigation. The policy design and interaction create rich opportunities for policy evaluation and learning (Supplementary Figs. 1 to 3, Supplementary Table 1, and Supplementary Note 1). Experience from the pilots can be used directly for the development of China’s national market with the world largest emission coverage^10^, and potentially for ETS in other countries.

**Fig. 1:**
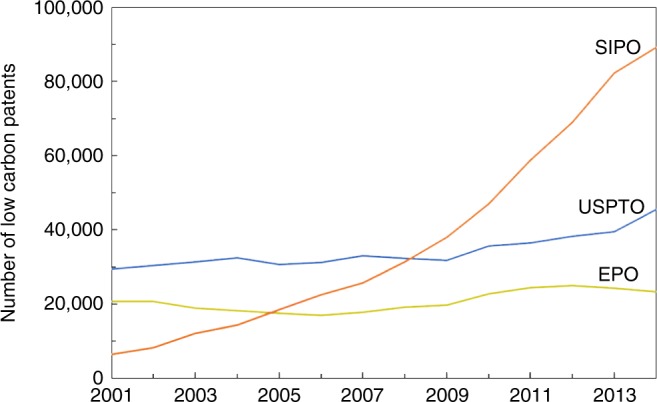
Annual low-carbon patent application at the Chinese, US and EU patent offices. SIPO patents are available via its website (http://www.sipo.gov.cn/zhfwpt/zljs/). USPTO and EPO patents are available via EPO’s PATSTAT database (https://www.epo.org/searching-for-patents/business/patstat.html#tab-1). The scope of low-carbon innovation is the same as used in the main estimation (see Methods section) with reference to the IPC Green Inventory

Here we present firm-level evidence of policy effects directly from emissions trading and differential program designs in China since 2013. We focus on the effect of ETS pilots on low-carbon innovation, based on a quasi-experimental design and disaggregated patent information. Low-carbon innovation helps to break out of path dependency from a carbon-intensive economy^11^ and existing energy infrastructure^12^; it creates a stock of technologies to hedge against future uncertainties in climate mitigation^13,14^, essential for achieving policy targets to stabilize global temperature^15,16^. China has experienced more than ten years of rapid growth in low-carbon innovation. Two years before China’s State Intellectual Property Office (SIPO) became the world largest patent application receiver in 2011, it overtook the US Patent and Trademark Office (USPTO) in receiving more low-carbon patent applications (Fig. 1). Innovation quality has been improving at the same time. As a measure of high-value innovation, triadic low-carbon patents^17^ filed jointly at the SIPO, the USPTO and the European Patent Office (EPO) increased by 14 times, the same as that of total low-carbon patents (Supplementary Fig. 4).

The theoretical literature suggests that climate policies can direct innovative activities toward low-carbon technologies^18,19^, therefore reducing compliance cost and promoting further mitigation in the long term. The empirical literature confirms that increased energy price^20^, carbon tax^21^, and the EU ETS^22^ can facilitate low-carbon innovation. But not much is known about the ETS effects conditional on the presence of other climate policies, the scope of effects among regulated and unregulated firms, and influences from different ETS program design.

Our findings show significant induced innovation effects of the ETS both directly on ETS firms and indirectly on large non-ETS firms on top of other climate policy influences. The identified effects help to sort out previously inconsistent research findings related to but not directly from emissions trading in China: policy announcement in 2011 of ETS pilots has a positive effect on innovation from a small set of publicly listed firms possibly but not necessarily subject to emissions trading^23^; the aggregate effect at the regional level was arguably negative^24^; energy price, as a proxy for carbon price, has a positive effect on clean innovation but a negative one on other innovation^25^. By using the same estimation method, our results are directly comparable to the innovation effect of the EU ETS^22^, showing a similar, significant effect on individual ETS firms but limited impacts at the regional level.

More importantly, our finding reveals the influences from ETS program design. The policy effect was not affected by permit price, auction, or industrial energy intensity, suggesting a weak role of carbon pricing, likely caused by the overall low price and limited trading in several ETS programs. The effect was driven by firms that were subject to mass-based allowance allocation where the number of allowances was pre-established before a compliance cycle; the effect was not significant among firms subject to rate-based allowance allocation, where the number of allowances was updated according to the actual output. Our findings suggest that China’s current national market, with a rate-based approach and inclusion of the power sector only, may deliver additional climate benefits with a broader coverage and a better allocation approach.

## Results

### Rate and direction of ETS-induced innovation

**Fig. 2:**
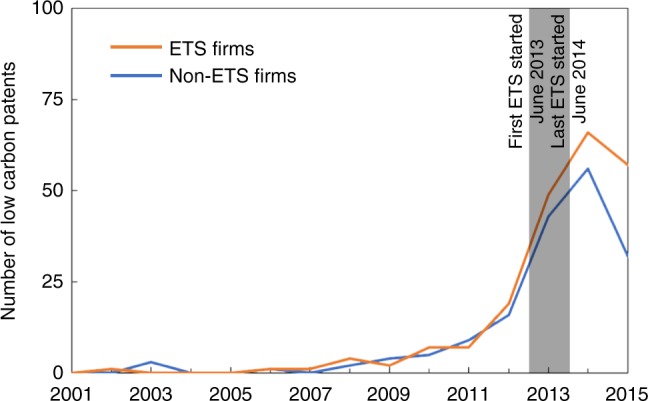
The number of low-carbon patenting by matched ETS and non-ETS firms. ETS and non-ETS firms had similar trends of low-carbon patenting before the ETS started, and diverged afterwards. The gray area marks the period from the first program being started in June 2013 to the last program being started in June 2014

The rate and direction of innovation are essential for achieving climate mitigation targets by affecting the availability, cost, performance, and timing of low-carbon technological change. The main innovation effect of an ETS on low-carbon technologies was estimated in two steps. First, we matched for each ETS firm a similar non-ETS firm as its counterfactual, to control for factors other than the ETS that might have affected innovation (see Methods section). The matched ETS and non-ETS firms shared similar patterns in their historical patent filing (Fig. 2 and Supplementary Fig. 5) and other characteristics (Supplementary Fig. 6, Supplementary Tables 2–4, and Supplementary Note 2). But the two groups diverged after the initiation of seven ETS pilots during 2013–2014, implying a positive effect of the ETS on low-carbon innovation.

**Fig. 3:**
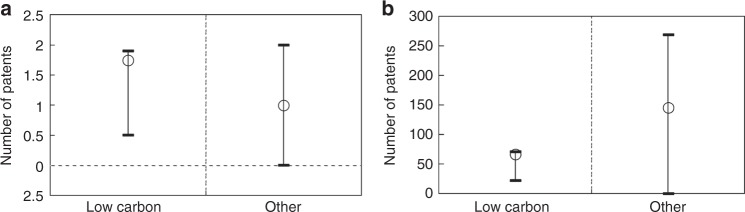
Rate and direction of ETS-induced innovation. **a** Firm-level effects and **b** aggregate effects on the number of low-carbon and other technology patenting and 95% confidence intervals. The aggregate effect was calculated by applying the individual effect to firms with consideration of the corner solution at zero patent

The ETS effect on low-carbon innovation was further estimated by applying a Tobit-modified difference-in-differences (DID) estimator to the matched sample (see Methods section). An average ETS firm filed 1.75 additional low-carbon patents, with a 95% confidence interval of (0.5, 1.9), during the first two years of the ETS (Fig. 3a and Supplementary Table 5). Such an effect was significant and substantial—the EU ETS, in comparison, led to two additional low-carbon patents filed per firm in five years^22^. The effect was consistent across different matching specifications (Supplementary Note 3 and Supplementary Table 6); scopes of low-carbon patents (Supplementary Table 7); more restrictive matching only within ETS regions to eliminate other policy influences (Supplementary Table 8); different baselines and samples to eliminate unobservable selection bias (Supplementary Table 9); and an alternative estimation based on a common parametric DID (Supplementary Table 10). The estimated effect also passed placebo tests, being unlikely a result of chance, any other omitted variable (Supplementary Fig. 7), or regional and firm features (Supplementary Table 11).

Despite the promising policy effect on the matched sample, the overall contribution of emissions trading on low-carbon innovation was limited. To guarantee matching quality, 40% of the ETS firms, mostly being larger and more innovative, could not be matched with non-ETS firms (Supplementary Table 12). If we assume moderately that the same policy effect of 1.75 additional patents applied to these firms, the total effect on all the ETS firms would be 282 (90, 301) additional low-carbon patents considering data censoring at zero, or 10.1% (3.04%, 10.9%) increase (Supplementary Table 13 and Supplementary Note 4). If an alternative matching method based on propensity score is used to have most of the ETS firms matched, the estimated effect would be 0.75 (0, 1.9) additional low-carbon patents individually, 135 (0, 301) additional patents in total, or 4.6% (0, 10.9%) increase (Supplementary Figs. 8–9, Supplementary Table 14, and Supplementary Note 5). Even if we assume no effect on the 40% unmatched firms, an extreme case, the increase from the matched firms would still lead to 2.2% (0.73%, 2.38%) increase of all the ETS firms (Supplementary Table 13). Depending on the assumption, the direct effect of the ETS contributed to around 0.4–2% of the more than 16,000 low-carbon patents filed by firms in ETS regions during 2014–2015. The overall contribution of China’s pilot ETS to regional low-carbon innovation was similar to that of the EU ETS^22^.

A common concern for policy-induced innovation is that increased low-carbon innovation in a firm may crowd out other innovative activities^25,26^. The disproportionate policy impacts would be particularly troublesome if other innovation of high social value were delayed or suspended, creating adverse side-effects of climate mitigation. To examine this, the same estimation method was applied to non-low-carbon technologies. Further subcategorization of patents into detailed technology areas was not pursued, considering the reliability of statistical inference.

Instead of being crowded out, other technology patenting was increased by China’s pilot ETS too. The ETS caused a firm to file one (0, 2) additional patent of other technologies (Fig. 3a), the same as the crowding-in effect of the EU ETS. This was driven largely by increased filing of other green patents: the share of these patents increased from less than 1% to more than 5% among ETS firms, and from less than 1% to just above 1% among non-ETS firms. Either overlapping with or complementary to low-carbon technologies, the availability of these other green technologies supports the previous forecast of co-benefits from China’s carbon pricing^27^. Although 69% of the patents induced by the ETS was non-low-carbon (Fig. 3b), this already represented a shift in innovation direction—the share of low-carbon patents increased to 31% from only a few percent before the ETS.

### Spillovers of policy effects to non-ETS firms

It is possible that the innovation effect of China’s ETS not only pertain to the regulated ETS firms, but also to other unregulated firms. An environmental policy may create a deterrence effect, causing firms that are likely to be regulated in the future to comply in advance. If such policy spillovers existed, the overall innovation effect of the ETS would be greater than a simple aggregation of the firm-level estimation above: the scope of the effect would be beyond ETS firms to unregulated ones; the individual effect on ETS firms might be greater than the estimation above. While the nature of innovation may also create knowledge flows and spillovers, especially to firms in the same technology space as the regulated firms, this process takes time, unlikely significant in the first two years.

We identified three sets of firms that were more likely subject to policy spillovers and estimated impacts on them separately. First, several ETS programs specified their respective lists of non-ETS firms that were required to report CO_2_ emissions annually (Supplementary Table 1). The innovation effect of reporting alone is unclear, considering inconsistent evidence from voluntary programs^28,29^. But these large CO_2_ emitters would have a higher chance to be included in the ETS later because of either their own emission increase or a lower inclusion criteria. Second, we selected in each industry sector of each ETS program the largest non-ETS firms by output. These large non-ETS firms within ETS sectors had a higher chance of being regulated later because of either their own emission increase or a lower inclusion criteria. Third, we focused on the non-ETS sectors not covered by six ETS programs but by the Shenzhen ETS (the most comprehensive in sectoral coverage), and selected the largest firms in those sectors. These firms would likely be regulated if the ETS programs were to expand in sectoral coverage following the Shenzhen ETS. In addition, we tested knowledge spillovers among non-ETS firms that filed patents jointly with ETS firms previously, following the analysis for the EU ETS^22^.

Large non-ETS firms in sectors covered by their regional ETS (second set above) or the Shenzhen ETS (third set above) were significantly induced by the ETS to innovate. We estimated one more patenting from each of the firms in response to the ETS, with 95% confidence intervals of (1, 1) and (1, 1.9), respectively, thanks to their large sample size (Table 1, Supplementary Table 15, and Supplementary Note 6). To confirm that the effect only pertained to these large firms more likely to be included in the ETS rather than sector-wide through knowledge spillovers, we also estimated the effect on small firms in these sectors, showing no significant effect. The spillover effect was not associated with industry competition, patent transfer or license out (Supplementary Note 6), suggesting the unregulated firms’ own demand in response to the policy. With a small sample size, the estimated effect on reporting firms was positive but nonsignificant. Neither could we find any evidence of knowledge spillover yet, as expected.

**Table 1:** Indirect impact of the ETS on non-ETS firms

	Estimate	95% confidence interval	Matched firm pair
Reporting firms	1	(−1, 2.9)	128
Large firms in ETS sectors	1	(1, 1)	1430
Small firms in ETS sectors	0.75	(−0.9, 0.9)	2142
Large firms in Shenzhen sectors	1	(1, 1.9)	1074
Small firms in Shenzhen sectors	0.25	(−0.9, 0.9)	1411
Co-patenters of ETS firms	0.75	(−1, 3.9)	79

Because of the policy spillovers, the innovation impact of the ETS was broader than regulated firms. To avoid underestimation of the direct effect, the ETS firms were matched with firms in the non-ETS low-carbon pilot regions, showing a similar effect of 1.75 (1, 1.9) patents. Because of the nature of spillovers, however, the overall impact cannot be precisely estimated. It was not likely substantial, considering that the ETS firms only contributed to around 1% increase of regional low-carbon patenting and spillovers were limited to large unregulated firms in sectors already or likely covered by the ETS. Further increase of the policy impacts requires a broader program coverage.

### Potential mechanism of the ETS in facilitating innovation

Given significant effects of the ETS on firm-level innovation, it would be important to understand the mechanism through which the ETS facilitated innovative activities. A better understanding of the policy mechanism helps to further improve the effectiveness and efficiency of policy-making for climate mitigation.

The conventional wisdom is that environmental externalities of emissions contribute to underinvestment in innovation and adoption of pollution-control technologies; emissions trading increases the demand for pollution-control innovation by addressing the environmental externalities and creating appropriate price signals for mitigation^30,31^. If this were the case for China’s ETS, one would expect that a higher permit price led to more low-carbon innovation. But a price mechanism could be challenged by the presence of spillovers of policy-induced innovation to non-ETS firms not subject to carbon pricing. The mechanism could also be compromised by the overall low price and limited transaction.

Allowance allocation scheme is also an important feature of an ETS and may affect the degree of innovation^6,32^. In a typical cap-and-trade system, the amount of allowances is pre-established before a compliance cycle, i.e., mass-based. For political, competitiveness, or equity concerns, however, a rate-based system (also known as tradable performance standard) is often used by updating the number of allowances according to the actual output^33^. The former can achieve the social optimum, at least in theory, by setting an emission level to have the marginal abatement cost equal the marginal cost of the externality. Its efficiency also helps to link national and regional programs for global mitigation efficiency^2^. In comparison, rate-based allocation subsidizes output by allowing additional emissions and compromises cost-effectiveness^34,35^, which is exacerbated with heterogeneous benchmarks^36^. The innovation impact from the two methods, however, is likely ambiguous^6,33^. ETS pilots varied in their use of allocation methods, and usually applied different methods to different industries, which may lead to heterogeneous induced-innovation effects.

We evaluated whether the induced-innovation effect was dependent upon pricing, allowance allocation, and related features that varied across programs and industries. Program or firm-specific influences were tested both by a DID estimation with interaction terms between these program features and the treatment status of the ETS and by between-group comparisons based on Wilcoxon’s rank-sum test (Table 2; see Methods section).

**Table 2:** Heterogeneous effects of the ETS across programs and firms

	Dependent variable (∆low-carbon patent)			Wilcoxon’s rank-sum test alternative hypothesis
ETS	−0.007 (0.096)	−0.038 (0.0928)	−0.139 (0.155)	
ETS*average permit price	0.000	0.000	0.003	High price > low price
	(0.002)	(0.002)	(0.003)	*p* = 0.97
ETS*auction = 1	0.018	0.016	0.016	Auction > no auction
	(0.078)	(0.078)	(0.077)	*p* = 0.99
ETS*mass-based = 1	0.069*	0.073**	0.083*	Mass-based > rate-based
	(0.036)	(0.034)	(0.044)	*p* = 0.02
ETS*cap reduction = 1	−0.033	−0.035	−0.030	Cap reduction > no cap
	(0.048)	(0.048)	(0.053)	*p* = 0.07
ETS*energy-intensive = 1	0.015	0.000	0.024	Energy-intensive > other
	(0.050)	(0.047)	(0.052)	*p* = 0.19
ETS*no patent before = 1	−0.011	−0.009	−0.010	No patent before > patent
	(0.041)	(0.041)	(0.046)	*p* = 0.30
ETS*Herfindahl-Hirschman Index	0.588	0.701	0.734	High-competition > other
	(1.406)	(1.377)	(1.425)	*p* = 0.12
ETS*state-owned = 1	0.003	0.072	0.094*	State-owned > other
	(0.035)	(0.043)	(0.046)	*p* = 0.34
ETS*foreign-owned = 1	−0.028	−0.001	0.002	Foreign-owned > other
	(0.036)	(0.037)	(0.028)	*p* = 0.77
Firm characteristics	No	Yes	Yes	
Matched firm pair dummies	Yes	Yes	Yes	
Excluding power plants	No	No	Yes	
Observations	1332	1332	1198	
Adjusted R-squared	0.204	0.208	0.216	

*Note:* Astersisks (*) and (**) indicate 10% and 5% significance levels, respectively. In parentheses are standard errors clustered at the ETS program and province levels for observations in and outside ETS regions, respectively

When considering the interactions from program and firm features, the estimated ETS effect was no longer significant, with a much smaller coefficient than being estimated alone (Supplementary Table 10), suggesting its influences from these features. A higher permit price, however, was not associated with more ETS-induced innovation, shown by a small, nonsignificant coefficient. Neither was auction significantly correlated with induced innovation. Consistent with these was the fact that ETS firms in energy-intensive sectors, who should be more sensitive to carbon pricing, did not have significantly more innovation. All of these suggest a weak role carbon pricing and trading played in the first two years, with overall low price and limited trading in several markets: the highest permit price was less than $20/ton (Supplementary Fig. 2) and the highest average price was lower than $10/ton (Supplementary Table 1); in four out of the seven markets, two-year total trading was less than 5% of the annual allowance (Supplementary Table 1).

**Fig. 4:**
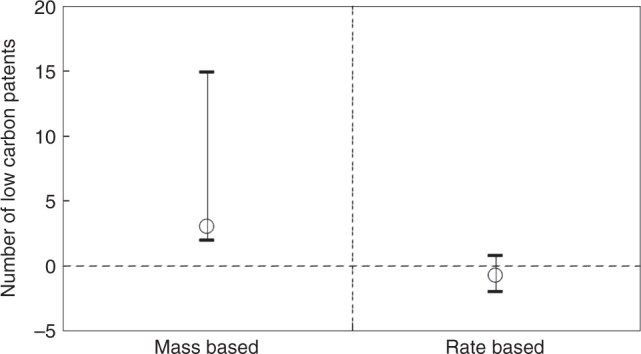
ETS-induced Innovation under alternative allowance allocation approaches. ETS effects on the number of low-carbon patenting and 95% confidence intervals among firms under mass-based allocation and that under rate-based allocation

While pricing and trading did not affect induced innovation, allowance allocation structure did. Firms under mass-based allocation outperformed those under rate-based allocation with significantly more induced low-carbon innovation. Results were consistent in cases both without and with firm characteristics as control variables and in a subsample excluding power plants, which tend to file fewer patents and receive rate-based allowance allocation (Table 2). The mass-based approach actually drove the overall policy effect while the rate-based approach had no effect (Fig. 4).

We further ruled out the influence from other factors, including program commitment in cap reduction, industrial competition^37,38^ (measured by Herfindahl-Hirschman Index), firms’ previous patenting experience^39^, and ownership. Mass-based allocation was the only one that ensured an induced innovation effect from the ETS. It guarantees a binding cap for emissions control, at least similar to a stringent mandatory instrument for policy effectiveness when the market does not operate well for efficiency. Although patent quality has often been a concern, there is no evidence of the quality of ETS-induced innovation being compromised (Supplementary Table 16 and Supplementary Note 7).

## Discussion

As the largest CO_2_ emitter, China’s mitigation efforts contribute to the global climate agenda through direct emission reduction and indirect policy learning and technology diffusion. There has been a patenting surge in China for innovation in general, thanks to a series of reforms^40^ and policy incentives^39,41^. We show in this context emissions trading schemes stood out from other climate policy experimentations to induce firm-level innovation, particularly for low-carbon technologies. The policy-induced innovation effect extended beyond the regulated firms to large unregulated ones in sectors already or likely covered by the ETS. The overall contribution of the ETS to regional low-carbon innovation, however, was still limited, suggesting the need for broader program coverage to increase policy impacts.

The significant policy effect was not affected by carbon pricing yet, with low permit price, limited auction, and limited market fluidity in several programs. Rather, the policy effect was driven by firms under mass-based allocation with the number of allowances established in advance of a compliance cycle. China’s national carbon market has adopted a rate-based approach with the number of allowances being updated by firms’ actual output^42^, which had no effect on inducing innovation in the ETS pilots. A rate-based approach may have its advantage in adaptation and distributional considerations. But our results provide one more reason in favor of a mass-based approach for future development of the market, besides its efficiency advantage and ease of linkage across programs.

## Methods

### Data

Our main dataset of patent information has been made available by the State Intellectual Property Office (SIPO) for free and its associated publisher the Intellectual Property Publishing House Co. Ltd for purchase. It includes information of all the patent applications the SIPO received and published since the first Chinese patent law being enforced in 1985, from both domestic and foreign individuals, firms, and institutions. Information about each patent application includes application number, date, patent title, applicant name and location, inventor, agent, International Patent Classification (IPC) code, and patent type (invention, utility model, or design).

There is a time lag between patent application and SIPO publication of patent information, which influences the timing for collecting patent data in evaluating recent policies. The time lag is usually considered around 18 months^41^, but our investigation shows that to compile more representative patent data, a longer lag would be preferred. Supplementary Fig. 10 illustrates monthly cumulative distribution of published patents by the year of application. After 18 months, around 80% out of the total applications as announced by the SIPO for a given year have been published, and the rate of publication are still increasing rapidly. The share increases to around 90% after 26 months, which is well beyond the turning point and at the flat part of the curves with only trivial increases in following years. As the curves predict, a few percent out of SIPO announced total applications will never be published in a reasonable time frame.

The cumulative distributions of publication months in Supplementary Fig. 10 are for annual cohorts where annual total application numbers are based on SIPO’s announcement. The publication lags for patenting in different months of a year—January and December in the extreme cases—are mixed. Alternatively, we took all patent entries during 2007–2016 in the SIPO dataset as the total population and drew the cumulative distribution of the time lag between application and publication for each patent in Supplementary Fig. 11. It provides a more accurate probability distribution of time lags between patenting and publication in recent years. Again, it shows that only 80% of all patents would be published and available after 18 months, although there is a surge in the 18th month because of required publication. After 26 months, more than 97% of patent information ever published by the SIPO would be available. Therefore, all our results were produced based on data acquired in March 2018, 26 months after the end of our study period in 2015.

Data about patent filing, instead of patent granted, have been used in the analysis, following the previous innovation research in Europe and China^22,39,41,43^. Besides the reason that filing measures firms’ innovative efforts, patent examination decisions also take much longer to be available^41^, making it impossible to evaluate policies in recent years based on data of patent granted. Patent filing is also of better quality and more consistent availability than alternative measurements of innovation, such as research and development expenditure^43^. In total, we collected 13,473,005 published patent applications filed to the SIPO from 1985–2016, including 6,993,314 inventions and 6,479,691 utility models. Among them, firms filed 8,268,320 patents, including 4,516,681 inventions and 3,751,639 utility models.

A well-known issue with Chinese patent filing is the insufficiency and unavailability of citation information, which could have been used as an indicator for patent quality^41^, for two reasons: first, the SIPO and patent regulations in China do not require citation of all related patents; second, the SIPO has not made the citation information available. We addressed this issue in two ways. Our descriptive analysis used triadic patents filed jointly at the SIPO, the USPTO and the EPO to measure high-quality patents. These international patent families are proved particularly valuable^17^. But patent families are extremely rare among firms, and take a much longer time to be available than the common time lag between application and publication at the SIPO. Therefore, in econometric analysis where it is infeasible to use triadic patents, we differentiated patent types between high-quality invention patents and low-quality utility models. The third type, designs, is about exterior appearance of a product and therefore not included in our analysis, similar to the practice in other research^39^.

In order to distinguish a patent of low-carbon innovation from other patents, we used the IPC code accompanied with each patent. Every patent application includes one or more IPC codes, which were assigned by SIPO’s experts to reflect the patent’s technology areas. To identify the low-carbon IPC codes, we referred to the IPC Green Inventory, which was developed by the World Intellectual Property Office with reference to the United Nations Framework Convention on Climate Change. An alternative categorization of low-carbon technologies, Y02 classification, was not feasible, because the SIPO patent entries do not contain related classification codes.

Low-carbon patents in our main results were based on a relatively broad set of areas related to climate mitigation in the IPC Green Inventory: alternative energy production, transportation, energy conservation, carbon capture and storage, nuclear power generation, reuse of waste materials, and administrative, regulatory, and design aspects related to climate mitigation. This classification led to 1,202,975 low-carbon patents, or 9% of total applications, in which 546,799 were filed by firms in China. An alternative, narrow set of areas were used to represent low-carbon innovation in robustness checks, consisting of low-carbon power generation and energy conservation.

The patent data were merged with the Annual Survey of Industrial Firms (ASIF, also known as the Chinese Industrial Enterprise Database) from the National Bureau of Statistics to form a dataset of firms and patents they filed. Being the most comprehensive firm-level dataset in China besides economic census, the ASIF consists of all the state-owned enterprises and other enterprises that are above scale, meaning with annual sales above 20 million RMB (since 2011 for our study period, before that above scale referred to above 5 million RMB).

A fuzzy matching technique was adopted to improve matching rate between firms and patent applicants sharing the same name, in recognition of personal discretion in reporting information for the ASIF and patent application: we broke names into pieces of information and allowed flexibility in the sequence by which the pieces appear and minor variation of their forms, as long as there was no conflict. For example, two names with almost the same characters except that one with location information appeared at the beginning and the other with location information appeared in the middle could be treated as the same firm. Names with the only difference in the suffix of location information (such as Beijing City versus Beijing) would also be considered referring to the same firm. Without causing any conflict, this procedure improved the overall quality in dataset merge. To reflect firms’ annual innovation activities, patents were counted according to the date of application. The merging procedure led to a dataset of 309,656 firms, who filed 2,000,120 patents, including 147,102 low-carbon patents. The regulatory status of a firm with regard to the ETS has been generally available through lists of ETS firms publicized by the local governments. In rare occasions where a list was unavailable, we filed information disclosure requests to obtain firm lists.

### Empirical strategies

The identification strategy for the effect of the ETS on innovation has been matching-adjusted DID that proceeded in two steps^44,45^, used for estimation of both the main policy effects and spillovers. The same strategy has been used, for example, to evaluate the effect of NO_X_ emissions trading in Southern California^46^ and European Union ETS^22^. The first step selected and matched ETS firms with similar non-ETS firms, conditional on their observable characteristics, to remove potential biases in sample selection caused by policy design or other confounding factors; the second step estimated the difference-in-differences of the matched ETS and no-ETS firms to account for firm-level heterogeneity and time trends, with adjustment for the issue of a corner solution at zero patent.

Matching was the preferred strategy to make the regulatory status under the ETS appear to be a random assignment, conditional on observable characteristics of firms. It took advantage of the fact that seven independent ETS programs all adopted different firm inclusion criteria, with regard to industrial sector (4 to 26 sectors), emission threshold (3 to 150 thousand tons of CO_2_), and the base year to measure emission for inclusion (2009–11, 2010–2011, 2009–2012, 2011–2012, or 2008–2012). The differences in inclusion criteria across programs led to an overlap in the joint distributions of pre-ETS covariates of ETS and non-ETS firms.

Two sets of marginal differences between ETS and non-ETS firms were explored for a quasi-experimental design via matching: two firms may be identical except their locations, so that one (an ETS firm) is in the ETS, and the other (a non-ETS firm) is in place of no ETS but other climate policies, of an ETS with a narrower sectoral coverage, or of an ETS with a higher emission threshold for firm inclusion; two firms may be identical except their highest emission levels during the base years for inclusion measurement, which does not necessarily indicate a difference in pre-treatment emissions.

Similarity between ETS and non-ETS firms was measured by a nearest neighbor matching estimator^44,47^. It required a firm pair to be exactly matched on the four-digit industrial classification code (the most detailed level), on location within low-carbon pilot regions, and to have the shortest Mahalanobis distance. The Mahalanobis distance of a firm pair was calculated based on their pretreatment innovation and common predicators of innovation, including total asset, employment, age, low-carbon and total patenting in the pretreatment period (2011–2012), and accumulative levels of low-carbon and total patenting by 2012. The remaining covariates were log transformed except for age, and patent count plus one was used in log transformation. In addition, quadratic terms were used for all the patenting related covariates to improve matching quality on innovation. Considering both matching quality (i.e., similarity between a matched firm pair) and representativeness of a matched sample, we set a caliper of 1.5 to remove matched pairs with longer Mahalanobis distances. Replacement was allowed so that a non-ETS firm could be used as a match for multiple ETS firms. Matching quality was evaluated by comparing between the two groups their pretreatment levels of the matching variables, as well as output, which was left out of matching for evaluation only (Supplementary Tables 3–4 and Supplementary Figs. 5–6).

The second step applied a nonparametric Tobit-modified empirical-likelihood-based DID estimator that has been used in estimating the effect of the EU ETS^22^. The advantage of this estimator over a common DID is that it estimates precisely the number of patenting while accounting for the issue of a corner solution at zero—firms with optimal patenting choice at zero may possess different levels of innovation intention. In addition, the nonparametric nature of the estimator does not assume a specific distribution of patent data. It searched for a treatment effect which, when being subtracted, made the distributions of ETS and non-ETS firms’ first differences most similar, where similarity was measured by Wilcoxon’s signed-rank statistic^48^. A Tobit modification was applied to the subtraction process to calculate the DID of each firm pair to account for the corner solution at zero:

1 $$\delta _j = \left\{ {\begin{array}{*{20}{l}} {\max \left( {Y_{j{\mathrm{T}}1} - \tau - Y_{j{\mathrm{T}}0}, - Y_{j{\mathrm{T}}0}} \right)-\left( {Y_{j{\mathrm{C}}1}, - Y_{j{\mathrm{C}}0}} \right)if\,\tau \ge 0} \hfill \\ {\left( {Y_{j{\mathrm{T}}1} - Y_{j{\mathrm{T}}0}} \right) - \max \left( {Y_{j{\mathrm{C}}1} + \tau - Y_{j{\mathrm{C}}0}, - Y_{j{\mathrm{C}}0}} \right)if\,\tau \ < \ 0} \hfill \end{array}} \right.$$

where *Y* is the number of patent filing; firm pairs are indexed by *j*; *τ* is the treatment effect being searched; *δ*_*j*_ is the DID used for calculating Wilcoxon’s statistic; T and C denote ETS and non-ETS firms in a pair; 1 and 0 denote posttreatment (2014–2015) and pretreatment (2011–2012) periods, respectively.

Matching-adjusted DID estimators assume conditional unconfoundedness, i.e., assignment of regulatory status is independent of the potential outcome distribution of firms had they not been assigned to the ETS, conditional on the observables. To assess this assumption (which is not directly testable in principal) and general robustness of our results, we tested a set of alternative strategies and specifications, including different calipers, number of non-ETS firms matched with each ETS firm, location restriction in matching, estimation strategy, scope of low-carbon technologies, samples, baseline periods, as well as placebo tests (Supplementary Tables 6–11 and Supplementary Fig. 7). Balance tests for output, which was unobserved in the matching process, also helped to confirm conditional unconfoundedness (Supplementary Tables 3–4 and Supplementary Figs. 5–6).

The same estimation strategy was used to test policy spillover effects. It matched non-ETS firms potentially subject to spillovers—reporting firms, large firms in ETS sectors or in Shenzhen ETS sectors, co-patenters of ETS firms—with other non-ETS firms not subject to these influences. Unlike clear-cut inclusion criteria, however, spillovers take networked channels and can be ambiguous. Therefore, the same identification strategy may not be as strong as in the main estimation.

Besides our preferred strategy that can estimate a constant treatment effect and account for the corner solution at zero patenting, we also adopted an alternative estimation strategy after the same matching process. This strategy followed common practices in research using patent data, and could be extended to investigate whether ETS program specification and composition of firms and industries led to heterogeneous induced innovation effects. For point estimation of the general effects, a common parametric DID was used:

2 $$\Delta {\rm{Patent}}_{ij} = \alpha _j + \beta _1D_{i = {\mathrm{T}}} + \varepsilon _{ij}$$

where matched firm pairs are indexed by *j* and the treatment status in a firm pair is indicated by *i*; *α*_*j*_ is a set of firm pair dummies; $D_{i = {\mathrm{T}}}$ is a dummy variable with the value one for ETS firms; $\varepsilon _{ij}$ is the error term; $\Delta {\rm{Patent}}_{ij}$ is the first difference of the natural log of patent count plus one, often used to address inflated number of zeros in patent data^49,50^.

The application of the main estimation strategy to heterogeneous treatment effects in investigation of potential ETS mechanisms would be computationally difficult, considering the calculation of empirical likelihood. Instead, we tested heterogeneous treatment effects based on the common DID of Eq. 2:

3 $$\Delta {\rm{Patent}}_{ij} = \alpha _j + \beta _1D_{i = {\mathrm{T}}} + \beta _2D_{i = {\mathrm{T}}}X_{ps} + \beta _3D_{i = {\mathrm{T}}}X_j + {\it{\epsilon }}_{ij}$$

where *X*_*ps*_ is a set of design and operational features that vary across programs and sectors within programs; *X*_*j*_ is a set of firm and industry characteristics. To assist statistical inference, we also divided the ETS firms into two groups according to program specifications or firm characteristics to be investigated, and compared the DID values between groups based on Wilcoxon’s rank-sum test.

It has to be noted that, although the same matched sample from the main estimation is used, it does not represent the same identification strategy. The main estimation takes advantage of different program inclusion criteria to use matching to recover seemingly randomized assignment of ETS status for causal identification. But when making inference for the effect from different program features, assignment of these features is not likely made randomized through matching, because they may be correlated with program inclusion criteria.

## Supplementary information

Supplementary Information

Peer Review File

## Data Availability

The original patent data has been made available by the State Intellectual Property Office via its website (http://www.sipo.gov.cn/zhfwpt/zljs/) and also published by the Intellectual Property Publishing House Co. Ltd, with bulk access granted. The Annual Survey of Industrial Firms is confidential and restricted from public access. For reproducing figures and results, processed data with confidential information removed is available from the authors on request.

## Code availability

Computer code is available from the authors on request.
