# Supplementary material for: Low-carbon innovation induced by emissions trading in China
Source: Nat Commun. 2019 Sep 9;10:4088. doi: 10.1038/s41467-019-12213-6 (PMC6733790; doi:10.1038/s41467-019-12213-6)
Supplement: Supplementary file 1 — Supplementary Information [file 41467_2019_12213_MOESM1_ESM.pdf]

Supplementary Information for

"Low-carbon innovation induced by emissions trading in China"

by Zhu et al.

This file includes
Supplementary Figures 1 to 11,
Supplementary Tables 1 to 16,
Supplementary Notes 1 to 7,
and Supplementary References.

Supplementary Figures

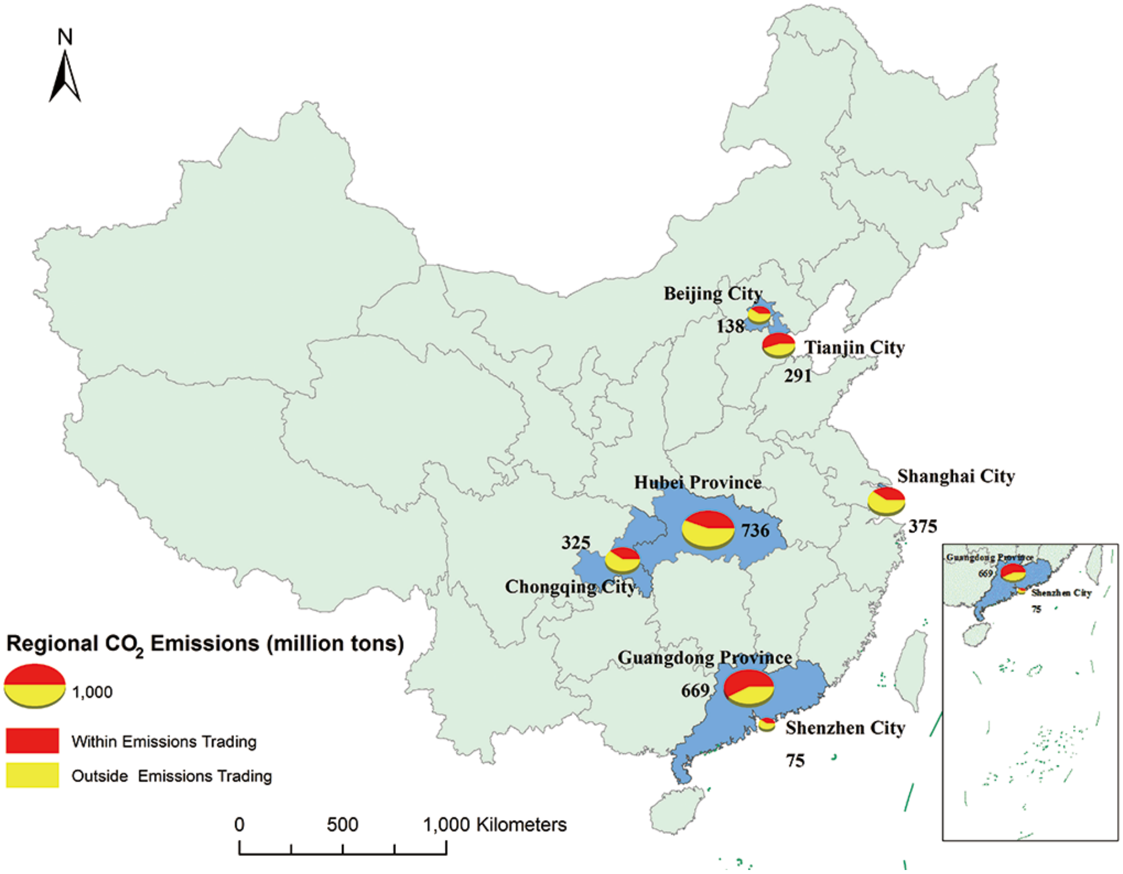

Supplementary Fig. 1: Seven regional carbon ETS programs in China. The numbers indicate regional total carbon emissions, and the pie charts indicate the percentage of emissions covered by the ETS (red color) as of the regional total emissions. Source: Authors' own creation, with emission data collected from the *China Carbon Market Report* by the National Center for Climate Change Strategy and International Cooperation.

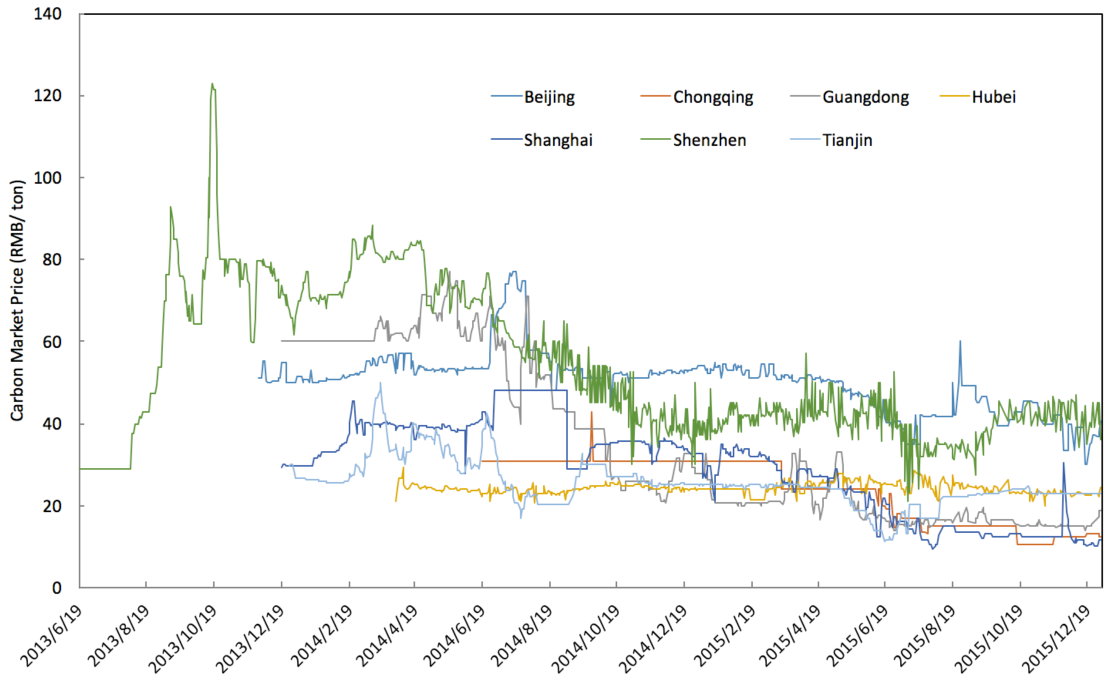

Supplementary Fig. 2: Daily average carbon price in seven emissions exchanges. Source: authors' own calculation based on individual transactions listed in "Carbon K Line".

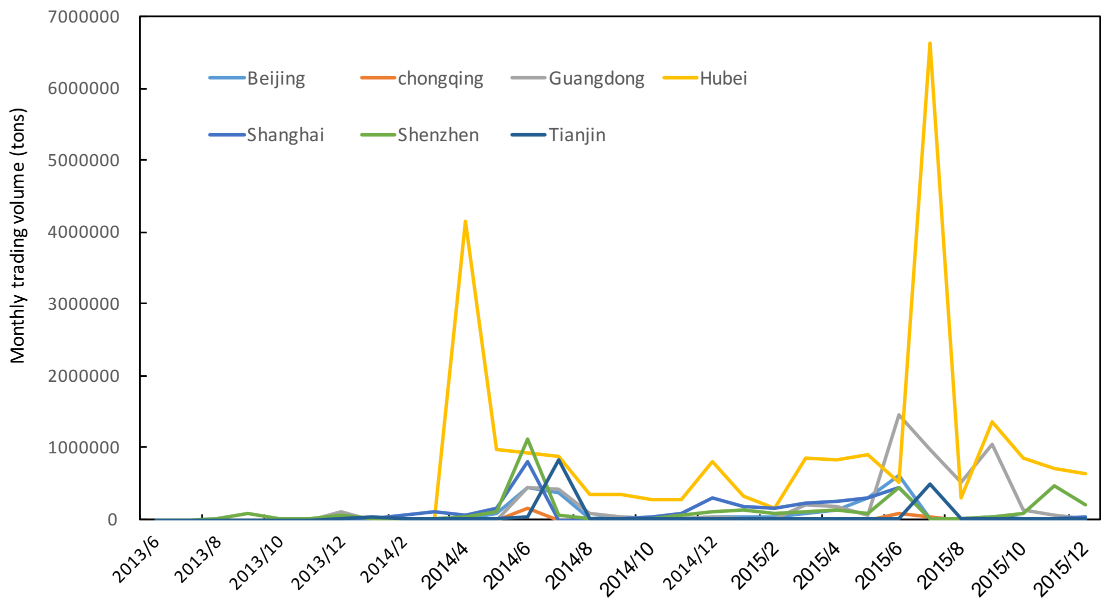

Supplementary Fig. 3: Monthly public trading volumes in seven emissions exchanges. Source: authors' own calculation based on individual transactions listed in "Carbon K Line".

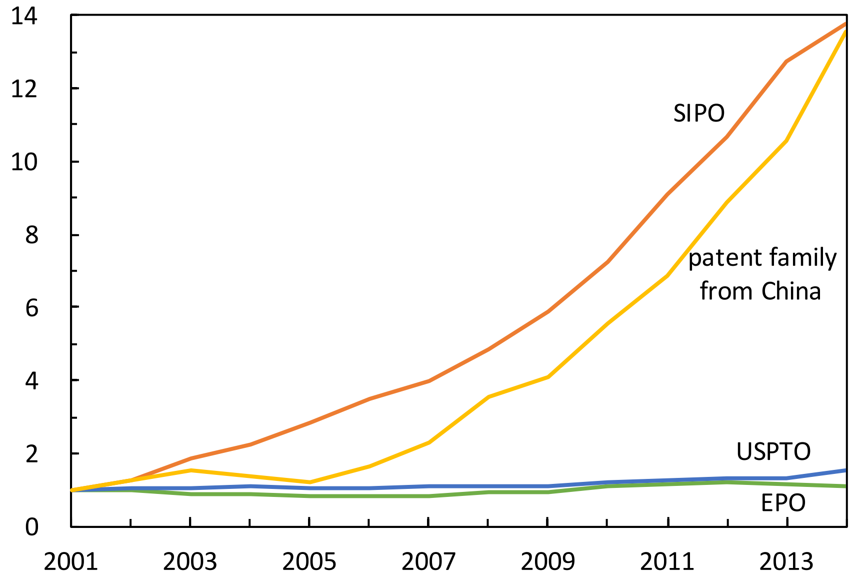

Supplementary Fig. 4: Changes of low-carbon patenting by patent offices and low-carbon patent family from China relative to 2001.

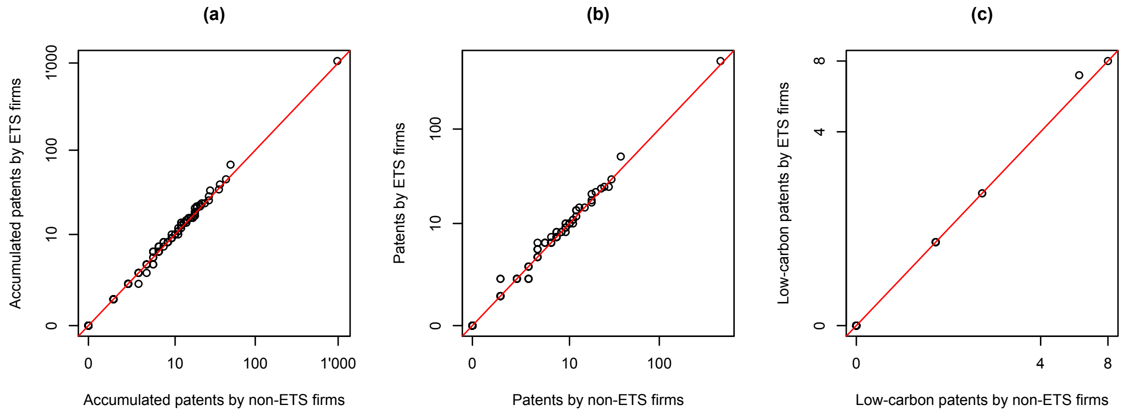

Supplementary Fig. 5: Quantile-quantile plots comparing the ETS firms and the matched non-ETS firms in (a) historic total patenting by 2012, (b) total patenting during 2011-2012, and (c) low-carbon patenting during 2011-2012.

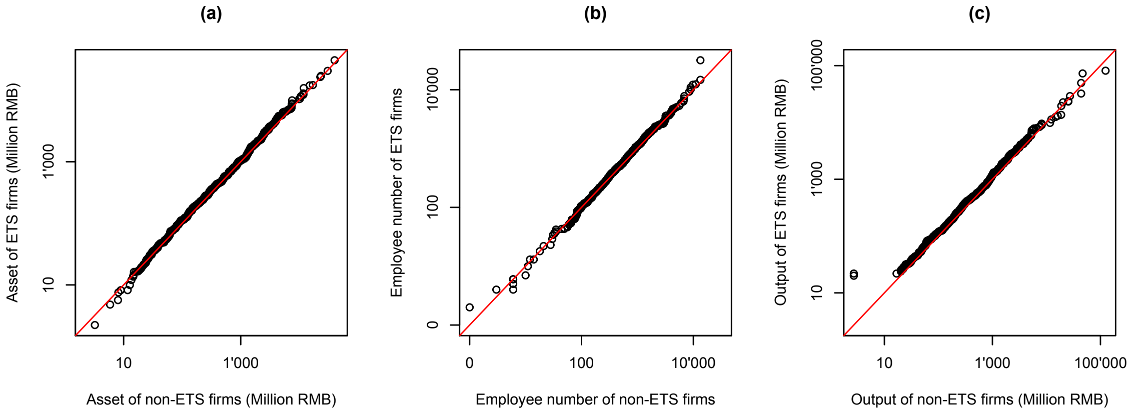

Supplementary Fig. 6: Quantile-quantile plots comparing the ETS firms and the matched non-ETS firms in (a) total asset, (b) number of employee, and (c) output (not used in matching) in 2012.

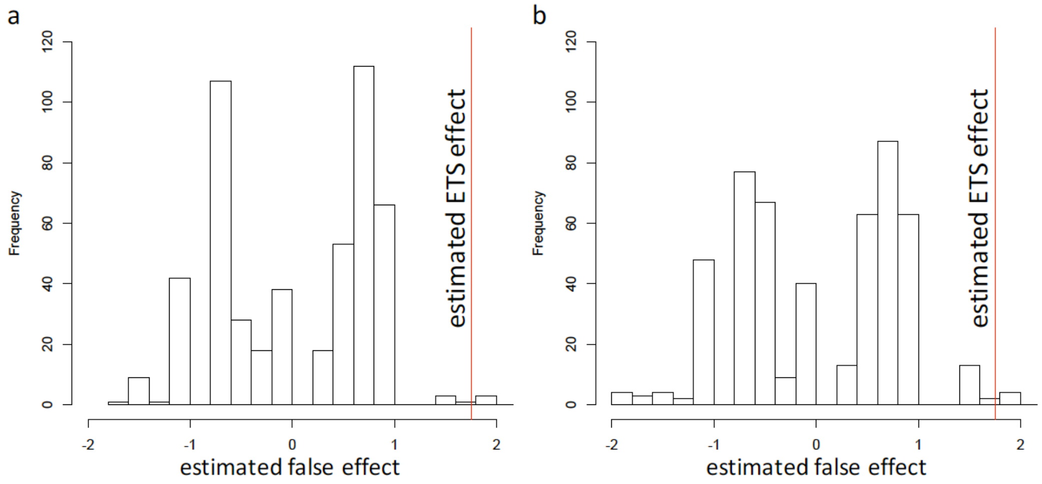

Supplementary Fig. 7: Distribution of estimated effects from placebo tests based on (a) non-ETS firms in EST sectors in low-carbon pilot regions and (b) non-ETS firms in EST sectors nation-wide.

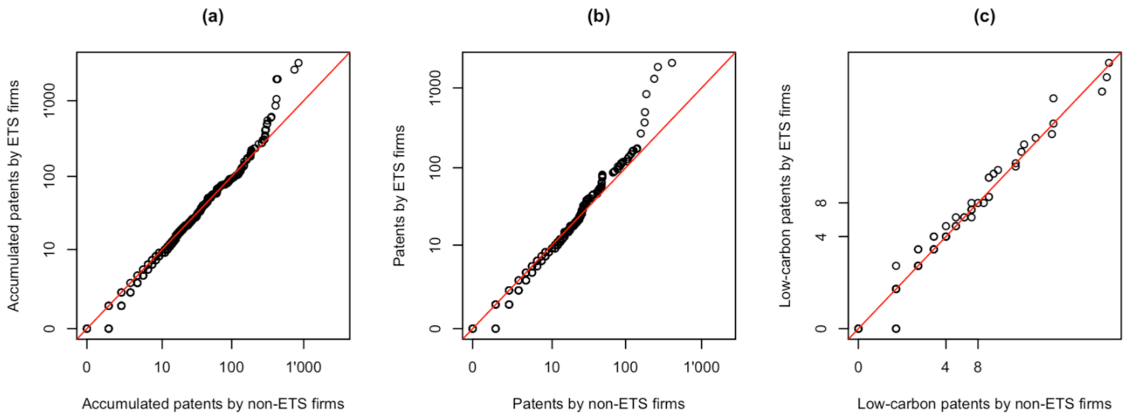

Supplementary Fig. 8: Quantile-quantile plots comparing the ETS firms and the matched non-ETS firms in (a) historic total patenting by 2012, (b) total patenting during 2011-2012, and (c) low-carbon patenting during 2011-2012, based on propensity score matching.

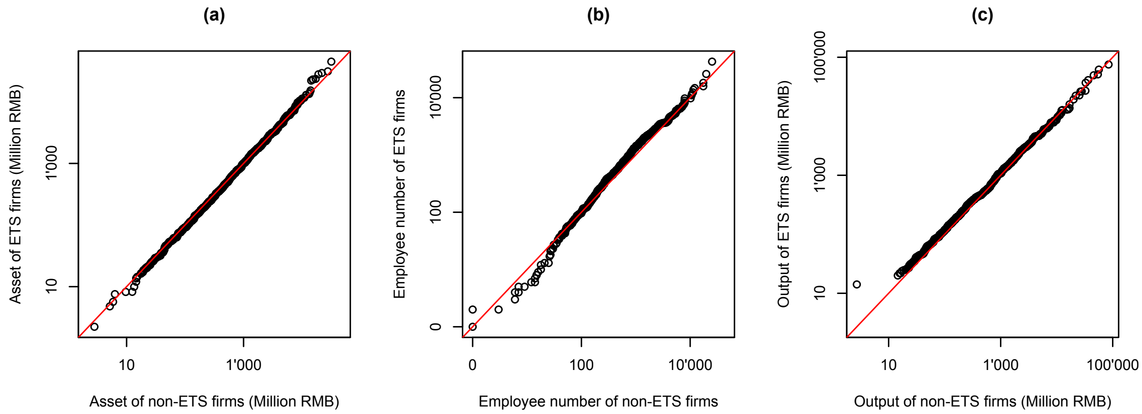

Supplementary Fig. 9: Quantile-quantile plots comparing the ETS firms and the matched non-ETS firms in (a) total asset, (b) number of employee, and (c) output (not used in matching) in 2012, based on propensity score matching.

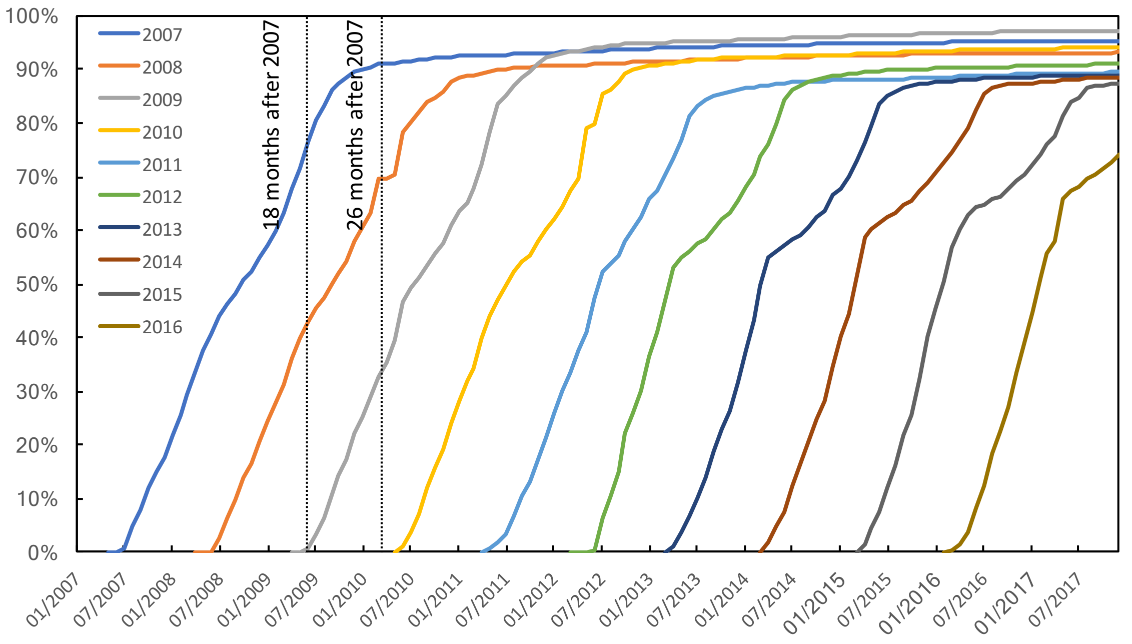

Supplementary Fig. 10: Cumulative distribution of patent publication month by annual application cohort.

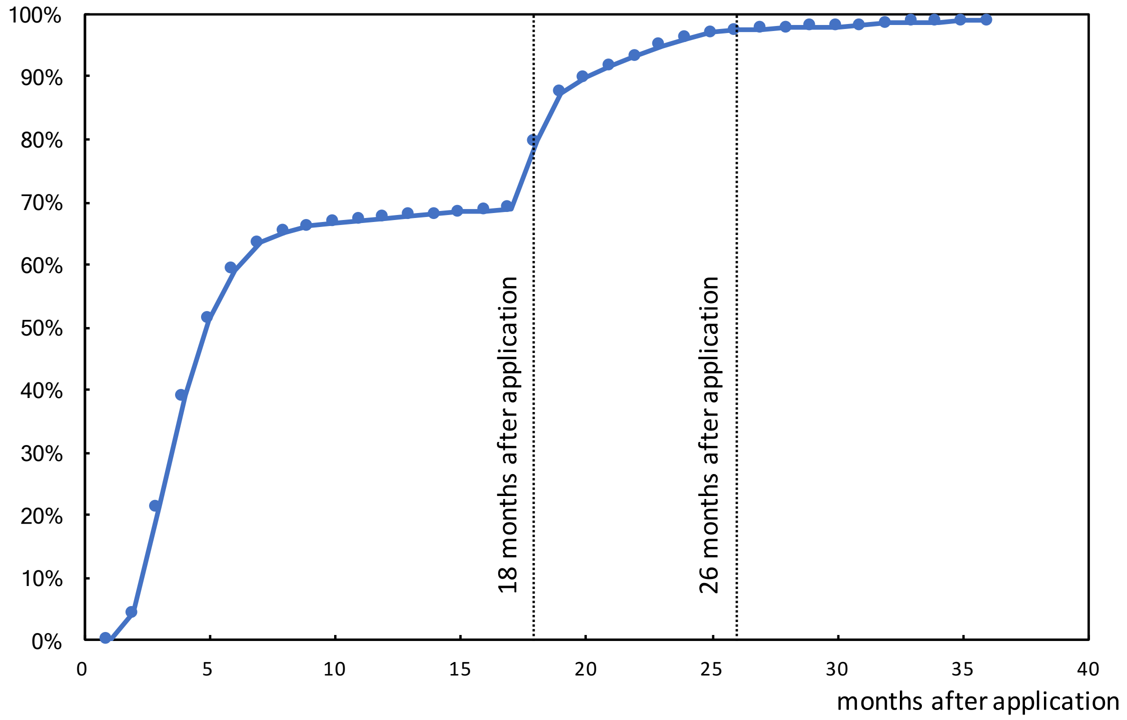

Supplementary Fig. 11: Aggregate cumulative distribution of patent publication month after application.

Supplementary Tables

Supplementary Table 1: Overview of the seven ETS programs during 2013-2015.

Market	Shenzhen	Shanghai	Beijing	Guangdong	Tianjin	Hubei	Chongqing
Date program started	06/2013	11/2013	11/2013	12/2013	12/2013	04/2014	06/2014
Inclusion criteria							
criteria announced [a]	06/2013	07/2012	11/2013	11/2013	02/2013	02/2013	05/2014
industrial sectors	26	10	10	4	5	12-15	6
emission (1000 ton CO_2)	>3	>20	>5-10	>20	>20	>150 [b]	>20
base year to measure emission	2009-11	2010-11	2009-12	2011-12	2009-12	2010-11	2008-12
Coverage							
of regional emissions (%)	40	40	40	58	55	44	40
number of firms	635-636	190-191	415-551	211-242	112-114	138-167	233-242
Allowance allocation							
annual total (million ton CO_2)	30	150	55	408	160	324	130
allocation with auction	No [c]	No [c]	No	Yes	No	Yes	No
benchmark [d]	sector	mixed	history	mixed	mixed	mixed	No
cap reduction commitment	No	No	No	partial [e]	No	13%	4.1%
rate or mass-based [f]	rate	by sector	by sector	by sector	by sector	by sector	mass
Market operation							
traded volume (million ton CO_2)	6.5	4.9	5.3	8.3	2.0	23	0.3
traded volume/annual allowance	0.22	0.03	0.10	0.02	0.01	0.07	0.002
auction (million ton CO_2)	N/A	N/A	N/A	15.2	N/A	2	N/A
average trading price (RMB/ton)	46	28	45	22	18	23	25
average auction price (RMB/ton)	N/A	N/A	N/A	51	N/A	20	N/A
Enforcement and compliance							
report emission (1000 ton CO_2) [g]	N/A	>10	>4	>10	>10	ETS	ETS
offset credit as of allowance	<10%	<5%	<5%	<10%	<10%	<10%	<8%
actual credit used (million ton)	2	>10	5	0.2	1.2	N/A	0
lump sum penalty (1000 RMB)	50-100	50-100	<50	50	by case	No	No
penalty per ton/ETS price	3	No	3-5	No	No	1-3	N/A
penalty in allowance reduction	Yes	No	No	Yes	No	Yes	No
penalty in policy preference	No	Yes	No	No	Yes	No	Yes
compliance rate (%)	99	100	97-100	99-100	97-99	81-100	70

Notes: a. Guangdong's inclusion criteria were initially announced in 2012, broadened in early 2013, and finalized in late 2013 with a narrower scope than the original plan. While for most ETS programs inclusion criteria were first set up without other policy specifications, in Chongqing the inclusion criteria were only announced after the policy was specified. b. The inclusion criteria for Hubei ETS was set at 60,000 tons of coal equivalent, converted into roughly 150,000 tons of CO_2 emissions to be comparable with other ETS programs. c. In an ad hoc manner, Shenzhen and Shanghai allocated some allowances

through auction in June 2014, i.e. the end of the first compliance cycle, to help a few firms comply in the first cycle. d. Programs that adopted mixed benchmarks to calculate allowances usually used sectoral benchmarks for power industries and new installations and own historical levels for other sectors; Beijing adopted sectoral benchmarks for new installations; in Chongqing firms first proposed their own allowance, which would be proved if in aggregate did not exceed the cap, and would be adjusted according to historical emission levels otherwise. e. Guangdong reduced the allowance cap for existing facilities but also reserved additional allowances for adjustment, and the total amount was not decreasing. f. allowances are pre-established in mass-based systems, and are updated according to the actual output in rate-based systems[1]; in mixed systems, allowances are usually rate-based for the power sector. g. Firms above the report emission level and below the inclusion emission level were not included in the ETS but required to report their annual CO_2 emissions; in Shenzhen there were alternative reporting criteria proposed, and in Hubei and Chongqing only ETS firms were required to report.
Source: Policy details are summarized based on policies announced by each ETS program. Statistics about market operation are from *China Carbon Market Report* by the National Center for Climate Change Strategy and International Cooperation, including not only public trading in the regional exchanges (as what we collected for Supplementary Figures 2-3) but also negotiated trading, which are not publicly recorded.

Supplementary Table 2: Summary statistics of the means and standard deviations of firm characteristics in the full sample and matched sample.

	firms from the sectors regulated by the ETS		matched sample (caliper=1.5)	
	ETS firms	non-ETS firms	ETS firms	non-ETS firms
2011-12 low-carbon patenting	1.81	0.14	0.03	0.03
	(18.27)	(3.04)	(0.39)	(0.39)
2011-12 total patenting	37.59	2.36	1.46	1.49
	(386.20)	(19.98)	(17.39)	(16.78)
historic low-carbon patenting by 2012	5.75	0.27	0.05	0.05
	(61.32)	(4.65)	(0.55)	(0.52)
historic patenting by 2012	126.05	4.77	2.83	2.91
	(1567.48)	(48.44)	(36.43)	(35.57)
2012 number of employee	1,702	342	870	792
	(5,330)	(1,251)	(1,672)	(1,197)
2012 total asset (million RMB)	2,652	239	1,174	1,010
	(11,900)	(2,166)	(2,859)	(2,691)
firm age by 2013	15.12	10.43	13.17	12.84
	(10.75)	(7.88)	(8.40)	(8.21)
2012 output (million RMB)	2,661	295	1,313	1,159
	(11,200)	(2,122)	(4,801)	(5,474)
2014-15 low-carbon patenting	2.10	0.17	0.14	0.10
	(17.59)	(3.98)	(0.91)	(1.17)
2014-15 total patenting	40.40	2.70	2.53	2.12
	(374.17)	(25.91)	(11.58)	(16.18)
observations	1,454	228,163	852	770

Supplementary Table 3: *t* tests for key variables after matching.

	difference in the means	standard error	p-value
2011-12 low-carbon patenting	0.0012	0.018	0.949
2011-12 total patenting	0.088	0.809	0.913
historic low-carbon patenting by 2012	-0.0023	0.025	0.926
historic patenting by 2012	0.175	1.703	0.918
2012 total asset (million RMB)	102	133	0.442
2012 number of employee	77.5	71.8	0.280
firm age by 2013	0.331	0.402	0.410
2012 output (million RMB)	75.6	250	0.762
four-digit industry sector	exactly matched		
low-carbon pilot region	exactly matched		

Note: Output is not used in matching.

Supplementary Table 4: Equivalence tests for key variables after matching.

	median difference	critical equivalence range	equivalence range
2011-12 low-carbon patenting	0	±<0.01	±0.075
2011-12 total patenting	0	±<0.01	±3.34
historic low-carbon patenting by 2012	0	±<0.01	±0.105
historic patenting by 2012	0	±<0.01	±7.02
2012 total asset (million RMB)	10.2	±29.7	±550
2012 number of employee	7	±25	±296
firm age by 2013	0	±0.49	±1.66
2012 output (million RMB)	40.3	± 53.0	±1031
four-digit industry sector	exactly matched		
low-carbon pilot region	exactly matched		

Note: Output is not used in matching.

Supplementary Table 5: Estimated ETS effects on the rate and direction of innovation during 2014-15.

	low-carbon patenting	other (non-low-carbon) patenting
Individual effect		
point estimate	1.75	1
95% confidence interval	(0.5, 1.9)	(0, 2)
Aggregate effect		
point estimate	66.5	146
95% confidence interval	(22, 71)	(0, 269)
Percentage effect		
point estimate	117.7%	7.7%
95% confidence interval	(21.8%,136.5%)	(0%, 15.3%)

Note: The aggregate effect was calculated by applying the individual effect to firms with consideration of a corner solution – filing zero patents under varying levels of intention and actual innovation – and used for calculating percentage effects and Fig. 3C.

Supplementary Table 6: Main results of ETS-induced low-carbon patenting under different matching specifications.

	estimated firm-level effect	95% confidence interval	observations treatment + control
caliper=0.5; treatment: control=1:1	1.5	(1, 5)	481 + 436
caliper=0.5; treatment: control=1:2	2	(1, 5)	394 + 678
caliper=0.5; treatment: control=1:3	1.75	(1, 2.9)	333 + 803
caliper=0.75; treatment: control=1:1	1	(0, 1.9)	621 + 562
caliper=0.75; treatment: control=1:2	1.5	(1, 1.9)	542 + 898
caliper=0.75; treatment: control=1:3	1.75	(1, 2.9)	480 + 1115
caliper=1; treatment: control=1:1	1	(0, 1.9)	721 + 649
caliper=1; treatment: control=1:2	1.5	(1, 1.9)	618 + 1043
caliper=1; treatment: control=1:3	1	(1, 1.9)	576 + 1344
main specification (caliper=1.5; 1:1)	1.75	(0.5, 1.9)	852 + 770
caliper=1.5; treatment: control=1:2	1	(1, 1.9)	765 + 1310
caliper=1.5; treatment: control=1:3	1	(1, 1.9)	727 + 1739

Supplementary Table 7: ETS-induced low-carbon patenting with different scopes of low-carbon technology.

	estimated firm-level effect	95% confidence interval	observations treatment + control
main specification	1.75	(0.5, 1.9)	852 + 770
narrow scope of low-carbon	1.75	(1, 1.9)	873 + 791

Supplementary Table 8: ETS-induced low-carbon patenting with a more restrictive exact match within ETS regions.

	estimated firm-level effect	95% confidence interval	observations treatment + control
main specification	1.75	(0.5, 1.9)	852 + 770
exact match on ETS region	1.75	(1, 2.9)	762 + 566

Supplementary Table 9: ETS-induced low-carbon patenting using alternative samples and baseline years.

baseline and sample	estimated firm-level effect	95% confidence interval	observations treatment + control
2011-2012	1.75	(1, 2.9)	762 + 566
2011-2012 without Tianjin ETS	1.75	(1, 3.9)	697 + 525
2011-2012 without four ETS	1.75	(0.1, 3.9)	391 + 332
2010-2011	1.5	(0.7, 1.9)	807 + 597
2012-2013	1.5	(1, 3.9)	744 + 563
2011-2013	1.2	(1, 2.9)	728 + 546

Supplementary Table 10: Alternative estimation for the main effect and spillovers.

	estimated firm-level effect	robust standard error
main specification	0.024**	(0.012)
spillovers: large firms in ETS sector	0.018**	(0.007)
spillovers: large firms in Shenzhen sector	0.022**	(0.010)

Note: ** indicates 5% significance level.

Supplementary Table 11: Placebo test of a false treatment in 2008.

	estimated firm-level effect	95% confidence interval	observations treatment + control
main specification (2011-2012)	1.75	(0.5, 1.9)	852 + 770
false treatment in 2008	-0.25	(-0.9,0.9)	1023+921

Supplementary Table 12: Comparing matched and unmatched firms in the main specification and matching quality of unmatched firms without a caliper.

	main specification (caliper=1.5)		matching unmatched in main specification (no caliper)	
	matched	unmatched	ETS firms	non-ETS firms
2011-12 low-carbon patenting	0.03	4.33	5.50	2.46
	(0.39)	(28.21)	(32.96)	(10.56)
2011-12 total patenting	1.46	88.71	116.45	28.01
	(17.39)	(596.41)	(701.18)	(109.48)
historic low-carbon patenting	0.05	13.82	17.58	5.79
by 2012	(0.55)	(94.75)	(110.95)	(23.83)
historic patenting by 2012	2.83	300.43	394.89	63.13
	(36.43)	(2,426.17)	(2,855.91)	(194.23)
2012 number of employee	870	2,880	3,181	1,907
	(1,672)	(7,897)	(8,848)	(7,692)
2012 total asset (million RMB)	1,174	4,743	5,592	2,719
	(2,859)	(17,953)	(19,015)	(12,922)
firm age by 2013	13.17	17.88	18.20	15.80
	(8.40)	(12.90)	(13.60)	(12.19)
2012 output (million RMB)	1,313	4,567	5,763	2,538
	(4,801)	(16,198)	(18,882)	(9,497)
2014-15 low-carbon patenting	0.14	4.86	6.14	2.61
	(0.91)	(27.09)	(31.62)	(14.24)
2014-15 total patenting	2.53	93.99	122.23	26.71
	(11.58)	(577.38)	(678.05)	(120.43)
observations	852	602	432	400

Supplementary Table 13: Inference for the aggregate effect on the whole sample.

	point estimate	95% confidence interval
For matched ETS firms		
individual effect	1.75	(0.5, 1.9)
aggregate effect	66.5	(22, 71)
percentage effect	117.7%	(21.8%,136.5%)
For all ETS firms assuming the same individual effect on unmatched firms		
aggregate effect	282	(90, 301)
percentage effect	10.13%	(3.04%, 10.94%)
For all ETS firms assuming no effect on unmatched firms		
aggregate effect	66.5	(22, 71)
percentage effect	2.23%	(0.73%, 2.38%)

Supplementary Table 14: Estimation and extrapolation based on propensity score matching.

	point estimate	95% confidence interval
For matched ETS firms		
individual effect	0.75	(0, 1.9)
aggregate effect	98.2	(0, 217.4)
percentage effect	8.08%	(0, 19.82%)
For all ETS firms assuming the same individual effect on unmatched firms		
aggregate effect	135	(0, 301)
percentage effect	4.63%	(0%, 10.94%)
For all ETS firms assuming no effect on unmatched firms		
aggregate effect	98.2	(0, 217.4)
percentage effect	3.33%	(0, 7.68%)

Supplementary Table 15: Estimated effect for each carbon market on low-carbon patenting.

	point estimate	95% confidence interval	matched treated+ control firms	caliper
reporting firms	1	(-1, 2.9)	128+126	1.5
top-10 firms in ETS sectors	1	(1, 1)	1,430+1,376	1.5
top 11-20 firms in ETS sectors	0.75	(0, 0.9)	1,387+1,337	1.5
bottom-10 firms in ETS sectors	0.75	(-0.9, 0.9)	2,142+2,029	1.5
large firms in Shenzhen sectors	1	(1, 1.9)	1,074+998	1.5
small firms in Shenzhen sectors	0.25	(-0.9, 0.9)	1,411+1,313	1.5
co-patenters of ETS firms	0.75	(-1, 3.9)	79+76	no
co-patenters of ETS firms	0	N/A	13+13	1.5

Supplementary Table 16: ETS-induced innovation effects on different types of low-carbon patenting.

Point estimation for two patent types			Wilcoxon's signed-rank test
	utility model	invention	alternative hypothesis
Point estimation	0.75	1.5	utility<invention
95% confidence interval	(-0.9, 1.9)	(0, 6)	p=0.399

Supplementary Notes

Supplementary Note 1 Carbon Emissions Trading as a Policy Experiment

China's pilot carbon emissions trading scheme (ETS) has been an important policy instrument for climate mitigation, for which comprehensive review has been done in the literature[2,3]. Here we briefly highlight the key features of the policy process, design, and market activities of the pilot ETS programs. They help to explain our research motivation for learning from the policy experimentation, main strategy for causal identification based on the policy experimentation, and research design for heterogeneous policy effects.

As the cabinet member of the State Council in charge of climate policies, the National Development and Reform Commission (NDRC) selected seven regions – both provinces and cities – as pilots in October 2011 to explore the ETS independently[i]. They were all part of the low-carbon pilot scheme. Different from previous policies which usually focused on energy efficiency across all provinces, the low-carbon pilot scheme was only explored in a few provinces and cities. It features policy experimentation, an important policy process in China's socio-economic development[4,5]: the national government solicited applications from provinces and cities, reviewed their climate mitigation targets and plans, and designated some provinces and cities as low-carbon pilot regions. Selected pilot regions were encouraged to explore innovative policy measures that could efficiently reduce CO_2 emissions. Successful experience would be later diffused to other regions.

The seven ETS regions are spatially dispersed (Supplementary Fig. 1), including four provincial-level municipalities (Beijing, Tianjin, Shanghai, and Chongqing), two provinces (Guangdong and Hubei), and one city in Guangdong province (Shenzhen). Although Shenzhen City is part of Guangdong Province, they set up two independent ETS programs. With great flexibility in policy design left by the NDRC, most of the pilot regions did not finalize their own rules until shortly before or even after the markets were formally launched[3]. The NDRC only announced the first guideline for carbon emissions trading in December 2014[ii], after all the regional ETS pilots had been launched. This process reflects the nature of the ETS as seven independent policy experiments based on the same policy instrument but different design. As an important policy learning process, it is expected to contribute to the development of a national carbon market.

---

[i] *Notice on implementing carbon emissions trading pilots*. Source: http://www.ndrc.gov.cn/zcfb/zcfbtz/201201/t20120113_456506.html.

[ii] *Interim rules on carbon emissions trading*. Source: http://qhs.ndrc.gov.cn/zcfg/201412/t20141212_652007.html

Because of the nature of independent policy experiments, great heterogeneity exists across ETS programs, as shown in Supplementary Table 1. One striking feature is the inclusion criteria, which supports our use of matching for causal identification. The ETS programs differ in firm inclusion criteria with regard to their industrial sectors (4 to 26 sectors), emission threshold (3 to 150 thousand tons of CO_2), and the base years to measure emission for inclusion (2009-11, 2010-11, 2009-12, 2011-12, or 2008-12). Two firms may be assigned to alternative statuses, one in an ETS program and the other outside, because of their differences in location, combination of sector and location, combination of location and emission, and the year in which they reached the emission threshold.

For example, many industrial sectors (and therefore firms in those sectors) being covered in the Shenzhen ETS are not covered in the Guangdong ETS; a firm in Hubei may be below the emission threshold and outside the ETS, but would have been in the ETS if it were in Chongqing; a firm may emit as many CO_2 as other ETS firms annually, but was below the emission threshold during the base years for inclusion measurement and outside the ETS. Matching helps to find for ETS firms these similar non-ETS pairs as their counterfactuals. More importantly, most ETS programs finalized their inclusion criteria only shortly before the programs started, and selected base years in previous periods without overlap for inclusion measurement. The time gap precluded self-selection of firms by manipulating their CO_2 emission levels to opt in or out of the ETS, although we still tested this in robustness checks.

Besides inclusion criteria and coverage, the seven ETS programs also differed in the way to allocate emission allowance. Allowance allocation can affect the level of innovation and therefore social welfare[6]. One important difference across ETS programs was that while most allowances were allocated for free, a small portion of allowances in Guangdong and Chongqing were allocated through auction, creating a higher incentive for climate mitigation. Hubei and Chongqing explicitly proposed *ex ante* commitment for reduced emission caps of the whole market, while others only required reduced emission intensity at most. Programs also differed in the benchmarks for allowance calculation and whether allowances are pre-established or updated according to actual output, i.e. rate or mass-based[1]. These allocation differences suggest different levels of policy stringency.

The operation of seven carbon markets was also different: some markets were clearly more fluid, characterized by more transactions than others; permits were sold at higher prices on average in these markets than others (Supplementary Figures 2-3). Depending on the market fluidity and price, firms may have differed in whether and to what extent they internalize carbon prices in a financial way, or take allowances as a binding performance mandate. These differences make comparisons of policy effects across program features valuable, especially for policy learning and development of the national carbon market.

Supplementary Note 2 Matching Quality

Supplementary Table 2 lists the summary statistics of the sample used in our research. The left columns show that ETS and non-ETS firms would not be directly comparable, even after we confined the non-ETS firms to be from the same four-digit industrial sectors as the ETS firms. The ETS firms were on average larger, older, and filing more low-carbon and other patents before the ETS started. After the matching process, the remaining ETS and non-ETS firms became much more similar in all these aspects, including their output not being used in matching. They only differed in their patenting activities after the ETS started.

We further confirmed in three ways that the matching process removed potential selection bias, making the assignment of ETS status more like a random experiment. The sample of treatment and control firms were nicely balanced after the matching process. First, we performed individual t tests for equality of means of key characteristics between the matched firm pair samples before the ETS. Supplementary Table 3 shows that the differences between means are very small and we cannot reject the null hypotheses that the means from the two samples are the same.

Second, we drew a quantile-quantile diagram of ETS firms and the matched non-ETS firms to compare their distributions of key characteristics. Supplementary Figure 5 shows that historic patenting by 2012, patenting during 2011-2012, and low-carbon patenting during 2011-2012 were quite similar between matched ETS and non-ETS firm pairs, lying on the line of y=x. Supplementary Figure 6 shows, in the same manner, that the distributions of pre-ETS total asset, number of employee, and output (not used for matching) were also quite similar between matched firm pairs.

Finally, we performed an equivalence test following Calel and Dechezleprêtre (2016)[7]. Supplementary Table 4 shows results of nice balance between the matched ETS and non-ETS firms for all the key characteristics again. The median differences of the covariates between the matched groups were either the same or similar. They were all contained in the critical equivalence ranges, which were 95% confidence intervals of the location shift parameter between the covariate distributions of the matched ETS and non-ETS firms, based on Wilcoxon's signed rank tests and Tobit modification for censoring at zero. Most importantly, the critical equivalence ranges all lay in the equivalence ranges of ± 0.2 standard deviations of the pooled sample's distributions.

Supplementary Note 3 Robustness Checks

This note demonstrates the robustness of our main results regarding ETS-induced low-carbon innovation over alternative research designs and specifications. These alternative approaches also help to evaluate the plausibility of our identification assumptions.

Alternative Matching Specification

We first explored alternative specifications in the matching process of our identification. Particularly, we tested using different calipers for quality control by dropping ETS firms whose Mahalanobis distance to the nearest non-ETS firms was longer than the caliper, and changed the number of non-ETS firms to be paired with a given ETS firm. We did not report specifications with a caliper of two or larger, which led to unbalanced samples. Supplementary Table 6 shows that all the point estimates were between one and two, and the lower bound between zero and one, mostly at one. Our conclusion of significant low-carbon innovation induced by the ETS on individual firm level is robust over all these specifications.

Alternative Scope of Low-Carbon Technology

Our main estimation adopted a relatively broad scope of low-carbon technologies based on the IPC Green Inventory. To test whether the estimated results remain in a key set of low-carbon technologies, we narrowed down the scope to low-carbon power generation and energy conservation in manufacturing. Waste reuse that may reduce primary energy consumption in production and low-carbon products – mainly technologies in transportation and fuel cell (which is classified as alternative energy production) in IPC Green are no longer included as low-carbon innovation. Supplementary Table 7 shows that our conclusion still holds in the smaller set of key low-carbon technologies. The point estimates are almost the same, while the 95% confidence levels are smaller, suggesting that the ETS induced innovation in these narrower set of key low-carbon technologies more consistently.

Exact Match in ETS Regions to Test Other Location-Specific Influences

Matching as an identification strategy assumes unconfoundedness, which is by its nature not testable in principle. However, we can try to eliminate potential confounding factors and evaluate the unconfoundedness assumption. One major factor that may cause potential confoundedness are policies other than the ETS. In our preferred estimation, we have required an exact match of firms' location within low-carbon pilot regions (all ETS programs are in low-carbon pilot regions), which could affect low-carbon innovation too.

Firms from the whole low-carbon pilot regions were used to reduce the chance of using firms within ETS regions, which could be affected by policy and knowledge spillovers.

Here we applied a more restrictive condition to require an exact match of firms' location within ETS regions. Although control firms would be more likely subject to spillovers, this could further eliminate any influence from location-specific factors. For example, the ETS regions might have enacted other policies complementary to the ETS programs to assist research and development. Policy influence from other low-carbon pilot regions that might compromise the quality of the control group could also be eliminated. Supplementary Table 8 shows that the more restrictive condition in the matching process reduced the number of matched pairs for estimation, but the point estimation remained the same. The lower bound estimation increased to one, and the upper bound increased, too. The conclusion of significant low-carbon innovation induced by the ETS remains valid, if not stronger.

Alternative Samples and Baselines to Test Unobservable Selection Bias

Given an exact match within ETS regions to eliminate potential location-associated influences, we further tested the potential influence to the estimation from firms' self-selection or government selection with regard to the ETS. Firms with different innovation potential might opt in or out of the programs by manipulating their CO_2 emission levels, which could generate the estimated effect. On the other hand, ETS programs might manipulate inclusion criteria that would more likely pick up firms with more innovation potential.

All the ETS programs announced inclusion criteria and then used firm emissions in prior periods for inclusion decisions. So it was technically infeasible for firms to opt in or out of the programs by changing their emission levels, unless they knew the inclusion criteria years before the formal announcement through information leakage. Therefore, we first dropped ETS firms from Tianjin, which had the shortest time gap between criteria announcement and the selected base years and most likely affected by information leakage, if any. The estimated effect remained unchanged (Supplementary Table 9). We further dropped ETS firms from three other programs (Shanghai, Beijing, and Guangdong) with the time gap less than one year. The point estimate remained the same and significant, although the confidence interval increased due to a much smaller sample (Supplementary Table 9).

If the government knew firms' potential innovation capability precisely and managed to set base years and emission threshold accordingly, it could also affect the estimated results. This assumption seemed extremely unlikely, especially after considering our results in Table 2 where firm features – such as size and previous innovation experience – were uncorrelated with the induced innovation effect. But we tested it here anyway by changing the baseline years used as the pretreatment period in our matching process – difference in

potential innovation capability could be more or less captured by patenting statistics in an earlier, later, or longer period. The results are robust in alternative baseline settings, too.

Alternative Estimation Method

We estimated the main effect and spillovers from the ETS based on a more commonly used DID estimation method as in Equation 2. Supplementary Table 10 shows that both the main effect on ETS firms and spillover effects on non-ETS firms remained significant based on the alternative estimation method.

Placebo Test of Other Potential Confoundedness

Two sets of placebo tests were designed to test any other potential confoundedness. First, we tested again whether the estimated effect was not driven by the ETS but by unobserved features of the regions, industries, or firms, by assuming a false treatment in 2008. Corresponding to our main specification, two years before and after 2008 were set as the pretreatment and posttreatment periods, respectively. Supplementary Table 11 shows that a small, nonsignificant effect was estimated for the false treatment, suggesting that the effect was not driven by the regional, industrial, or firm features.

Second, we explicitly tested the possibility of the estimated effect being caused by any other omitted variable or simply by chance. A placebo test was conducted by assigning false ETS status to 1,460 randomly selected firms (similar to the actual number of ETS firms in our sample) in four-digit sectors covered by the ETS but outside ETS regions. We matched these false ETS firms with other firms outside ETS regions and estimated the treatment effect based on our main matching and estimation method. We used both ETS sectors in low-carbon pilot regions (consistent with our matching specification) and ETS sectors nation-wide (with more variance introduced) as the pool for the placebo tests; tests in both pools were repeated 500 times to avoid making inference based on rare events. Supplementary Fig. 7 shows that the induced innovation effect was very unlikely produced by any omitted variable or chance: the effect was reached or exceeded only five times (six times on both sides considering absolute value) out of 500 times of placebo tests in low-carbon pilot regions; it was reached or exceeded seven times (14 times on both sides considering absolute value) out of 500 times of placebo tests nationwide.

Supplementary Note 4 Unmatched Firms

Based on our matching estimator, the conclusion was drawn upon 852 out of 1454 ETS firms, about 60% of our sample. It would be important to discuss whether the result of ETS-induced low-carbon innovation applied to the other 602 firms. The left columns of Supplementary Table 12 shows that the unmatched ETS firms were much bigger and older than the matched ETS firms, and filed substantially more low-carbon and other patents

historically and before the ETS. The right columns of the table show that without a caliper for the nearest neighbor matching estimator, we could match more than two thirds of the unmatched firms with non-ETS firms based on the same exact match condition (four-digit sector and low-carbon pilot region). But the quality of matching was not good for any inference – the matched firm pairs differed in all dimensions.

Instead of estimating and drawing conclusion based on low-quality matching, Supplementary Table 13 makes inference for the aggregate effect on the whole sample based on more reliable estimation of the matched sample. If we assume that the ETS effect on individual unmatched firms was the same as that on the matched firms of 1.75 additional low-carbon patents, the aggregate effect on the whole sample would be 282 additional low-carbon patents, or 10% increase. If we assume an extreme case of no policy effect on unmatched firms, the aggregate effect on the whole sample would be only 66.5 additional patents, representing an increase of 1.9%. Although we cannot generalize the estimated effect beyond the matched sample, we can conclude that the aggregate policy effect on the whole sample of ETS firms is statistically significant yet limited.

Supplementary Note 5 Results from Alternative Matching Method

Although our nearest neighbor matching method led to a high-quality, balanced sample of matched firm pairs, it only kept 60% of ETS firms in our sample. To make the matched firm sample more representative, we adopted an alternative method of propensity score matching, which was used for evaluation of the EU ETS[7]. We used all the same variables and combinations of variables that were used to calculate the Mahalanobis distance in nearest neighbor matching to calculate propensity scores, conditional on the same exact match condition, with a caliper of 0.01. It improved the representativeness of the matched sample by covering 91%, or 1325 out of the 1454 ET firms. Supplementary Figures 8-9 shows that, however, the quality of matching became lower, particularly for large, more innovative firms, which consistently filed more patents than their matched counterfactuals historically and right before the ETS started. To a lesser extent, smaller, less innovative firms were not as closely matched for their patenting as by nearest neighbor matching. The result echoes that from nearest neighbor matching, which dropped these large, more innovative firms due to the lack of high-quality counterfactuals.

To confirm whether the induced innovation effect pertain to all the ETS firms, we made the same Tobit-modified empirical-likelihood-based difference-in-differences estimation for the matched sample anyway. Supplementary Table 14 shows that the point estimate for the individual effect on a single firm was smaller but still significant. Because most of the ETS firms remained in the matched sample, our extrapolation led to closer results of aggregate and percentage effects under different assumptions. The ETS facilitated the 91% matched ETS firms to file 8% more low-carbon patents, and all the ETS firms to file 4.6%

more patents assuming the same individual effect of 0.75 additional patents on all firms, or 3.3% more assuming no effect on the unmatched firms.

Supplementary Note 6 Spillover Effects

While the main results for spillovers are demonstrated in Table 1 of the manuscript, we explain some details here. Large and small non-ETS firms in ETS sectors were defined as the top-10 and bottom-10 firms by output. Because spillovers extended at least to top 11-20 firms in those sectors too (Supplementary Table 15), we could not make a very accurate estimation of the aggregate spillover effects.

Large non-ETS firms by output in non-ETS sectors of six ETS regions but covered by the Shenzhen ETS were selected for each sector in each region as the same number of ETS firms covered in the same four-digit sector in Shenzhen. We did not stick with top-10 here because it would lead to too many firms: there were more than 200 four-digit sectors in the Shenzhen ETS and, when multiplied by ten firms in each sector and six programs, it would generate more than 10,000 firms to be matched and estimated. Small firms were selected in the same strategy.

While most of the estimation was based on a caliper of 1.5 in matching, the same as used in our main estimation, we reported the estimation for co-patenters with no caliper in matching. If a caliper of 1.5 were used, only 13 co-patenters would be left and the 95% confidence interval could not be estimated.

Finally, in the review process, one reviewer raised competing explanations of the spillover effects as for competition and patent transfer instead of policy spillovers. The competing explanations were ruled out in our response to the comments with support of statistical evidence.

Supplementary Note 7 Innovation Quality

Except all the analyses of the impact of program specification and firm characteristics on induced innovation shown in the manuscript, we also tested whether patent quality was affected. Lack of patent citation information from the SIPO data, we made point estimation of the ETS effects on the high-quality invention patents and on the low-quality utility models in Supplementary Table 16. We also did paired comparison between the difference-in-differences of invention patents and that of utility models for each firm pair, based on Wilcoxon's signed-rank test. Although the point estimation was higher for high-quality invention, the DIDs of the two patent types were not statistically significantly different.

Supplementary References

1 Goulder, L. H. & Morgenstern, R. D. China's Rate-Based Approach to Reducing CO2 Emissions: Attractions, Limitations, and Alternatives. *AEA Papers and Proceedings* **108**, 458-462, doi:10.1257/pandp.20181028 (2018).

2 Zhang, D., Karplus, V. J., Cassisa, C. & Zhang, X. Emissions trading in China: Progress and prospects. *Energy Policy* **75**, 9-16 (2014).

3 Zhang, J., Wang, Z. & Du, X. Lessons learned from Chinas regional carbon market pilots. *Economics of Energy & Environmental Policy* **Volume 6** (2017).

4 Heilmann, S. Policy Experimentation in China's Economic Rise. *Studies in Comparative International Development* **43**, 1-26, doi:10.1007/s12116-007-9014-4 (2008).

5 Heilmann, S. From local experiments to national policy: The origins of China's distinctive policy process. *The China Journal* **59**, 1-30 (2008).

6 Fischer, C., Parry, I. W. H. & Pizer, W. A. Instrument choice for environmental protection when technological innovation is endogenous. *Journal of Environmental Economics and Management* **45**, 523-545, doi:https://doi.org/10.1016/S0095-0696(03)00002-0 (2003).

7 Calel, R. & Dechezleprêtre, A. Environmental Policy and Directed Technological Change: Evidence from the European Carbon Market. *Review of Economics and Statistics* **98**, 173-191 (2016).
