# Supplementary material for: Low-carbon innovation induced by emissions trading in China
Source: Nat Commun. 2019 Sep 9;10:4088. doi: 10.1038/s41467-019-12213-6 (PMC6733790; doi:10.1038/s41467-019-12213-6)
Supplement: Supplementary file 2 — Peer Review File [file 41467_2019_12213_MOESM2_ESM.pdf]

Review of *Emissions trading induces low-carbon innovation in China, but not yet by pricing*

December 5, 2018

Disclosure

As I have informed the Editor, I recently reviewed a version of this manuscript for another journal. I have updated my report to reflect changes in the manuscript, although the authors will recognise many of my comments.

Summary

This paper estimates the effect of China's seven pilot ETS programs on low-carbon innovation, applying the same methods that has been used to study the effect of the EU ETS (Calel and Dechezlepretre, 2016, REStat). The paper also compares estimates across the pilot programs to see what specific design features, if any, are driving the effects they observe. The authors conclude that effect is strongest among programs that use a mass-based emissions cap, and suggest that China's new national carbon market might induce more innovation if it switched from a rate-based to a mass-based cap.

Major comments

1. **Engage with the literature.** This paper follows similar studies of EU ETS, as well as one recent paper that also examines the impact of China's pilot programs on low-carbon innovation (Cui et al. 2018, AEA P&P). This paper should engage in a more direct conversation with that literature, if only to help the reader understand how it builds on, adds to, and sometimes reaches contradictory conclusions to that literature. For instance, on line 36 the authors claim to "present the first firm-level evidence of policy effects directly of ETS pilots on low-carbon innovation..."

but then on line 52 cite two papers (including Cui et al.) that have used firm-level data to provide evidence of the effects on low-carbon innovation. They seem quite dismissive of this previous work, but it's unclear why. As the reader, I want to understand what you're doing differently, and how your findings reinforce or contradict those earlier studies. In particular, Cui et al. find that pilots with a higher carbon price induced a greater innovation response, which you argue hasn't happened. I want to understand why your results are different. Are you perhaps able to replicate their finding, and then show how the result goes away when you take some other factors into account?

2. **Explain the source of variation in treatment.** A matched difference-in-differences design can reveal a causal effect only if there is a source of variation in treatment that, for some subset of companies, is credibly uncorrelated with unobserved drivers of future innovation outcomes. As currently written, the paper does not explain to the reader why some companies are regulated under a pilot program, while other fundamentally similar companies are not. Without a persuasive explanation I will tend to presume that the unregulated companies are not in fact similar. To the extent that you're matching on observable characteristics, you would only ensure greater dissimilarity on unobservable characteristics.

   There is a paragraph on this in the first section of the SI where you explain that "Two firms may be assigned to alternative statuses, one in an ETS program and the other outside, because of their differences in location, combination of sector and location, combination of location and emission, and the year in which they reached the emission threshold." As best I can parse this statement, there are three different identifying margins: (1) some matches will be identical in every respect except their pre-treatment location, (2) some matches will be identical in every respect except their pre-treatment sector, (3) some matches will be identical in every respect except their pre-treatment emissions. Is this right? Each one tells a qualitatively different story about identification, but the paper doesn't make clear to what extent you are relying on each of these margins. Then in Table S3, it seems you're actually matching exactly on 4-digit industry sector and pilot region, which seems to leave only the pre-treatment emissions margin. So in reality, you seem to be systematically comparing similar heavy polluters with lighter polluters, and implicitly asserting that heavy polluters are no more likely to innovate in the future, conditional on the set of covariates. Please state this simply and clearly. There is no reason to make it difficult for the reader to figure this out (if this is

indeed right?). It is essential that you clearly explain this in the main paper, too, not just in the SI.

The source of identifying variation is then somewhat different when you are estimating spillovers. In this case, 'treatment status' depends on both the ETS/non-ETS quasi-randomisation (discussed above) AND on the properties of the networks. If some firms are more prolific co-patenters, for instance, they are more likely than average to be assigned to 'treatment' when a random firm is assigned to the ETS. These network properties alter the permutation probabilities and the standard errors.

The source of identifying variation is again different when you're analysing effect heterogeneity across programs. In that case you're not comparing an ETS-firm with a non-ETS firm that was just below the emissions threshold. Either you are matching ETS-firms to non-ETS firms from other pilot programs, or perhaps you are not matching at all? Either way, you seem to be using regional differences in design rather than within-region thresholds. This has two consequences. First, it's harder to think about the regional variation in design as plausibly randomised. Second, even if it is quasi-random, you have to think harder about how you permute treatments to get your standard errors. If you're imagining swapping out the 'Beijing'-treatment for the 'Shanghai'-treatment for one Beijing-based firm, can you do that without simultaneously doing the same swap for all Beijing-based firms? Block randomisation of this sort would radically reduce the number of permissible permutations of the treatment vector and inflate your standard errors. I think some version of this exercise is interesting, and absolutely essential to this paper, but the authors should be careful to signal that the claims about causal identification are quite different and a bit weaker here.

3. **Changing the match.** The matching methodology and the resulting matches appear to have changed since the original submission. They authors seem to have changed the set of covariates substantially: adding total assets, employment, and some quadratic terms, applying log transformations, matching exactly for pilot region, and dropping output. I would like to see some kind of discussion for why they thought it was important to match on some things and not on other things. Remember that you're matching to balance potential outcomes, so what is the evidence and argument that these particular covariates are good predictors of potential outcomes in the absence of an ETS?

   Setting these crucial clarifications aside, an even bigger concern is that the set

of matched companies is now completely different from the original submission. You are now choosing how and who to match after having peaked at their outcomes. This lays you open to the risk that your matches are either consciously and unconsciously influenced by the estimates they produce. I think it's really important that your main analysis be based on the original method and sample. You should definitely include analysis based on this alternative matching method, but it should be included as one of your robustness checks. The distinction is important. What I ask, and asked before, is that you *report* the imbalances on all covariates, whether or not they were inputs into the matching algorithm. But the authors seem to have taken the opportunity to match on these extra variables in order, presumably, to reduce imbalances for the original matched sample that weren't initially reported. The results are ultimately not wildly different, so I don't understand the reluctance to report the original estimates.

4. **Relevance of unmatched ETS companies.** As the authors say, 40% of ETS firms are set aside because they couldn't find suitable matches, and these 40% are typically more innovative. My concern is that if the effect is substantially different in this group of companies, it completely changes the quantitative and qualitative conclusions of the paper. Even a small negative effect might be enough to cancel out the effect you observe in the matched sample, for instance.

   I appreciate that you've done a bit in the SI to look at the relevance of the many unmatched ETS firms. It would be helpful to also include some kind of bounding exercise, since it would provide a cleaner answer to the question of what kind of influence these omitted ETS firms could theoretically have on your estimates without having to compare them to a bunch of unsuitable controls. I think it's important that the results of this kind of bounding analysis are reported in the main results section of the paper, since it gives the reader context for interpreting the level of uncertainty in your conclusions beyond the statistical significance. You need to be more up front about the fact that you're looking at an unrepresentative subset of about 60% of ETS firms, and in particular, how sensitive your overall conclusions are to what effect the pilots might have had on the other 40%.

5. **Interpretation of spillover estimates.** I like the general approach to spillovers, but I think the description of the nature of possible spillovers is unnecessarily restricted, as is the interpretation of your findings. The authors speculate that non-ETS firms might be responding to the expectation of being under the ETS in the future. This is one valid hypothesis (although if firms are responding to

expectations, I think you need to address the question of how much you are underestimating your main effect because the ETS firms started responding before the ETS started). But spillovers can come in other shapes, e.g. non-ETS firms could respond to an ETS by reducing their innovation in the expectation that ETS firms will bear more of the innovation-burden, or non-ETS firms could respond by increasing their innovation to keep up with their more innovative ETS competitors, or by increasing their innovation to sell to ETS companies who now have greater demand for these technologies, etc. Each hypothesis potentially implies a different set of empirical patterns, and you would want to conduct an empirical test that discriminates between them. When you're just highlighting one hypothesis, it isn't clear whether your findings actually favour your hypothesis over the others.

In a similar vein, you basically find that a bunch of large non-ETS firms are increasing innovation. You jump to the conclusion that that's because they expect to be regulated under an ETS in the future, but I see no particular justification offered for this interpretation. An alternative interpretation, also consistent with your findings, is that something altogether different is going on in the Chinese economy that is driving low-carbon innovation among both large ETS and non-ETS companies. In this interpretation, perhaps most of the effect you're attributing to the ETS isn't actually because of the ETS at all. Basically, I think spillovers are a really challenging topic to deal with. Although I like the general approach the authors take, I think they have not done the hard work necessary to show why their estimates should be interpretated as they have, rather than, say, interpreted in a way that undermines their main estimates.

6. **Title.** I think the title needs to be changed. The phrase "not yet by pricing" is ambiguous, and as my comments above indicate, you don't really have a strong identification strategy that rules out pricing. Perhaps a phrasing focusing on your positive findings might be more appropriate: "Mass-based emissions trading induces low-carbon innovation in China".

Minor comments

1. line 8: "significantly induced low-carbon innovation" suggests statistical significance, rather than substantive significance. Perhaps "induced significant low-carbon innovation" instead.

2. line 10: "with policy expectation" suggests that 'policy expectations' is something

you're actually measuring. Better to describe what you've done so the reader can assess if they think it's a persuasive test of your hypothesis.

3. line 32: You talk about the pilot programs at "independent." This word has a statistical meaning that is inapproriate for this context (see earlier discussion on sources of variation).

4. line 36: This is where you assert that you are the first to present firm-level evidence on this question, before offering some unclear distinction with previous work on line 50. Please write this is a more engaged, clear, and less territorial way.

5. line 71: The authors are using the term "patent families" when they actually mean "triadic patents."

6. lines 109-114: For most of these robustness tests, it is clear what the authors conclude but totally opaque from the text what actual test has been performed. Please consider re-writing.

7. line 121: Again, "If an alternative matching method is used..." In what way is it different from the main matching method? The reader doesn't know how to make heads or tails of this paragraph without consulting the SI.

8. Figure 3.b.: A bar chart (rather than a pie chart) would allow you to also show the total magnitude of the induced innovation as well as confidence intervals.

9. line 146-150: If non-ETS companies innovate because they expect to become ETS companies in the future, that means your control companies are also increasing innovation and "... the individual effect of ETS firms would be greater than the estimation above." But it also means that the aggregate effect further inflated because the population of treated companies is larger than the set of ETS companies.

10. lines 154-168: If these groups of non-ETS companies are potentially contaminated controls, have you re-matched and re-estimated your main results when these contaminated firms are removed from the matching pool? How does this affect your results? Does it affect it in the way that you'd expect, given the hypothesised sign of the spillover?

11. Table 1: The last column lists the number of matched pairs, but not how many companies you started out trying to match in each category. The scope for selection bias is very different if you're matching 1% or 100% of companies in each category.

12. lines 207-222: Given my major comments about the source of identifying variation, you should be more careful in using causal language here like “reason” and “effect.” At least, you haven’t yet made a persuasive argument why these associations should be causally interpreted.

13. Table 2: It is unclear if the dependent variable, Δ low-carbon patent, is referring to a difference between firms or a change over time. Or is it a DID perhaps?

Reviewer #2 (Remarks to the Author):

This is an excellent paper that brings together a unique dataset to analyse the effect of China's pilot Emissions Trading Systems on low-carbon innovation. It is the first paper to robustly evaluate the impact of China's carbon markets on low-carbon innovation, which has high scientific importance and huge policy implications given the recent launch of the nation-wide carbon market in the country and its future expansion to sectors beyond energy production. The conclusions are backed by solid evidence. The data is of high quality and the econometric analysis is well conducted.

The paper presents three main results: (i) China's pilot ETS induced innovation in low-carbon technologies, as measured by patent filings in relevant technological fields, among the set of regulated companies; (ii) this effect was not associated with a decrease in patenting in other technologies (actually, the opposite is found); (iii) unregulated firms also reacted by filing more low-carbon patents, probably in the expectation of future regulation; and (iv) the impact was not found to be statistically greater in markets with higher carbon prices.

I think that the first 2 sets of results above are extremely solid. Result (iii) is very interesting, but I wonder how balance can be achieved if you match the largest (or top 10) firms in every ETS or Shenzhen sectors. If you systematically match these firms with smaller, could this not drive your results? Please provide also balance tests for this set of results, as the assumptions behind using matching are less obviously met given that you cannot exploit the same inclusion criteria.

My main reservation regards result (iv). This finding comes from a regression where the ETS status is interacted with multiple programme design characteristics as well as firm characteristics. Four reasons may explain the lack of statistical significance for the interaction term between ETS and the price, and for other interaction terms more generally: first, I expect many of the programme design features to be highly correlated with each other (a correlation matrix in the supplementary material would be welcome to check whether this is the case); second, I expect many of these programme features to be correlated with firm characteristics, but I couldn't find the list of firm characteristics included as controls (in particular, I wonder whether the statistically significant difference between mass-based and rate-based is not driven simply by a greater effect among manufacturing firms compared to firms in the energy sector. Do you have sector dummies and region dummies as controls?); third, there is little variation to exploit since the average price did not differ much across ETS programs (with the exception of Shanghai and Beijing); fourth, what matters for innovators is the (unobserved) expected future price of carbon on the market, not the current spot price.

For these reasons, and unless you can make the finding of an absence of a price effect more robust, I would make this result less prominent (in particular, not feature in the paper's title), as I think it is not that strongly supported by the analysis. For a wide-audience journal like Nature

Communications, the main result on the impact of the Chinese ETS on low-carbon innovation is sufficient anyway in my opinion.

Some less important comments follow:

1. It would be interesting to know how the pilot schemes were selected among potential candidates and what are the specificities of the chosen regions and cities compared to the rest of China, in terms of carbon emissions, emissions intensity and low-carbon innovation prior to the launch of the programmes. This would give some indications as to whether the results might generalise to the whole of China.

2. Your analysis of the impact on non-ETS firms is extremely interesting, but could you give an indication as to how this effect compares with the effect on the set of regulated firms, in terms of the number of total patents? It seems that the total effect on unregulated firms might be greater than the total effect on ETS firms.

3. That ETS programs also affect unregulated companies has implications, as you note, for the baseline results, which might be underestimated. Can you give an indication on the size of this bias? In particular, are 'large firms in ETS sectors' used as controls in the baseline results? Does the baseline treatment effect increase if you restrict control firms to firms less likely to be regulated in the future?

4. The merging between SIPO and ASIF only allows you to match 2 million patents out of the 8 million patents filed by firms. The ratio for low-carbon patents is similar (147k patents against 546k). Your sample is large enough, and I doubt that the matching quality would vary systematically around the inclusion threshold, but can you say more about the matching quality and the potential consequences for your analysis?

Reviewer #3 (Remarks to the Author):

This article uses patent analysis to determine the innovation impact of the seven ETS pilot schemes in China. Methodologically, it builds on comparable studies conducted for the EU ETS – a quasi-experimental design matching comparable ETS and non-ETS firms. The paper finds a positive innovation impact of the pilot ETS, increasing low-carbon innovation by a few percent (even for firms who might be expecting to be included in the ETS in the future). The analysis also demonstrates that the (free) allocation method matters, as only mass-based allocation is associated with a positive innovation impact. This is a critical finding as the national ETS in China has adopted the rate-based approach for which the study does not find an impact on low-carbon innovation, which can be interpreted as a critical shortcoming for the dynamic efficiency of the national ETS in China, and thus

the manuscript is of high policy relevance. The paper will be of interest to all academics and policy makers working on emissions trading, both in China and beyond. While the analysis appears sound and the article is well written, the communication could benefit from some improvements to more clearly and more directly get across the main findings and implication (see point 1 below). In addition, the embeddedness of the study into multi-disciplinary studies investigating the innovation impact of the EU ETS should be improved (rather than focusing on environmental economics and quantitative studies only) which would also help with more nuanced interpreting and critically reflecting upon the findings (see point 2). Overall, I see great value of the study to be published in Nature Communications.

Shortcoming 1: Clearer communication of findings and implications

- As the difference of a positive innovation impact of mass-based allocation vs no impact for rate-based allocation is a key finding with key policy implications for the national ETS, this needs to be communicated more clearly. First, the terms mass-based and rate-based need to be introduced fairly early on in the article, defining them and summarizing expected differences in innovation impact based on theory and other empirical evidence. Second, in your results and discussion you should explain better the mechanisms behind output-updated allowance allocation and why it is creating an additional subsidy (p. 9 lines 225ff), so that the reader has a clearer understanding of the underlying mechanisms. Finally, and perhaps most importantly, the last sentence of the study/conclusion and abstract shall state much more clearly that based on this study no positive impact on low-carbon innovation can be expected from China's national ETS, and discuss why this is a problem for long-term climate mitigation, leading to a clearer formulated policy recommendation.

- More generally, you rightly discuss the importance of actual ETS design for its innovation impact (following the line of argument of e.g Vollebergh and Kemp & Pontoglio that the innovation impact is more dependent on design features than instrument types). I would recommend that you should take care in defining your design features and other variables which you introduce on p. 7 on line 200 as program composition, design and operation, and to briefly outline which innovation impacts you would expect of these based on the extant literature.

- The wording that the pilot ETS have "significantly induced low-carbon innovation of ETS firms" (in abstract and elsewhere), while technically not wrong, is implicitly making the effect to appear to be grand, while a careful reader will see that indeed it has been quite limited. While the authors later also state that "the overall impact was limited" what sticks with the reader is the impression of a grand effect suggested by the word "significant". To clarify this, the authors should be careful in their wording (to avoid misunderstandings) and should absolutely include the very useful percentage figures they have provided in their analysis in their abstract and conclusion, alluding also there to the additional patents of 4.6%-10.1% (depending on matching method) and to the low share of the low-carbon patents of 1% associated with the ETS. These figures much more clearly demonstrate the actual extent of the innovation impact. The positive impact can be identified through sophisticated matching, but it is really at the moment still very miniscule overall. This needs to come across very clearly in all of the article, also if a reader were just to read the abstract.

- You argue in the conclusion on p. 9 in line 247 that a broader program coverage is needed to increase policy impact, but your earlier findings even more so point to a different allocation approach being needed than the one adopted in the national ETS, so I would mention this alongside program coverage, before you will then, in your last paragraph, unpack the issue of mass-based allocation as driver for low-carbon innovation (not rate-based). Please ensure to state this problem with the national ETS more clearly.

- In light of these comments, consider changing the title of your manuscript so that the reader will already know: limited, but positive innovation impact, but only for mass-based allocation.

Shortcoming 2: Broader multi-disciplinary embeddedness in previous literature on innovation impact of emissions trading schemes

- Not only environmental economists but also innovation and transition scholars have investigated – with various qualitative and quantitative methods – the innovation impact of emission trading systems, perhaps mostly for the EU ETS. However, this literature is not included in the current manuscript, which is a major shortcoming as the totality of studies have enabled a very nuanced and deep understanding of how emission trading systems influence innovation activities. For example, for the EU ETS a recent review on studies investigating its innovation impact by Rogge might be helpful in this regard. There are also a number of studies who have investigated the Chinese ETS pilots in terms of innovation impact as well as the design process of the national scheme based on lessons-learned from the pilots (e.g. Shen, Duan et al). Omitting this broader ETS & innovation literature is problematic – particularly for a multidisciplinary journal as Nature Communications – and leads to shortcomings in the line of argument and in the interpretation of the findings.

- For example, on p. 3 in line 90ff the authors make a generic statement about the superiority of an ETS in terms of inducing innovation – however, this has been heavily debated in the broader literature, so a more broadly informed manuscript would require a more nuanced formulation of this (theoretically) claimed superiority of ETS.

- Another example is the following sentence in lines 93f which makes the important statement that empirical evidence has shown that the actual innovation impact of an ETS depends on a range of factors, but no study is cited for this, nor is this further explained. However, as the study later picks up on the design questions it would be advisable to summarize the state of the art on the empirical findings regarding the evidence for price/stringency, allocation mode (auctioning/types of free allocation). Also, interaction effects with other policy instruments would be worthwhile to report so as to be able to pick up on this in the discussion of your own findings on this matter, but there is no inclusion of references on policy mixes and instrument interactions (e.g. Sorrell, del Rio).

- Another example for this is the argumentation on p.7 on the expected influence of the carbon price on innovation activities of ETS firms (lines 193-197) which only argues with spillovers, while neglecting evidence that has pointed to threshold effects (e.g. 30 Euro EUA prices in the EU ETS) or differences in paying for permits vs receiving revenue from freed ones. You later say that a

higher permit price was not a reason for ETS-induced innovation (lines 206ff) but we read too little about whether this finding can be expected to be generic or whether it is likely arising from (too) low prices, too little differences between the pilots, and a lack/neglect of auctioning in the pilots designs.

Overall, I find your study very interesting from an academic viewpoint and politically highly relevant, and thus my comments are limited to these two main points, and are meant to improve the embeddedness in the wider literature which has investigated the innovation impact of ET schemes, and finetune the clarity of how you communicate your findings and their implications

Response to Reviewer #1

Reviewer #1

Disclosure

As I have informed the Editor, I recently reviewed a version of this manuscript for another journal. I have updated my report to reflect changes in the manuscript, although the authors will recognise many of my comments.

Summary

This paper estimates the effect of China's seven pilot ETS programs on low-carbon innovation, applying the same methods that has been used to study the effect of the EU ETS (Calel and Dechezlepretre, 2016, REStat). The paper also compares estimates across the pilot programs to see what specific design features, if any, are driving the effects they observe. The authors conclude that effect is strongest among programs that use a mass-based emissions cap, and suggest that China's new national carbon market might induce more innovation if it switched from a rate-based to a mass-based cap.

Thanks for the nice summary. We respond to the comments below point by point.

Major comments

1. Engage with the literature. This paper follows similar studies of EU ETS, as well as one recent paper that also examines the impact of China's pilot programs on low-carbon innovation (Cuietal. 2018,AEAP&P). This paper should engage in a more direct conversation with that literature, if only to help the reader understand how it builds on, adds to, and sometimes reaches contradictory conclusions to that literature. For instance, on line 36 the authors claim to "present the first firm-level evidence of policy effects directly of ETS pilots on low-carbon innovation..." but then on line 52 cite two papers (including Cui et al.) that have used firm-level data to provide evidence of the effects on low-carbon innovation. They seem quite dismissive of this previous work, but it's unclear why. As the reader, I want to understand what you're doing differently, and how your findings reinforce or contradict those earlier studies. In particular, Cui et al. find that pilots with a higher carbon price induced a greater innovation response, which you argue hasn't happened. I want to understand why your results are different. Are you perhaps able to replicate their finding, and then show how the result goes away when you take some other factors into account?

We appreciate the opportunity to explain the novelty of our research and make revisions to further highlight our contribution in the manuscript. The reviewer mentions one previous article on a similar topic. Our discussions regarding the article (and in fact another one also on the topic) are intended to show the differences in research design and questions to be addressed, rather than to serve as critiques of the articles.

It has been clearly and reliably shown that emissions trading can induce low-carbon innovation without reducing other technology innovation (Calel and Dechezleprêtre, 2016). Calel and Dechezleprêtre (2016) also provide a nice solution to the issue of count data, common in innovation research using patent as a measure. On that basis, the scientific questions we want to address are (1) whether an ETS has a similar effect in the institutional context without much experience of market instruments (i.e. China) and with other low-carbon policies (the broader policy experimentation of low-carbon pilots including pilot ETS but also regions of other policy experimentations); (2) whether policy spillovers and design features matter to the induced-innovation effect (see responses to comments on spillovers and design features below).

The research questions and associated research design differ our research from Cui et al. (2018). They focus on comparisons before and after the year of *announcing policy experimentation of ETS in 2011* (the actual launch of the individual pilots was in 2013 and 2014) and between *publicly-listed firms in sectors likely to be included in ETS and those in other sectors* (because ETS inclusion criteria were not determined then). This is why we use *policy announcement* to describe the treatment they evaluate, as in "policy announcement in 2011 of ETS pilots has a positive effect on innovation from a small set of publicly listed firms possibly but not necessarily subject to emissions trading[19]."

To help understand Cui et al. (2018), we quote their own description here.

- *Data*: "we have assembled a unique dataset pertaining to the publicly-listed firms in China". They have 1,956 publicly-listed firms in total. The publicly-listed firms are business elite who file a lot more patents than average firms. In comparison, there are 309,656 industrial firms in our sample.
- *Strategy*: "we employ a difference-in-difference-in-differences (DDD) approach". A result of this strategy is that the majority of firms assigned to the treatment group are not actually in any ETS, as explained below.
- *Time difference*: "dummy variable equals 1 for year 2011 and after, and zero otherwise". This was the time that Chinese national government announced that it would experiment with ETS pilots, years before the detailed design of the ETS pilots were finalized.
- *Sector difference*: "being 1 if sector j is subject to the regulation in any pilot regions, and zero otherwise", and because of this "Shenzhen regional pilot is excluded in this study, because all sectors in the manufacturing industry are subject to carbon ETS in this pilot." This explains why the majority of firms in the treatment group are not actually in any ETS – programs vary substantially in sectoral coverage, and the *union* of individual program coverage deterimines this variable.
- In addition, it is not quite accurate to state "Shenzhen regional pilot is excluded in this study, because all sectors in the manufacturing industry are subject to carbon

ETS in this pilot." The statement is only correct when using two-digit industry classification. If a finer four-digit classification were used, one would find that half of the industries are not included, so that Shenzhen ETS should not necessarily be dropped out of their research.

- *Regional difference*: "equaling 1 if region r is a carbon market pilot, and zero otherwise". The potential confoundedness from the broader policy experimentation of low-carbon pilots, which includes ETS pilots and takes a lot of our efforts to address, is not considered.

As one may have seen, a main challenge to any inference of a direct ETS effect based on the estimation from Cui et al. (2018) is the assignment of treatment status, let alone time difference used, estimation method, or other aspects in design. The treatment status is assigned to all the publicly-listed firms in all sectors included in the *union* of sectoral coverage from six ETS programs, excluding Shenzhen. As a result, the majority of firms assigned to the treatment group are actually not in the ETS – they are either in an ETS sector but not included, or not even in the ETS sector, but the sector is covered by another ETS. For example, *no* textile firms are included by the Guangdong ETS or Tianjin ETS, but because some textile firms are included in the Shanghai ETS, *all* textile firms in Guangdong and Tianjin are assigned to the treatment group in Cui et al. (2018).

Addtionally, because of the design, firms are compared to those in other sectors, which may have a totally different trend of innovation. In the extreme case if Shenzhen ETS were not excluded from the analysis, all the manufacturing sectors in all seven regions would have been assigned to the treatment group. One reason for this strategy is probably that it takes time to get firms' actual treatment status – we have to get a list of firms for each ETS in each year individually and sometimes file information disclosure request to the government. Again, it is more accurately to describe the policy evaluation practice of Cui et al. (2018) as about *policy announcement*.

The estimation of Feng et al. (2017) focuses on firms in the Hubei ETS only, as an extension of their synthetic control estimation also for the Hubei ETS. From the limited information we get, their treatment assignment seems accurate on the ETS firms in Hubei. They focus on innovation in general instead of specific technology areas (i.e. low-carbon innovation), and also compare before and after 2011 and use a dataset of listed firms. According to our Fig. S4 and S5, Feng et al. (2017) would not have a very representative sample of patents, due to the timing of their research and patent publication. This issue may or may not bother Cui et al. (2018) too, depending on their date of data collection, of which we are not aware.

In comparison, we focus on the effect after the actual launch of ETS, assign firms to the treatment group according to actual enrollment in the ETS, and compare them with

similar firms in the same sector and of similar characteristics via matching. We recognize the importance of policy announcement in October 2011 and therefore tested alternative baseline years in matching, as shown by Table S11 in Section 5.4 "Alternative Samples and Baselines to Test Unobservable Selection Bias". The result remains almost the same, i.e. 1.75 (1, 2.9).

Replication of Cui et al. 2018 is an interesting idea. But as explained above, our research differs from theirs in all dimensions of research design (specially time and treatment assignment), data (1,956 publicly-listed firms in theirs vs. 309,656 industrial firms in ours), timing of patent data collection, addressing other confounding factors, and questions can be answered (policy announcement vs actual implementation). Explain the differences in results seems neither technically straightforward nor of scholarly value.

Having said all these, the authors agree that some revision can be made. As the reviewer mentioned above, previously the authors wrote in line 36 "Here we present the first firm-level evidence of policy effects directly from emissions trading and differential program designs in China." The authors intended to use "first" and "directly" to stress the differences from the literature, following previous feedbacks received. It is not the authors' intention to make a territorial claim. So now this sentence reads "Here we present firm-level evidence of policy effects directly from emissions trading and differential program designs in China since 2013." A similar statement in the abstract has also been revised by removing "the first".

2. Explain the source of variation in treatment. A matched difference-in-differences design can reveal a causal effect only if there is a source of variation in treatment that, for some subset of companies, is credibly uncorrelated with unobserved drivers of future innovation outcomes. As currently written, the paper does not explain to the reader why some companies are regulated under a pilot program, while other fundamentally similar companies are not. Without a persuasive explanation I will tend to presume that the unregulated companies are not in fact similar. To the extent that you're matching on observable characteristics, you would only ensure greater dissimilarity on unobservable characteristics.

This reviewer comment is about research design, which applies a matching method to observational data so that the treatment assignment can be interpreted as a randomized experiment in the matched sample. More formally, the matching method recovers the assumption of unconfoundedness, i.e. the treatment assignment is free of dependence of potential outcomes after matching. The unconfoundedness assumption is not testable in principal. So the reviewer suggests that the authors should state more explicitly the rationale of using matching methods *ex ante* in the research design phase. We respond to this request here in the paragraphs below and revise our statement in the main article.

In our response to the reviewer's next comment #3, we explain how we assess the plausibility of the untestable assumption of unconfoundedness indirectly, and provide evidence that supports the assumption in Tables R3 and R4.

There is a paragraph on this in the first section of the SI where you explain that "Two firms may be assigned to alternative statuses, one in an ETS program and the other outside, because of their differences in location, combination of sector and location, combination of location and emission, and the year in which they reached the emission threshold." As best I can parse this statement, there are three different identifying margins: (1) some matches will be identical in every respect except their pre-treatment location, (2) some matches will be identical in every respect except their pre-treatment sector, (3) some matches will be identical in every respect except their pre-treatment emissions. Is this right? Each one tells a qualitatively different story about identification, but the paper doesn't make clear to what extent you are relying on each of these margins. Then in Table S3, it seems you're actually matching exactly on 4-digit industry sector and pilot region, which seems to leave only the pre-treatment emissions margin. So in reality, you seem to be systematically comparing similar heavy polluters with lighter polluters, and implicitly asserting that heavy polluters are no more likely to innovate in the future, conditional on the set of covariates. Please state this simply and clearly. There is no reason to make it difficult for the reader to figure this out (if this is indeed right?). It is essential that you clearly explain this in the main paper, too, not just in the SI.

To follow up on the reviewer's summary of identifying margins, the matching method explores both (1) and (3). But both margins need additional clarification, as neither of them necessitates a comparison between heavy and light carbon emitters.

For the first margin. In our main estimation, we require the matched pairs to be in the low-carbon pilot regions and the same 4-digit sector. The low-carbon pilot regions are the provinces and cities selected by the NDRC to experiment with all kinds of low-carbon policies, including the two provinces and five cities with ETS pilots (ETS region hereafter). In other words, the ETS regions is a proper subset of the low-carbon pilot regions. The low-carbon pilot regions are operationalized by a dummy variable indicating whether a firm is in any of the regions or not.

So the first margin suggests that an ETS firm can be matched with an identical non-ETS firm because a) the latter is in a low-carbon pilot region but not a ETS region; b) the latter is in the province/city with a different ETS pilot where its sector is not included (e.g. a glassmaker in Shenzhen ETS matched with a glassmaker in Guangdong, where glass industry is not included in the Guangdong ETS); or c) the latter is in the province/city with a different ETS pilot where its sector is included but the inclusion threshold of emission is higher (e.g. a still mill in Shenzhen ETS of 5,000 ton emissions

matched with a steel mill in Guangdong, where only steel mills above 20,000 ton emissions are included in the ETS). In our robustness checks, we explore a stricter exact match condition of ETS regions, where we use b) and c) but not a).

Exploring the first margin inside the low-carbon pilot regions connects to the scientific question we want to address – whether an ETS still have an induced innovation effect with presence of other low-carbon policies. The low-carbon pilot regions consist of a lot of provinces and cities, each of which experiments with its own low-carbon policy. By matching ETS firms with those identical non-ETS firms in low-carbon pilot regions, we estimate the additional effect of ETS on innovation on top of any effect driven by other low-carbon policies in China.

For the third margin. Strictly speaking, we use the emissions in the base years, not the pretreatment emission immediately before the actual treatment. The base years are the period during which a firm's emissions is measured to determine whether it is included in an ETS. Note that as shown in Table S1, the base years span from a few years (2008/2009) to one or two years (2011/2012) before the launch of ETS pilots. A firm ever reached the emission threshold in a base year once (e.g. only in 2008 and never after) would be included. In comparison, a firm exceeded the emission threshold in 2012 or 2013 but not in a base year is not included. We are not saying that this identification is as strong as the previous one, but ETS and matched non-ETS firms in the same province/city are not of seriously systematic difference in their emission level right before the laucn of ETS.

Here we can map the geographic distribution of the matched firm pairs. The main estimation is based on 852 matched firm pairs. Only 58 ETS firms or 6.8% are matched with non-ETS firms in the same city or province, i.e. from the third margin. 262 ETS firms or 30.8% are matched with firms in a different ETS region. 532 ETS firms or 62.4% are matched with firms in the non-ETS low-carbon pilot regions. The latter two are based on the first margin.

To confirm the robustness of our strategy, here we further estimate and present in the response letter the treatment effect using the subsample of ETS firms matched with non-ETS firms strictly in a different location. This leads to 794 matched pairs. The point estimate and upper bound for the treatment effect remain 1.75 and 1.9 patents, while the lower bound increases to 1 patent.

In the main article, explanations for the identification strategy is added to the method section. Now we have a separate paragraph to explain:

"Two sets of marginal differences between ETS and non-ETS firms were explored for a quasi-experimental design via matching: two firms may be identical except their

locations, so that one is in the ETS and the other is in place of no ETS but other climate policies, an ETS with a narrower sectoral coverage, or an ETS with a higher emission threshold for firm inclusion; two firms may be identical except their highest emission levels during the base years, which does not necessarily indicate a difference in pre-treatment emissions."

The source of identifying variation is then somewhat different when you are estimating spillovers. In this case, 'treatment status' depends on both the ETS/non-ETS quasi-randomisation (discussed above) AND on the properties of the networks. If some firms are more prolific co-patenters, for instance, they are more likely than average to be assigned to 'treatment' when a random firm is assigned to the ETS. These network properties alter the permutation probabilities and the standard errors.

The reviewer raises an issue about the identification strategy for spillover effects, suggesting that co-patenters, because of the nature of their collaboration, may be more likely assigned to the treatment group. While most of our investigation of spillovers concerns policy spillovers, co-patenting is about knowledge spillovers following Calel and Dechezleprêtre (2016). This argument may extend to other types of spillovers, which are addressed in our response to reviewer #2. Here we focus on the issue of co-patenting.

To address the concern, two additional efforts are made. The first is a direct comparison between the co-patenters of ETS firms and the matched control firms, to see whether they are different in important aspects that may affect their patenting activities. Table R1 shows that the co-patenters were indeed different from the matched control firms in their size and previous co-patenting experience. The second is to add previous co-patenting as a variable in matching. This may not fully address the issue with regard to the structure of collaboration networks, but can at least mitigate it. Table R2 shows that by including previous co-patenting in matching, the result remains nonsignificant with a smaller point estimate of zero.

One note should be made here for clarification. Because Chinese firms rarely collaborate in patent filing, the result about co-patenting is based on an extremely small sample. A reasonable caliper of 1.5 cannot even be applied to the matching process, which would further reduce the matched sample to only 13 pairs of firms. So there is no conclusive statement made in the article about co-patenting.

Finally, one paragraph has been added to the Methods section to reflect that the estimation strategy applied to spillovers would not be as strong as applied to main estimation. It reads:

"A same estimation strategy was used to test spillover effects. It matched non-ETS firms potentially subject to spillovers – reporting firms, large firms in ETS sectors or in

Shenzhen ETS sectors, co-patenters of ETS firms – with other non-ETS firms not subject to these influences. Unlike clear-cut inclusion criteria, however, spillovers take networked channels and can be ambiguous. Therefore, the same identification strategy may not be as strong as in the main estimation.”

Table R1: *t* tests for key variables of co-patenters and matched control firms.

	difference in the means	standard error	p-value
2011-12 low-carbon patenting	0.6315	1.250	0.614
2011-12 total patenting	35.118	26.681	0.190
historic low-carbon patenting by 2012	3.132	4.346	0.472
historic total patenting by 2012	93.355	82.802	0.261
2012 number of employee	1177	485	0.016
2012 total asset (million RMB)	1714	671	0.012
firm age by 2013	0.697	1.808	0.700
2012 output (million RMB)	2075	814	0.012
historical co-patenting experience	2.18	0.35	0.000
four-digit industry sector	exactly matched		
low-carbon pilot region	exactly matched		

Table R2: Spillovers to co-patenters of ETS firms

	point estimate	95% confidence interval	matched firm pair
co-patenters of ETS firms (previous result without matching previous co-patenting)	0.75	(-1, 3.9)	79
co-patenters of ETS firms (adding number of previous co-patenting in matching)	0	(-1.9, 1.9)	76

The source of identifying variation is again different when you’re analysing effect heterogeneity across programs. In that case you’re not comparing an ETS-firm with a non-ETS firm that was just below the emissions threshold. Either you are matching ETS-firms to non-ETS firms from other pilot programs, or perhaps you are not matching at all? Either way, you seem to be using regional differences in design rather than within-region thresholds. This has two consequences. First, it’s harder to think about the regional variation in design as plausibly randomised. Second, even if it is quasi-random, you have to think harder about how you permute treatments to get your standard errors. If you’re imagining swapping out the ‘Beijing’-treatment for the ‘Shanghai’-treatment for one Beijing-based firm, can you do that without simultaneously doing the same swap for all Beijing-based firms? Block randomisation of this sort would radically reduce the number of permissible permutations of the treatment vector and inflate your standard errors. I think some version of this exercise

is interesting, and absolutely essential to this paper, but the authors should be careful to signal that the claims about causal identification are quite different and a bit weaker here.

If we understand correctly, this comment raises two related issues. First, because of the test of heterogenenous treatment effects, heteroscedasticity needs to be explicitly addressed, and our strategy using clustered standard errors may not be the perfect solution. The reviewer suggests that we randomize the assignment of ETS design/operational features among the seven pilots to solve the issue. Second, a clean identification strategy is needed to support a causal claim of any effect from a design/operational feature of ETS. Block randomization may do part of the job but may not be as strong as the main estimation. So if there is no better strategy, we have to state this in identifying heterogeneous treatment effects.

The issues are addressed by three strategies in light of block randomization, with the intention to provide a more reliable estimation of whether ETS features matter to the outcome. Before laying out our plan, we need to explain that while some features vary across ETS programs (e.g. average market price, whether there is auction), others also vary within a program (e.g. allowance allocation is different for different sectors and firms in an ETS). The alternative permutation strategies are designed according to the fact. Our focus is the effect of mass allocation, a main finding of the article.

The first strategy assumes that all the design and operational features of an ETS nest together and are not separable. In other words, an ETS pilot program, with reference to the firm profiles, made decisions of sectoral coverage, emission levels for inclusion, allocation methods, and so on, which collectively affected the price of the market. Block randomization may not be a preferred strategy in this case, because it separates some features from others anyway. One thing can be done is to randomize the assignment of mass vs rate-based allocation and keep other features unchanged to see whether the significant result is simply produced by chance or associated with other features. If it were the case, the significant result from mass-based allocation would be likely reproduced. In operationalization, the probability for an ETS firm to get mass-based allocation corresponds to the percentage of mass-based allocation in total. Following the regression in Table 2, coefficients are recorded. The process is repeated for 500 times to get a distribution. Fig. R1 shows that if firms were randomly assigned to an allocation method, the significant induced-innovation effect from mass-based allocation would diminish. The significant result from mass-based allocation is not likely produced by chance or other features.

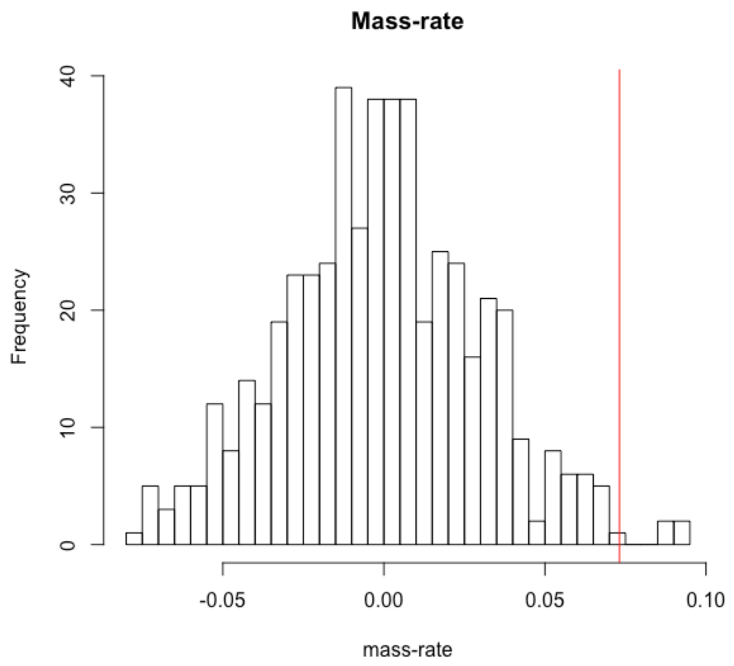

Fig. R1: Distribution of estimated coefficients of mass allocation based on 500 placebo tests. The red line shows the actual coefficient in Table 2.

Fig. R2 shows the result for price. The distribution is similar to that of Fig R1. Because price has no effect according to Table 2, the result is simply a confirmation without providing any additional insight.

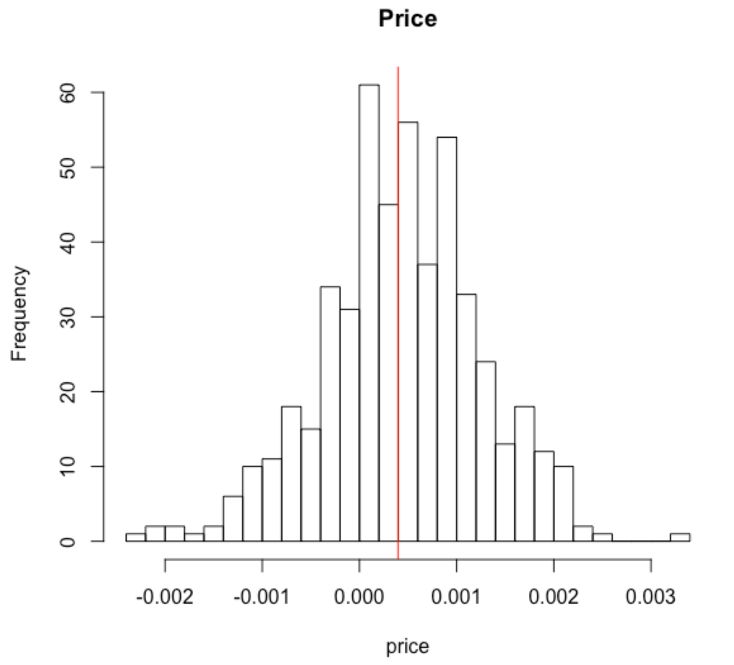

Fig. R2: Distribution of estimated coefficients of price based on 500 placebo tests. The red line shows the actual coefficient in Table 2.

The second strategy assumes that design and operational features of an ETS are separable. This is a stronger assumption than one used by the first strategy, but has its advantage: we can focus on the main features that may affect the outcome and investigate their individual effects more explicitly. Particularly, we focus on mass versus rate-based allocation.

In operationalization, the mass/rate-based allocation maintains unchanged while price – another important feature – is randomly permuted. Because average price varies at the program level, block randomization, which cannont be used for the first strategy, can be used here to get a full set of permutation as suggested by the reviewer. This means all the firms in one ETS would be assigned with the price of another ETS, and the total number permutation is 5040 (7P7). The rest is similar to that of the previous strategy – cofficients are recorded each time based on the same regression. Fig. R3 shows that the actual estimate is very close to the median of the permuted distribution, which rarely has any estimate close to or below zero. Fig. R4 confirms that the coefficient of price is close to that of the median of the randomly permuted distribution near zero.

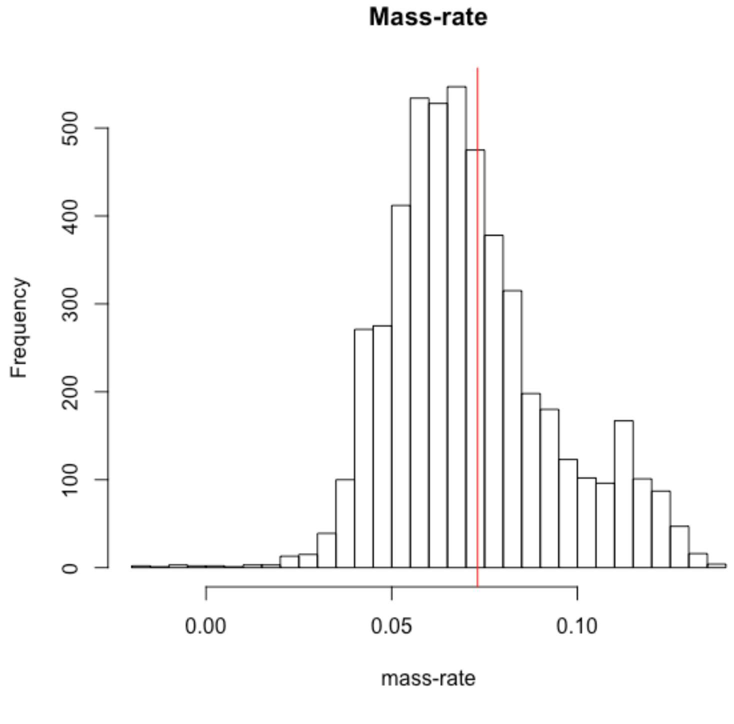

Fig R3: Distribution of estimated coefficients of mass allocation based on 5040 times of block randomization for price. The red line shows the actual coefficient in Table 2.

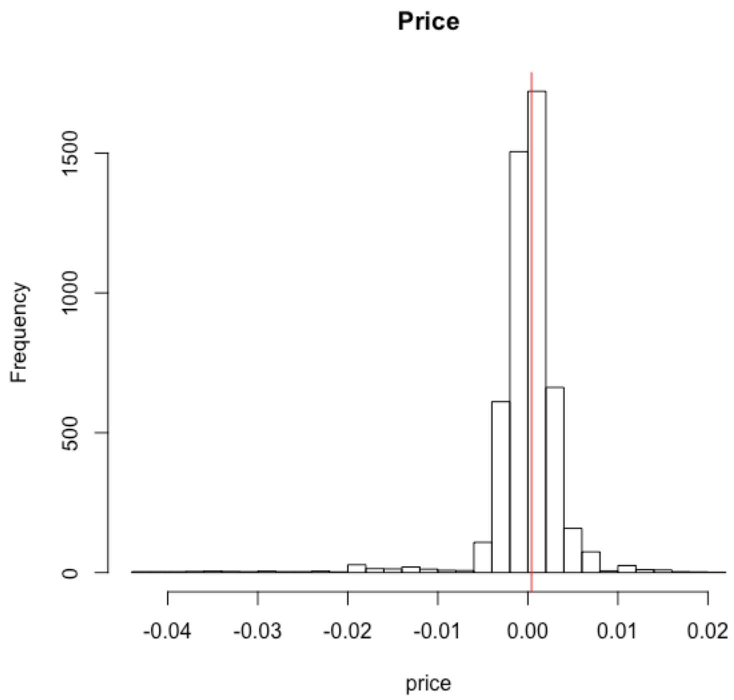

Fig R4: Distribution of estimated coefficients of price based on 5040 times of block randomization for price. The red line shows the actual coefficient in Table 2.

The third strategy relaxes the assumption from the previous one by assuming that all the program features are nested except mass/rate-based allocation. This is a weaker and practically valuable assumption that helps to further confirm the effect from mass-based allocation. We maintain mass/rate-based allocation unchanged and permute all the other features together via block randomization, because all the other features vary at the program level except mass/rate-based allocaiton. All the firms in one ETS would be assigned with all the other features of another ETS except allocation method, and the total number of permutation is 5040 (7P7). The rest is similar.

Fig. R5 shows that while the whole distribution shifts to the left, it still is significantly larger than zero, with an empirical *p* value of 0.024 (only 121 out of 5040 times at or below zero). This suggests that while the estimated effect of mass-based allocation may be partially associated with other program features, mass-based allocation is still the most important factor that explains ETS-induced innovation. Mass-based allocation alone explains the policy effect regardless of any randomized permutation. Fig. R6 further confirms.

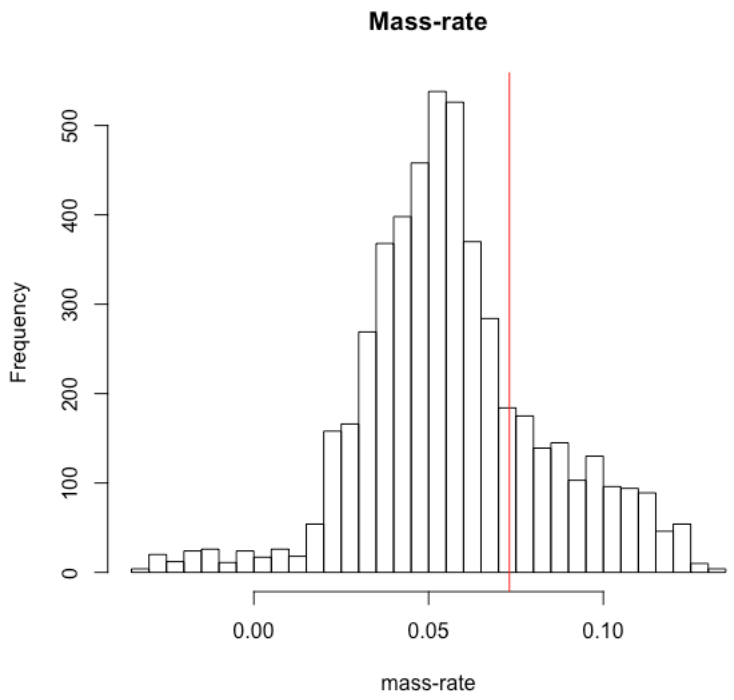

Fig R5: Distribution of estimated coefficients of mass allocation based on 5040 times of block randomization for all other features. The red line shows the actual coefficient in Table 2.

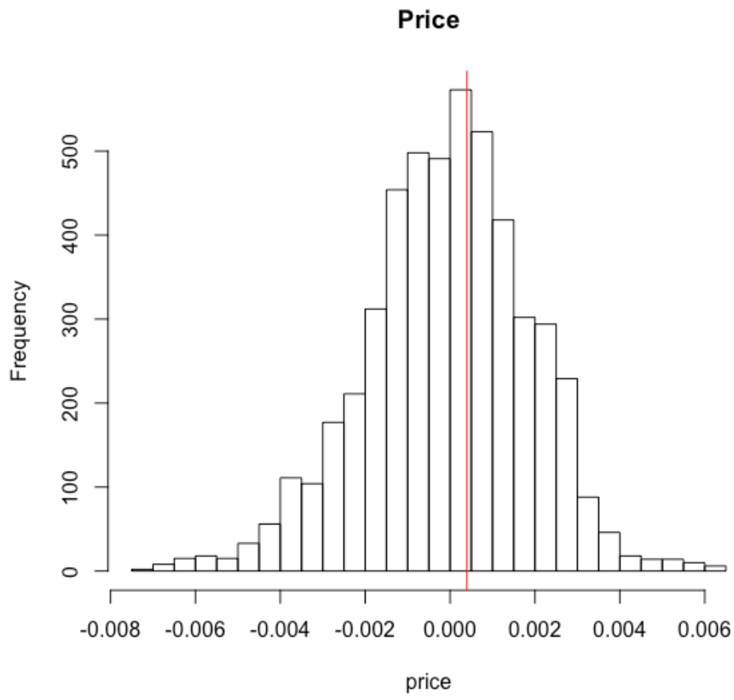

Fig R6: Distribution of estimated coefficients of price based on 5040 times of block randomization for all other features except mass allocation. The red line shows the actual coefficient in Table 2.

A final note is that we take the suggestion from the reviewer to highlight that the identification strategy for heterogeneous effects from program features is weaker than that of the main estimation. A paragraph has been added to the Methods section. It reads:

"It has to be noted that, although the same matched sample from the main estimation is used, it does not represent the same identification strategy. The main estimation takes advantage of different program inclusion criteria to use matching to recover randomized assignment of ETS status for causal identification. But when making inference for the effect from different program features, assignment of these features is not likely made randomized through matching, because they may be correlated with program inclusion criteria. We therefore discuss confounding factors and check the robustness of the results."

3. Changing the match. The matching methodology and the resulting matches appear to have changed since the original submission. They authors seem to have changed the set of covariates substantially: adding total assets, employment, and some quadratic terms, applying log transformations, matching exactly for pilot region, and dropping output. I would like to see some kind of discussion for why they thought it was important to match on some things and not on other things. Remember that you're matching to balance potential outcomes, so what is the evidence and argument that these particular covariates are good predictors of potential outcomes in the absence of an ETS?

This comment is about matching specifications. Particularly, the reviewer is interested in the reasons behind the selection of covariates used in matching. To address this comment, we need to go back to the discussion about research design based on matching. In our responses to the previous comment, we explain that matching methods are appropriate when they can recover the conditional unconfoundedness assumption. The assumption is not testable directly, but its plausibility can be assessed. Such an assessment is the key to the selection of covariates in matching.

To assess the plausibility of unconfoundedness, Imbens (2015) lays out two strategies: 1) to estimate a treatment effect on a variable known a priori not to be affected by the treatment, typically pretreatment outcomes and covariates; 2) to estimate a pseudo-treatment effect, known a priori not to have an outcome. The latter is done by our placebo tests shown by Table S13 and Fig. S9. The former connects to the selection of covariates. It can be operationalized by testing the balance of all the covariates and lagged outcomes between treatment and matched control observations, while using only a subset of the variables in matching.

Therefore, as the reviewer suggests, we need prior knowledge to guide our selection of matching variables of predictive power and parsimony. We first prioritize 4-digit industry code and location in the low-carbon pilot regions as two exact match

conditions. Four-digit industry code is the finest industrial sector in China. It is associated with ETS inclusion and tends to capture a few important unobservables such as production process, technology, input, and product. Low-carbon pilot regions are the provinces and cities approved by the National Development and Reform Commission to experiment all kinds of low-carbon policies, including seven ETS pilots. It is important for eliminating any effect from simply announcing a low-carbon policy and for addressing our research question of the ETS-induced innovation effect on top of other low-carbon policies. Similar exact match conditions are also used by Fowlie et al. (2012) in evaluation of the NO_x trading program.

For the continuous variables, we first consider firms' historical innovation capabilities in general and specifically for low-carbon technologies, as well as their innovation capabilities in recent years. Firms' historical and recent innovation capabilities, measured by patent counts, are likely associated with firms' future innovation. The quadratic forms are useful to emphasize the role of pretreatment innovation as the most important predictor of post-treatment outcome, while there are other predictors, as in Calel and Dechezleprêtre (2016). Innovation activites are also affected by firms' financing capabilities, where firms' financing constraint is commonly measured by SA index. The SA index is calculated based on firm age and size, which is measured by market capitalization (i.e. total asset as a proxy). Aside from market capitalization, we need another measurement of firm size as a proxy for unobservable emissions, conditional on 4-digit sector. While both number of employees and monetary output seem appropriate, we prefer to use only one for parsimony and for avoding high correlation between asset, output, and number of employees. The final selection of the number of employees because it is not as highly correlated with total asset as output. The number of employees has also been used by the NO_x trading evaluation (Fowlie et al., 2012).

The tables below show that most of the pretreatment variables are nicely balanced, although only a subset of the variables are used in matching. In our submission, we only test and present one variable not used in matching, i.e. output. Here we consider three remaining variables in our dataset that have not been used in our analysis before: profit, sales, and wages. We find that their values are also nicely balanced. In addition, we construct the outcome variables of low-carbon and total patenting in 2013 (i.e. when ETS pilots already lauched) here. Because innovation takes time, these immediate posttreatment outcome variables are still balanced, with the only exception of low-carbon patenting in 2013 (the year when five ETS pilots launched) under equivalence test. The exception is the same as in Calel and Dechezleprêtre (2016), who explain the issue as a result of only a small set of firms filing low-carbon patents. This also explains our case, where firms are more likely not to file any patent in a single year of 2013 than in two years of 2011-12, which are balanced. These balancing tests provide stronge evidence that the unconfoundedness assumption is very plausible, which in turn

supports our selection of covariates in matching.

Table R3: *t* tests for all variables after matching.

	difference in the means	standard error	p-value
2011-12 low-carbon patenting	0.0012	0.018	0.949
2011-12 total patenting	0.088	0.809	0.913
historic low-carbon patenting by 2012	-0.0023	0.025	0.926
historic patenting by 2012	0.175	1.703	0.918
2012 number of employee	77.5	71.8	0.280
firm age by 2013	0.331	0.402	0.410
2012 total asset (million RMB)	102	133	0.442
2012 output (million RMB)	75.6	250	0.762
**2012 profit (million RMB)**	-3.76	17.1	0.826
**2012 sales (million RMB)**	146	252	0.561
**2012 wages (million RMB)**	3.91	5.16	0.449
**2013 low-carbon patenting**	0.007	0.025	0.777
**2013 total patenting**	0.352	0.401	0.381
four-digit industry sector	exactly matched		
low-carbon pilot region	exactly matched		

Notes: Variables in red are not used in matching. Variables in bold are newly tested.

Table R4: Equivalence tests for all variables after matching.

	median difference	critical equivalence range	equivalence range
2011-12 low-carbon patenting	0	±<0.01	±0.075
2011-12 total patenting	0	±<0.01	±3.34
historic low-carbon patenting by 2012	0	±<0.01	±0.105
historic patenting by 2012	0	±<0.01	±7.02
2012 number of employee	7	±25	±296
firm age by 2013	0	±0.49	±1.66
2012 total asset (million RMB)	10.2	±29.7	±550
2012 output (million RMB)	40.3	± 53.0	±1031
**2012 profit (million RMB)**	-1.09	± 15.8	±70.7
**2012 sales (million RMB)**	21.1	±73.7	±1039
**2012 wage (million RMB)**	1.97	±4.32	±21.3
**2013 low-carbon patenting**	0	±<1.99	±0.103
**2013 total patenting**	0	±<0.99	±1.66
four-digit industry sector	exactly matched		
low-carbon pilot region	exactly matched		

Notes: Variables in red are not used in matching. Variables in bold are newly tested.

Setting these crucial clarifications aside, an even bigger concern is that the set of

matched companies is now completely different from the original submission. You are now choosing how and who to match after having peaked at their outcomes. This lays you open to the risk that your matches are either consciously and unconsciously influenced by the estimates they produce. I think it's really important that your main analysis be based on the original method and sample. You should definitely include analysis based on this alternative matching method, but it should be included as one of your robustness checks. The distinction is important. What I ask, and asked before, is that you report the imbalances on all covariates, whether or not they were inputs into the matching algorithm. But the authors seem to have taken the opportunity to match on these extra variables in order, presumably, to reduce imbalances for the original matched sample that weren't initially reported. The results are ultimately not wildly different, so I don't understand the reluctance to report the original estimates.

Some background information is needed in response to the comment. By "original submission", the reviewer refers to a submission to another journal that has been rejected. The "original submission" uses the same identification strategy but not exactly the same set of matching covariates as that have been used in this submission, which is in fact an original, new submission itself. This submission differs from the other one in its original form in questions to be addressed and results presented. The change benefits from the previous review process of the other submission, but they are two independent submissions.

There are two important reasons for the matching specification used in this submission to differ from the one used in the other submission. The first is an exact match on location within the low-carbon pilot regions, which was suggested by a reviewer of the other submission. With this condition, we are comparing an ETS firm with a firm exposed to some low-carbon policy influences, which directly connects to our research question. Without it, the control may or may not be exposed to the influences, which is ambiguous. So the exact match condition has to be included.

The second is total asset, which was reminded by another reviewer of the other submission too. Total asset is a necessary component of SA index, which predicts firms' financing constraint and therefore affects innovation. It is a particularly important predictor of credit constraint and innovation of Chinese firms, as the authors realized in another research project they have been working on. So we prefer to include it. All the other choices have been explained by our previous responses. Switching from output to the number of employees is a reasonable choice as the former is more correlated with total asset (0.82) than is the latter (0.62), so that using the number of employees provides additional information.

We do not understand why the reviewer suggests that the outcome may be affected by the change of matching specifications. The current specification, as we have explained,

is mainly determined by speculations a priori, thanks to the previous review process. Outcome variables have nothing to do in this design process. The robustness checks in the supplementary information also show that many specifications produce statistically more significant estimations than our baeline estimation. For example, in Table S8, eight out of eleven alternative spefications have a larger lower bound than the one in the baseline estimation.

We would understande if by “outcomes”, the reviewer referred to the results of balance tests. The result of balance tests was a nice reference in the choice between the number of employees and output. But there is a more important reason a priori that dominates this selection, i.e. correlations of the alternative varables with total asset. Selections of most other variables are determined by prior knowledge, although they bring better results of balance tests at the same time. In addition, the authors did not even realize that the specification also performed very well in the balance tests of pretreatment revenue, sales, wage, as well as 2013 patents. The newly tested variables suggest the plausibility of unconfoundedness assumption.

To summarize, the authors would like to emphasize again two reasons for current specification. First, this is a new submission regarding a different (though similar) research question (ETS effect on top of other climate policies). The specification reflects our understanding of the selection of matching variables at the current stage. The specification used by a previous submission that the reviewer reviewed before is not applicable to the research question in this submission, at least because an exact match of the low-carbon pilot region is necessary.

Second, we understand that the reviewer may have a concern that over-emphasis on the balance of the observables may compromise the balance of the unobservables and therefore the unconfoundedness assumption. The balance tests of previously not included variables in Tables R3 and R4 suggest that it is not an issue for the current specification.

4. Relevance of unmatched ETS companies. As the authors say, 40% of ETS firms are set aside because they couldn’t find suitable matches, and these 40% are typically more innovative. My concern is that if the effect is substantially different in this group of companies, it completely changes the quantitative and qualitative conclusions of the paper. Even a small negative effect might be enough to cancel out the effect you observe in the matched sample, for instance.

I appreciate that you’ve done a bit in the SI to look at the relevance of the many unmatched ETS firms. It would be helpful to also include some kind of bounding exercise, since it would provide a cleaner answer to the question of what kind of influence these omitted ETS firms could theoretically have on your estimates without

having to compare them to a bunch of unsuitable controls. I think it's important that the results of this kind of bounding analysis are reported in the main results section of the paper, since it gives the reader context for interpreting the level of uncertainty in your conclusions beyond the statistical significance. You need to be more up front about the fact that you're looking at an unrepresentative subset of about 60% of ETS firms, and in particular, how sensitive your overall conclusions are to what effect the pilots might have had on the other 40%.

There are two issues related to this comment. The first is whether the matched sample is well representative of the general population, so that the findings are generalizable to a broader context, e.g. a national ETS in China. It is reasonable to suspect the representativeness of the unmatched firms, not the matched ones, because otherwise the unmatched firms would have been matched and included in the analysis. The second is whether the innovation effect of the pilot ETS would be changed when considering the unmatched firms. Bounding exercise helps to sovle the second issue, not the first one, which is probably more important. The two issues are addressed below separately.

1) Representativenes of the matched ETS firms

The representativeness of the matched sample is evaluated following two methods as laid out by Caliendo and Kopeinig (2008). First, we calculate the perceptage of the general population (i.e. the whole sample after merging firm and patent datasets) that falls in the range of patenting level of the matched ETS firms (larger than minimum and smaller than maximum). Table R5 shows that the range of patenting levels as determined by matched ETS firms covers more thatn 97% of industrial firms in the whole sample.

Table R5: Minima and maxima comparison of representativeness.

	pre-treatment low-carbon patenting	pre-treatment total patenting	historic patenting
in ETS region	97.5%	99.9%	100%
in low-carbon pilot region	99.6%	100%	100%
in mainland China	99.7%	100%	100%

Second, we constitute bins, each based on a patenting value of the matched ETS firms, and calculate the percentage of firms falling in these bins from the whole sample. This strategy addresses the concern that some outliers in the matched ETS firms may drive up the range of patenting levels, and improve the coverage of Table R5. Table R6 shows that the matched ETS firms still account for the patenting activities of more than 85% firms in the ETS region and are even more representative at the national level, covering more than 95% of the firms.

Table R6: Comparison of representativeness after trimming.

	pre-treatment low-carbon patenting	pre-treatment total patenting	historic patenting
in ETS region	94.9%	90.2%	86.2%
in low-carbon pilot region	98.6%	96.6%	95.3%
in mainland China	98.9%	97.3%	96.4%

Considering the result of Table R6, the actual challenge to generalizability is not from excluding the unmatched firms, but rather from some outliers remaining in the matched sample. Because the ETS region includes three cities that are most innovative in China, i.e. Beijing, Shanghai, and Shenzhen, a small number of innovation giants, if not dropped out of the sample, may affect the estimation and diminish its generalizability.

To tackle this issue, we remove the outliers with respect to innovation activities from the matched ETS firm sample and redo the estimation. The outliers are defined as firms with pre-treatment (2011-2012) or historically accumulated low-carbon patents or total patents beyond the 99 percentile of the whole sample. In patenting analysis, because of the large number of zero patent, 99% rather than 95% is usually used to define outliers. According to the definition, three outliers are removed from the original observations. The point estimate remains 1.75, with the 95% confindence interveal (1, 1.9), i.e. a higher lower bound.

2) Considering the unmatched firms and their implications to the result

We report three efforts here to incorporate unmatched firms in our analysis. First, the representativeness of the unmatched firms are evaluated. Second, with relaxed matching strategies, the unmatched firms are included in the sample to estimate the effect. Third, the influences from unmatched firms to the estimation are evaluated via bounding.

Table R7 shows the first result by simply removing the outliers as defined above. It can be seen that there are a large proportion of outliers (around a quarter) among the unmatched firms, while only three outliers among the matched firms. After removing the outliers, the matched and unmatched firms become similar, but the unmatched firms are still larger and older. Tables R6 and R7 collectively suggest that the matching process are actually removing the unrepresentative firms, rather than maintaining them.

Second, we tried to get the unmatched firms matched to the largest extent through adjusting to a larger caliper or using alternative matching algorithm (i.e. propensity score matching). By increasing caliper to 2, we can increase the ETS firm matched from 852 to 969. The point estimate becomes 1 with (0, 1.9) the 95% confidence interval. As indicated in SI section 5.7, we could match more than two thirds of the unmatched firms

with non-ETS firms based on the same exact match condition without any caliper, yet the matching quality is too low for reliable inference. Based on propensity score matching, section 5.8 of the SI matches 1325 out of 1454 firms, or 91% of the total. The point estimate becomes 0.75 with (0, 1.9) the 95% confidence interval. Although the matching quality is compromised, the result is not qualitatively changed.

Table R7: Summary statistics of matched and unmatched firms after removing outliers.

	main specification (caliper=1.5)		removed outliers	
	matched	unmatched	matched	unmatched
2011-12 low-carbon patenting	0.03	4.33	0.01	0.22
	(0.39)	(28.21)	(0.13)	(0.60)
2011-12 total patenting	1.46	88.71	0.80	5.03
	(17.39)	(596.41)	(3.04)	(8.09)
historic low-carbon patenting by 2012	0.05	13.82	0.02	0.58
	(0.55)	(94.75)	(0.17)	(1.19)
historic patenting by 2012	2.83	300.43	1.50	12.78
	(36.43)	(2,426.17)	(4.65)	(16.55)
2012 number of employee	870	2,880	868	1,942
	(1,672)	(7,897)	(1,674)	(3,844)
2012 total asset (million RMB)	1,174	4,743	1,174	2,589
	(2,859)	(17,953)	(2,864)	(6,931)
firm age by 2013	13.17	17.88	13.17	17.84
	(8.40)	(12.90)	(8.41)	(12.76)
2012 output (million RMB)	1,313	4,567	1,315	3,070
	(4,801)	(16,198)	(4,809)	(10,200)
2014-15 low-carbon patenting	0.14	4.86	0.14	0.33
	(0.91)	(27.09)	(0.90)	(1.47)
2014-15 total patenting	2.53	93.99	2.28	7.03
	(11.58)	(577.38)	(10.36)	(19.54)
observations	852	602	849	462

Third, some bounding analysis has been performed as suggested. Because the main concern from the reviewer is that the unmatched ETS firms would not be as responsive as the matched ones, we focus on moderate to the lower bound assumptions. The moderate assumption is that the unmatched firms, while being more innovative, would only respond to the ETS by the same absoluate size as the matched firms, i.e. 1.75 patents. With tobit modification, we can estimate the total amount of increase of 282 (90, 301) low-carbon patents, or 10% (3.04%, 10.94%) of the total of the ETS firms. For the lower bound, we assume that there is no effect on the unmatched ETS firms at all. Idealy, this assumption could be operationalized by creating a pseudo control for

each unmatched ETS firm, with the same numbers of pre-treatment and post-treatment patents between the pairs. But this would simply create zero values for the DID of unmatched firms, leading to too many zeros to estimate. So instead, we simply estimated the aggregate and percentage effects on all the ETS firms with the increase only from matched firms. The result is an increase of 66.5 (22, 71) low-carbon patents, or 2.23% (0.73%, 2.38%) of the total.

Table S15 (appears in the supplementary information): Inference for the aggregate effect on the whole sample.

	point estimate	95% confidence interval
For matched ETS firms		
individual effect	1.75	(0.5, 1.9)
aggregate effect	66.5	(22, 71)
percentage effect	117.7%	(21.8%,136.5%)
For all ETS firms assuming the same individual effect on unmatched firms		
aggregate effect	282	(90, 301)
percentage effect	10.13%	(3.04%, 10.94%)
For all ETS firms assuming no effect on unmatched firms		
aggregate effect	66.5	(22, 71)
percentage effect	2.23%	(0.73%, 2.38%)

Following the reviewer's suggestion, we added the results from bounding estimation to the main article. It reads "If we assume moderately that the same policy effect of 1.75 additional patents applied to these firms, the total effect on all the ETS firms would be 282 (90, 301) additional low-carbon patents considering data censoring at zero, or 10.1% (3.04%, 10.9%) increase (Table S15). If an alternative matching method based on propensity score is used to have most of the ETS firms matched, the estimated effect would be 0.75 (0, 1.9) additional low-carbon patents individually, 135 (0, 301) additional patents in total, or 4.6% (0, 10.9%) increase (Table S16). Even if we assume no effect on the 40% unmatched firms, an extreme case, the increase from the matched firms would still lead to 2.2% (0.73%, 2.38%) increase of all the ETS firms (Table S15)."

5. Interpretation of spillover estimates. I like the general approach to spillovers, but I think the description of the nature of possible spillovers is unnecessarily restricted, as is the interpretation of your findings. The authors speculate that non-ETS firms might be responding to the expectation of being under the ETS in the future. This is one valid hypothesis (although if firms are responding to expectations, I think you need to address the question of how much you are underestimating your main effect because the ETS firms started responding before the ETS started).

The issue of spillovers will be addressed below. For underestimation, it is not a serious issue because of our main matching strategy, as explained in our response to comment

#2: more than 60% of the matched pairs are between ETS firms and firms in the non-ETS low-carbon pilot region. In addition, we find that only 75 out of the 852 firm pairs, or 8.8%, used non-ETS firms subject to spillovers as defined in our main article.

To further confirm this, we strictly require ETS firms to be matched with firms in the non-ETS low-carbon pilot region, i.e. where there is no policy spillovers. There are 778 matched pairs, including 688 non-ETS firms with replacement. The point estimate and upper bound remain 1.75 and 1.9 patents, while the lower bound increases to 1 patent.

But spillovers can come in other shapes, e.g. non-ETS firms could respond to an ETS by reducing their innovation in the expectation that ETS firms will bear more of the innovation-burden, or non-ETS firms could respond by increasing their innovation to keep up with their more innovative ETS competitors, or by increasing their innovation to sell to ETS companies who now have greater demand for these technologies, etc. Each hypothesis potentially implies a different set of empirical patterns, and you would want to conduct an empirical test that discriminates between them. When you're just highlighting one hypothesis, it isn't clear whether your findings actually favour your hypothesis over the others.

We appreciate the suggested potential mechanisms of spillovers. Our intention was to test whether there was a general deterrence on non-ETS firms because of expectations in policy expansion. Based on this hypothesis the targeted firms were selected and the potential effects on them estimated and confirmed. It was not the reverse order in which interpretation came after results. We use this strategy rather than an extensive analysis of spillover effects because of the same concern that there would be so many channels of spillovers that we could not estimate each of them reliablely.

The alternative mechanisms that the reviewer suggests are not quite consistent with the evidence we can provide.

- "non-ETS firms could respond to an ETS by reducing their innovation in the expectation that ETS firms will bear more of the innovation burden". This mechanism suggests a negative spillover effect, but a positive one is revealed in the article.
- "non-ETS firms could respond by increasing their innovation to keep up with their more innovative ETS competitors". This mechanism could explain spillovers to large firms below inclusion threshold in ETS programs, but not those in non-ETS sectors, which are ETS sectors in the Shenzhen ETS. The Shenzhen ETS is the first launched program to create some policy expectations, and the most comprehensive one in sector coverage and number of firms included. But most of the firms in the Shenzhen ETS are much smaller in size than what we selected in the rest of the ETS region to test spillovers.
- We further tested the proposed mechanism explicitly. If the mechanism existed,

one would expect that spillovers should be more significant in industries with higher-level competition. We test this possibility by adding an interaction term between HHI and treatment (firm subject to spillovers) in regressions similar to that of Table 2. Table R8 below shows that the interaction term is nonsignificant while the spillovers alone (treatment) is.

- "by increasing their innovation to sell to ETS companies who now have greater demand for these technologies". This mechanism suggests a knowledge spillover effect, which takes longer time as we discussed in the article. In addition, this would not necessarily lead to an effect on large firms and no effect on small firms, as observed by our analysis.
- We further tested the proposed mechanism explicitly. If these firms subject to spillovers innovated to sell to ETS firms, we would expect that they had more patent transfer or licensing out. Table R9 shows that it is not the case for patent transfer. Due to limited activities (most of the firms didn't have any transfer or licensing out), we could not even get reliable estimation. With even fewer activities, licensing out cannot be estimated. The summary statistics in Table R10 shows the firms did not increase licensing out after the ETS, if not reducing the activity.
- A final note is that all the proposed mechanisms are in a form of knowledge spillovers, which would not be as prompt as policy spillovers. It would be interesting to test them in a longer term.

Table R8: Testing for spillovers driven by competition.

	(1)	(2)
treatment	0.0746**	0.244***
	(0.0379)	(0.0656)
treat*HHI	-0.0840	0.0995
	(1.302)	(1.302)
treat*energy		-0.0636
		(0.378)
treat*no patent=1		-0.243***
		(0.0758)
State-owned		-0.0348
		(0.0718)
Foreign-owned		-0.0511
		(0.0635)
matched firm pair dummy	yes	yes
Observations	2,148	2,140
R-squared	0.521	0.526

Table R9: Testing for spillovers driven by patent transfer.

	estimate	95% confidence interval	matched firm pair
reporting firms		not enough data to estimate	
large firms in ETS sectors	-11.5	(-20, 20)	1,430
small firms in ETS sectors	-10.5	(-20, 20)	2,142
large firms in Shenzhen sectors	3	(-20, 20)	1,074
small firms in Shenzhen sectors	12.5	(-20, 20)	1,411
co-patenters of ETS firms		not enough data to estimate	

Table R10: Patent license-out of firms subject to spillovers.

	liccense-out post-ETS			liccense-out pre-ETS		
	Mean	Max	Min	Mean	Max	Min
reporting firms	0	0	0	0.0039	1	0
large firms in ETS sectors	0	0	0	0	0	0
small firms in ETS sectors	0	0	0	0	0	0
large firms in Shenzhen sectors	0	0	0	0.0009	1	0
small firms in Shenzhen sectors	0	0	0	0	0	0
co-patenters of ETS firms	0	0	0	0.0132	1	0

All these results suggest that the mechanisms proposed by the reviewer are not likely what have driven the spillover effects. We do appreciate these suggestions, which help to advance the understanding of spillovers. One sentence has been added to the main article to reflect these additional thoughs and discussions: “The spillover effect was not associated with industry competition, patent transfer or license out, suggesting the unregulated firms’ own demand in response to the policy.”

In a similar vein, you basically find that a bunch of large non-ETS firms are increasing innovation. You jump to the conclusion that that’s because they expect to be regulated under an ETS in the future, but I see no particular justification offered for this interpretation. An alternative interpretation, also consistent with your findings, is that something altogether different is going on in the Chinese economy that is driving low-carbon innovation among both large ETS and non-ETS companies. In this interpretation, perhaps most of the effect you’re attributing to the ETS isn’t actually because of the ETS at all.

According our matching specification as explained previously, an ETS firm is always matched and compared to a firm of a similar scale (both market capitalization and number of employees), among other characteristics. So the estimated effect cannot be attributed to size.

To address this concern more explicitly, we match the ETS firms with the non-ETS

firms subject to spillovers and estimate the treatment effect. The result is likely an underestimation of the actual effect but helps to further address the above-mentioned concern. There are 686 matched pairs, including 374 non-ETS firms with replacement. The estimated effect is 1.5 (1, 1.9) patents.

Basically, I think spillovers are a really challenging topic to deal with. Although I like the general approach the authors take, I think they have not done the hard work necessary to show why their estimates should be interpretated as they have, rather than, say, interpreted in a way that undermines their main estimates.

We agree that spillovers is a challenging topic, which probably worths an article itself. As shown above, the potential concerns can be addressed by our original results and additional analysis. We appreciate these dicussions and have revised the section of spillovers in the main article to make the interpretation more consistent with the evidence that can support it.

6. Title. I think the title needs to be changed. The phrase “not yet by pricing” is ambiguous, and as my comments above indicate, you don’t really have a strong identification strategy that rules out pricing. Perhaps a phrasing focusing on your positive findings might be more appropriate: “Mass-based emissions trading induces low-carbon innovation in China”.

We agree. The title has been changed to “Emissions trading induces low-carbon innovation in China” without the expression about pricing. The finding about mass-based allocation is not included in the title, mainly because many readers may not understand what it means.

Minor comments

1. line 8: “significantly induced low-carbon innovation” suggests statistical significance, rather than substantive significance. Perhaps “induced significant low- carbon innovation” instead.

Our intention is to indicate statistical significance at the firm level rather than substantive significance, as the overall impact is limited. There is another reviewer’s comment below, which suggests that the use of “significantly” may give readers a wrong impression that the overall impact was grand (page 49-50). So it really depends on whether one focuses on the individual level or overall impacts and we do not intend to further emphasize the wording here.

2. line 10: “with policy expectation” suggests that ‘policy expectations’ is something you’re actually measuring. Better to describe what you’ve done so the reader can assess if they think it’s a persuasive test of your hypothesis.

The detailed description of what have been done is in the section of spillovers, which has been revised according to a comment above. For line 10 and similar descriptions in line 48, however, there is no room for detailed elaboration of what have been done. Therefore, "with policy expectation" is simply removed in the two places. In spillovers section, the expression has been revised to "…spillovers were limited to large unregulated firms in sectors already or likely covered by the ETS." In conclusion, the expression has been revised to "The policy-induced innovation effect extended beyond the regulated firms to large unregulated ones in sectors already or likely covered by the ETS."

3. line 32: You talk about the pilot programs at "independent." This word has a statistical meaning that is inappropriate for this context (see earlier discussion on sources of variation).

To avoid any chance of understanding "independent" in statistical sense while maintaining the original meaning, the sentence has been revised. It now reads "The seven ETS pilots are independently designed and operated, featuring a variety of differences and creating rich opportunities for policy evaluation and learning."

4. line 36: This is where you assert that you are the first to present firm-level evidence on this question, before offering some unclear distinction with previous work on line 50. Please write this is a more engaged, clear, and less territorial way.

We explain above our difference compared to the related literature and choice of wording in response to the first major concern of the reviewer. We intended to use the combination of "first" and "directly from emissions trading" to define your research in the context of the literature, following previous suggestions received. We are willing to revise it if confusion and discomfort were raised. The sentence in line 36-37 now reads "Here we present firm-level evidence of policy effects directly from emissions trading and differential program designs in China since 2013." A similar statement in the abstract has also been revised by removing "the first".

5. line 71: The authors are using the term "patent families" when they actually mean "triadic patents."

Thanks. "Triadic patent" or "triadic patent family" is a particular type of patent family and a more precise description of our measurement. As it often refers to patents filed at the JPO, the USPTO, and the EPO, using "triadic patent" requires some additional clarification. The sentence now reads "As a measure of high-value innovation, triadic low-carbon patents[21] filed jointly at the SIPO, the USPTO and the European Patent Office (EPO) …"

6. lines 109-114: For most of these robustness tests, it is clear what the authors conclude but totally opaque from the text what actual test has been performed. Please consider re-writing.

Some revisions are made in order to provide additional information about the robustness checks, while not to introduce extensive explanations to make it more difficult to follow. It now reads "The effect was consistent across different matching specifications (Table S8); scopes of low-carbon patents (Table S9); more restrictive matching only within ETS regions to eliminate other policy influences (Table S10); different baselines and samples to eliminate unobservable selection bias (Table S11); and an alternative estimation based on a common parametric DID (Table S12). The estimated effect also passed placebo tests, being unlikely a result of chance, any other omitted variable (Fig. S9), or regional and firm features (Table S13)."

7. line 121: Again, "If an alternative matching method is used..." In what way is it different from the main matching method? The reader doesn't know how to make heads or tails of this paragraph without consulting the SI.

It has been revised and reads "If an alternative matching method based on propensity score is used to …"

8. Figure 3.b.: A bar chart (rather than a pie chart) would allow you to also show the total magnitude of the induced innovation as well as confidence intervals.

Fig. 3b has been replaced by a bar chart.

9. line 146-150: If non-ETS companies innovate because they expect to become ETS companies in the future, that means your control companies are also increasing innovation and "... the individual effect of ETS firms would be greater than the estimation above." But it also means that the aggregate effect further inflated because the population of treated companies is larger than the set of ETS companies.

This is correct. It is stated before the potentially larger individual effect that "the scope of the effect would be beyond ETS firms to unregulated ones".

10. lines 154-168: If these groups of non-ETS companies are potentially contaminated controls, have you re-matched and re-estimated your main results when these contaminated firms are removed from the matching pool? How does this affect your results? Does it affect it in the way that you'd expect, given the hypothesised sign of the spillover?

As we explain in response to the major concern for spillovers above, it does not affect the estimation substantially as not many firms subject to spillovers are used as controls. In addition, we strictly require ETS firms to be matched with firms in the non-ETS low-carbon pilot region, i.e. where there is no policy spillovers. The point estimate and upper bound remain 1.75 and 1.9 patents, while the lower bound increases to 1 patent. The result has been added to the section of spillovers "To avoid underestimation of the direct effect, the ETS firms were matched with firms in the non-ETS low-carbon pilot regions, showing a similar effect of 1.75 (1, 1.9) patents."

11. Table 1: The last column lists the number of matched pairs, but not how many companies you started out trying to match in each category. The scope for selection bias is very different if you're matching 1% or 100% of companies in each category.

We present the number of firms subject to spillovers and number of matched firm pairs below in Table R11. It is more difficult to find matches for the large outliers – reporting firms, top-10 firms in ETS sectors, large firms in Shenzhen sectors, and co-pateners. It is relatively easier to find matches for firms that are not as big, i.e. top 11-20, bottom-10, and small firms in sectors covered by the Shenzhen ETS.

Table R11: Number of firm pairs to test spillovers before and after matching.

	Before matching	After matching
reporting firms	319	128
top-10 firms in ETS sectors	2003	1430
top 11-20 firms in ETS sectors	1654	1387
bottom-10 firms in ETS sectors	2547	2142
large firms in Shenzhen sectors	1680	1074
small firms in Shenzhen sectors	1718	1411
co-patenters of ETS firms (no caliper)	159	79
co-patenters of ETS firms (with caliper)	159	13

12. lines 207-222: Given my major comments about the source of identifying variation, you should be more careful in using causal language here like "reason" and "effect." At least, you haven't yet made a persuasive argument why these associations should be causally interpreted.

Most causal language has been revised with the exception for that related to mass-based allocation, considering the additional evidence from placebo tests and block randomization that supports the effect from mass-based allocation. Now the statement about price, auction, and energy-intensive firms reads: "A higher permit price, however, was not associated with more ETS-induced innovation, shown by a small, nonsignificant coefficient. Neither was auction significantly correlated with induced innovation. Consistent with these was the fact that ETS firms in energy-intensive

sectors, who should be more sensitive to carbon pricing, did not have significantly more innovation."

13. Table 2: It is unclear if the dependent variable, low-carbon patent, is referring to a difference between firms or a change over time. Or is it a DID perhaps?

Yes, this is a direct difference-in-differeces without tobit modification, because it is calculated before any estimation.

Response to Reviewer #2

Reviewer #2 (Remarks to the Author):

This is an excellent paper that brings together a unique dataset to analyse the effect of China's pilot Emissions Trading Systems on low-carbon innovation. It is the first paper to robustly evaluate the impact of China's carbon markets on low-carbon innovation, which has high scientific importance and huge policy implications given the recent launch of the nation-wide carbon market in the country and its future expansion to sectors beyond energy production. The conclusions are backed by solid evidence. The data is of high quality and the econometric analysis is well conducted.

Thanks for the nice comment. We respond to the remaining comments below point by point.

The paper presents three main results: (i) China's pilot ETS induced innovation in low-carbon technologies, as measured by patent filings in relevant technological fields, among the set of regulated companies; (ii) this effect was not associated with a decrease in patenting in other technologies (actually, the opposite is found); (iii) unregulated firms also reacted by filing more low-carbon patents, probably in the expectation of future regulation; and (iv) the impact was not found to be statistically greater in markets with higher carbon prices.

I think that the first 2 sets of results above are extremely solid. Result (iii) is very interesting, but I wonder how balance can be achieved if you match the largest (or top 10) firms in every ETS or Shenzhen sectors. If you systematically match these firms with smaller, could this not drive your results? Please provide also balance tests for this set of results, as the assumptions behind using matching are less obviously met given that you cannot exploit the same inclusion criteria.

Thanks for the nice summary. We agree that it is important to evaluate the balance for the matched firm pairs in different channels of potential spillovers. The quality of matching may be compromised because the "top" firms are outliers and may not be similar to other firms. The same rationale applies to bottom firms, too. It may not be a serious concern because firms subject to spillovers are usually matched to control firms outside the same ETS province/city usually in a low-carbon pilot provice/city without an ETS.

Below is a set of seven tables presenting balance test results for each of the seven channels as in Table S6. They show that reporting firms are well balanced; top and bottom firms in ETS sectors are balanced except for one variable – asset, employee, or output; lager firms in Shenzhen ETS sectors and co-pateners are not well balanced in

asset, employee, and output. One paragraph is added to the the method section of the main article to reflect that "the assumptions behind using matching are less obviously met" for testing spillovers. It reads

"A same estimation strategy was used to test policy spillover effects. It matched non-ETS firms potentially subject to spillovers – reporting firms, large firms in ETS sectors or in Shenzhen ETS sectors, co-patenters of ETS firms – with other non-ETS firms not subject to these influences. Unlike clear-cut inclusion criteria, however, spillovers take networked channels and can be ambiguous. Therefore, the same identification strategy may not be as strong as in the main estimation."

Table R12: t tests for key variables of reporting firms and matched controls.

	difference in the means	standard error	p-value
2011-12 low-carbon patenting	0	0.029	1.000
2011-12 total patenting	0.008	0.683	0.991
historic low-carbon patenting by 2012	0.000	0.036	1.000
historic patenting by 2012	0.000	1.063	1.000
2012 number of employee	184.172	118.904	0.123
firm age by 2013	0.469	1.002	0.640
2012 total asset (million RMB)	292.422	193.323	0.132
2012 output (million RMB)	211.726	185.529	0.255
four-digit industry sector	exactly matched		
low-carbon pilot region	exactly matched		

Table R13: t tests for key variables of top-10 firms in ETS sectors and matched controls.

	difference in the means	standard error	p-value
2011-12 low-carbon patenting	-0.0007	0.009	0.935
2011-12 total patenting	0.042	0.138	0.762
historic low-carbon patenting by 2012	-0.001	0.009	0.936
historic patenting by 2012	0.104	0.248	0.675
2012 number of employee	36.190	30.083	0.229
firm age by 2013	0.136	0.243	0.577
2012 total asset (million RMB)	43.259	64.347	0.501
2012 output (million RMB)	173.352	75.359	0.021
four-digit industry sector	exactly matched		
low-carbon pilot region	exactly matched		

Table R14: t tests for key variables of top 11-20 firms in ETS sectors and matched controls.

	difference in the means	standard error	p-value
2011-12 low-carbon patenting	0	0.006	1.000
2011-12 total patenting	-0.009	0.156	0.956
historic low-carbon patenting by 2012	0.000	0.007	1.000
historic patenting by 2012	0.002	0.224	0.992
2012 number of employee	20.825	19.833	0.294
firm age by 2013	0.010	0.235	0.966
2012 total asset (million RMB)	24.713	37.022	0.504
2012 output (million RMB)	-15.236	49.598	0.759
four-digit industry sector	exactly matched		
low-carbon pilot region	exactly matched		

Table R15: t tests for key variables of bottom-10 firms in ETS sectors and matched controls.

	difference in the means	standard error	p-value
2011-12 low-carbon patenting	0	0.005	1.000
2011-12 total patenting	-0.016	0.071	0.819
historic low-carbon patenting by 2012	0.000	0.005	1.000
historic patenting by 2012	0.001	0.115	0.990
2012 number of employee	-3.883	6.893	0.573
firm age by 2013	0.049	0.198	0.806
2012 total asset (million RMB)	-4.364	8.465	0.606
2012 output (million RMB)	-52.926	10.264	0.000
four-digit industry sector	exactly matched		
low-carbon pilot region	exactly matched		

Table R16: t tests for key variables of large firms in Shenzhen ETS sectors and matched controls.

	difference in the means	standard error	p-value
2011-12 low-carbon patenting	-0.0009	0.013	0.944
2011-12 total patenting	0.0009	0.163	0.995
historic low-carbon patenting by 2012	-0.0009	0.014	0.947
historic patenting by 2012	0.156	0.285	0.584
2012 number of employee	66.031	44.233	0.136
firm age by 2013	0.119	0.240	0.619
2012 total asset (million RMB)	55.725	18.581	0.003
2012 output (million RMB)	187.218	31.906	0.000

	difference in the means	standard error	p-value
four-digit industry sector	exactly matched		
low-carbon pilot region	exactly matched		

Table R17: t tests for key variables of small firms in Shenzhen ETS sectors and matched controls.

	difference in the means	standard error	p-value
2011-12 low-carbon patenting	0.0007	0.011	0.949
2011-12 total patenting	-0.015	0.101	0.883
historic low-carbon patenting by 2012	0.001	0.013	0.957
historic patenting by 2012	0.020	0.158	0.900
2012 number of employee	-8.578	9.853	0.384
firm age by 2013	0.060	0.221	0.786
2012 total asset (million RMB)	-2.540	6.772	0.708
2012 output (million RMB)	-37.017	9.538	0.0001
four-digit industry sector	exactly matched		
low-carbon pilot region	exactly matched		

Table R18: t tests for key variables of co-patenters and matched controls.

	difference in the means	standard error	p-value
2011-12 low-carbon patenting	0	0.109	1.000
2011-12 total patenting	0.385	1.769	0.830
historic low-carbon patenting by 2012	0.000	0.109	1.000
historic total patenting by 2012	2.077	4.770	0.667
2012 number of employee	47.769	78.328	0.548
2012 total asset (million RMB)	1.769	1.768	0.327
firm age by 2013	-160.656	313.447	0.613
2012 output (million RMB)	-162.899	108.845	0.148
four-digit industry sector	exactly matched		
low-carbon pilot region	exactly matched		

My main reservation regards result (iv). This finding comes from a regression where the ETS status is interacted with multiple programme design characteristics as well as firm characteristics. Four reasons may explain the lack of statistical significance for the interaction term between ETS and the price, and for other interaction terms more generally: first, I expect many of the programme design features to be highly correlated with each other (a correlation matrix in the supplementary material would be welcome to check whether this is the case);

We agree that lack of statistical significance for price and other program features may not be sufficiently enough to support a causal conclusion regarding these features. Meanwhile, the evidence for a causal effect from mass-based allocation seems strong, especially considering the additional evidence in Fig. R1-R6 based on placebo tests and block randomization. Below are point-by-point responses to the explanations.

Table R19 shows the correlation matrix. It is not as seriously correlated as one would expect, considering that most features vary at the program level except mass vs rate-based allocation within programs. But we think the first explanation is still a very reasonable assumption in the sense that the choices of design features by an ETS program are not independent but rather nested. In other words, an ETS selected the coverage of industries, emission threshold for inclusion, allocation method, aution, etc. which collectively determined market price and active rate. Therefore, in empirical terms, the research design cannot be treated as quasi-experimental for each of program features indvidually. While we cannot do too much to investigate each feature, we provided additional analyses regarding the main finding of mass-based allocation in Fig. R1-R6 as suggested by reviewer #1.

Table R19: Correlation matrix of design and operational features of ETS.

	mass	price	auction	active rate	cap	CCER
mass	1.00					
price	-0.74	1.00				
auction	0.29	-0.32	1.00			
active rate	-0.82	0.91	-0.08	1.00		
cap	-0.60	-0.59	0.43	-0.58	1.00	
CCER	-0.86	0.81	-0.29	0.92	-0.59	1.00

second, I expect many of these programme features to be correlated with firm characteristics, but I couldn't find the list of firm characteristics included as controls (in particular, I wonder whether the statistically significant difference between mass-based and rate-based is not driven simply by a greater effect among manufacturing firms compared to firms in the energy sector. Do you have sector dummies and region dummies as controls?);

As explained above, it is a reasonable assumption. It has been taken into account in the regressions:

- Firm chacteristics are included as control variables in two specifications of Table 2 in a way similar to the matching variables. They include total asset, number of employees, age, pretreatment patents and low-carbon patents, historic patents and low-carbon patents.
- Additionally, we add matched firm pair dummies – a dummy variable for each

firm pair to further control for firm-level differences so that the DID is in fact a matched DID. This means industrial sectors are also controlled for because it is an exact match condition.

- Considering that firms in the energy sector are usually under rate-based allocation, Column 3 of Table 2 excluded those firms from the sample, where the results are still the same.

third, there is little variation to exploit since the average price did not differ much across ETS programs (with the exception of Shanghai and Beijing);

A major issue with the average price is that it is at a very low level – below 10 US dollars – across all the seven markets. Therefore, even though there is some variation we can explore across markets, firms under the highest price may still not have enough incentives to innovate.

fourth, what matters for innovators is the (unobserved) expected future price of carbon on the market, not the current spot price.

We agree. The average market price is just a proxy. It is arguably a better proxy than the price of single transactions, which wold be endogenously determined by the firms involved (and cannot be observed in our research). But still, the expected future price would be a preferred measurement in case an exogenous shock that affects firm expectation could be used for identification. Unfortunately, we cannot find such a shock as a new identification strategy for our empirical investigation.

For these reasons, and unless you can make the finding of an absence of a price effect more robust, I would make this result less prominent (in particular, not feature in the paper's title), as I think it is not that strongly supported by the analysis. For a wide-audience journal like Nature Communications, the main result on the impact of the Chinese ETS on low-carbon innovation is sufficient anyway in my opinion.

Thank you for the suggestion. We agree with the argument above and are willing to make the conclusion about the price effect less prominent. Now the title reads "Emissions trading induces low-carbon innovation in China". The causal language that was used to describe results regarding price and other program featurs except mass-based allocation has been revised as correlational.

Some less important comments follow:

1. It would be interesting to know how the pilot schemes were selected among potential candidates and what are the specificities of the chosen regions and cities compared to the rest of China, in terms of carbon emissions, emissions intensity and low-carbon innovation prior to the launch of the programmes. This would give some

indications as to whether the results might generalise to the whole of China.

There are three related issues here. The first is about the policy-making process that leads to the selection of the seven provinces and cities. The second is the representativeness of these regions. The third is the generalizability of the research findings.

The general policy process of ETS pilots is transparent, but the detailed decisions not so much, which could be done as a separate research project. The ETS pilots are part of the broader policy experimentation of low-carbon pilots the national government started in 2010. Local governments are encouraged to apply to the National Development and Reform Commission (NDRC) for experimenting different kinds of low-carbon policies. The NDRC approved five provinces and eight cities in the first batch, including Guangdong, Hubei, Chongqing, Tianjin, and Shenzhen. Shanghai and Beijing are selected by the NDRC in the second batch. There is no explicit rationale for the selection by the NDRC that can be observed.

The representativeness of the seven regions differs. Guangdong, Hubei, and Chongqing are nice representative of differet areas of China, which is extremely heterogenous. Their representativeness can be confirmed by an on-going evaluation of us based on synthetic control. Guangdong has a more advanced industrial, export-oriented economy representing the provinces along the east coast. Hubei is in the middle of China and is representative of provinces in the middle and northeast areas. Chongqing is politically a provincial-level city but is actually very similar to the other west provicnes of lower development levels. The other four cities – Beijing, Shanghai, Shenzhen, and Tianjin – are not well representative. Three of them – Beijing, Shanghai, and Shenzhen – are full of innovation activities from coporate research centers, universities, and research institutions. They are outliers in innovation.

While generalizability is related to representativeness of research areas, it can be further improved in research. There are two reasons for nice generalizability of the findings. First, because of the matching process, the outliers are dropped out of the research and the remaining sample is well representative of the industrial firms in China. To confirm this, we constitute bins, each representing a patenting value of the matched ETS firms, and calculate the percentage of firms falling in these bins from the whole sample (the merged dataset from firm and patent data). Table R6 shows that the matched ETS firms account for the patenting activities of more than 85% of all patenting statistics in ETS regions, and are even more representative at the national level, covering the patenting level of more than 95% of firms. Our main finding of ETS-induced low-carbon innovation from industrial firms should be widely applicable in China.

Second, the investigation of design features also strengthens generability. The particular

finding that mass-based allocation induces significantly more innovation while rate-based allocation doesn't is not context-specific and can be extended to the design of other ETS programs.

Table R6 (appears previously): Representativeness of matched ETS firms.

	pre-treatment low-carbon patenting	pre-treatment total patenting	historical patenting
in ETS region	94.9%	90.2%	86.2%
in low-carbon pilot region	98.6%	96.6%	95.3%
in mainland China	98.9%	97.3%	96.4%

2. Your analysis of the impact on non-ETS firms is extremely interesting, but could you give an indication as to how this effect compares with the effect on the set of regulated firms, in terms of the number of total patents? It seems that the total effect on unregulated firms might be greater than the total effect on ETS firms.

There are a few reasons that make an explicit calculation and presentation of the aggregate innovation effect from spillovers not preferred. First, there are different channels of spillovers and the effort to find the channels of spillovers is not likely exhaustive. Second, the scale and channels spillovers may change through time. On the one hand, knowledge spillovers, which are not likely salient in our time frame, may become more significant in longer terms. On the other hand, policy spillovers may also change as firms' expectation changes. Third, because of the nature of spillovers, it is also more difficult to find suitable counterfactuals for reliable estimation, compared to the estimation of the main effect. Finally, the applicability of the findings about aggregate spillover effects would be limited, especially when the program coverage increases.

Because of these reasons, the authors wanted to be cautious in estimating and interpreting the spillover effect. We hope that the implications are relevant and not excessively interpreted. Therefore, what we intend to conclude is that 1) there is policy spillovers, so that the focus should not be only on the regulated firms; 2) spillovers are not likely to change to the overall impact of the ETS pilots as being limited. The spillover effect is likely of a similar magnitude as the main effect at the current stage, but this rough guess seems not to provide additional value to the current discussion in the article.

3. That ETS programs also affect unregulated companies has implications, as you note, for the baseline results, which might be underestimated. Can you give an indication on the size of this bias? In particular, are 'large firms in ETS sectors' used as controls in the baseline results? Does the baseline treatment effect increase if you restrict control firms to firms less likely to be regulated in the future?

The fact that there are spillovers may affect the result of the main estimation. But it does not affect our estimation seriously, because most of the matched control firms are not subject to spillovers (at least not in the channels we investigated).

To further confirm this, we strictly require ETS firms to be matched with firms in the non-ETS low-carbon pilot region, i.e. where there is no policy spillovers from the channels we investigaged. This leads to 778 matched firm pairs, including 688 non-ETS firms with replacement. The point estimate and upper bound remain 1.75 and 1.9 patents, while the lower bound increases from 0.5 to 1 patent.

4. The merging between SIPO and ASIF only allows you to match 2 million patents out of the 8 million patents filed by firms. The ratio for low-carbon patents is similar (147k patents against 546k). Your sample is large enough, and I doubt that the matching quality would vary systematically around the inclusion threshold, but can you say more about the matching quality and the potential consequences for your analysis?

We appreciate the opportunity to discuss our efforts to merge patent and firm datasets, the reasons for unmatched patents, and the implications for our analysis. The identification strategy based on matching – in case this also relates to your comment – is addressed in our response to the major comment #2 of reviewer #1 (pages 8-9). In short, we explored two margins, i.e. matched firm pairs with location difference and matched firm pairs with emission difference in the base year, with the matched sample mostly from the former. The former nicely explores the fact that a matched control would have been regulated in case the ETS had a broader sectoral coverage or lower emission threshold for inclusion (e.g. glassmaking is covered by Shenzhen ETS but not by Guangdong ETS; steel-making is covered by both but Shenzhen has a lower inclusion threshold to regulate smaller firms).

The matching rate of our merging process is comparable to the most recent effort to link the Annual Survey of Industrial Firms (ASIF) and the patent entries from the State Intellectual Property Office (SIPO). For example, He et al. (2018) matched 1,113,588 patent entries with the ASIF 1998-2009. In comparison, we matched 1,271,034 patent entries with the ASIF 1998-2010. To improve the matching rate, we used a fuzzy matching technuiqe that breaks the names of firms and patent applicants into pieces of information. This accounts for the discretion in filling out firm names, especially when responding to the ASIF. Similar strategies have been used to link multiple datasets, for example, the NBER patent applicant and Compustat ID (Autor et al., 2019).

Having said this, we agree that it is helpful to diagnose reasons for unmatched patents. There are mainly six reasons for a patent entry not being matched with a firm in the ASIF:

- The eight million patents are filed by domestic and foreign firms. Because *foreign* firms are not covered in the ASIF, patents filed by them cannot be matched. They are of course irrelevant to our research.
- Patents filed by firms that *exited* before 2011 are not matched. While this is a result due to the merging of the ASIF of different years, the outcome is not relevant to our research.
- The coverage of SIPO patents is more up to date than the ASIF, which covers till 2013. Therefore, patents filed by firms established after 2013 cannot be matched. The *newly established* firms are not likely included in the ETS. Even if they were, it would not be possible to evaluate their effect, because there is no pretreatment status necessary to the matching process. The outcome would not affect our analysis.
- Patents filed by *service* firms are not matched, because the ASIF covers the secondary industry only. While a small amount of service firms are included in the ETS, the majority of ETS firms are industrial. Unless a more comprehensive dataset than the ASIF existed, we would have to drop these service firms out of our analysis.
- Patents filed by industrial firms that are not included by the ASIF cannot be matched. The ASIF covers all the state-own firms and other firms that are above-scale, which means more than five million RMB annual revenue before 2010 and more than 20 million afterwards. They are not likely included by the ETS pilots because of their *small size*, but would cause selection bias in case they were included.
- Finally, patents may not be matched due to our matching *method*. This is probably the main concern of the reviewer.

The first four reasons of unmatched patents are either irrelevant to our research (foreign or exited firms) or not a serious concern (service or newly established firms). But the latter two may affect the reliability of our results. To evaluate to extent to which the latter two may affect sample selection, we estimate the percentage of unmatched firms for each reason whenever possible, based on different kinds of dataset.

First, according to the address of patent applicants, foreign applicants with addresses outside mainland China can be recognized. They account for 1,936,891 previously unmatched patents, or 23.4% of the total.

Second, using the subset of the ASIF before 2011, we are able to match previously unmatch patents from firms that exited before 2011. They account for 1,271,034 previously unmatch patents, or 15.4% of the total. Notice that this is a lower bound estimation, as there would also be some firms that exited for which our method failed to match.

Third, using a comprehensive dataset we recently acquired, we are able to estimate the newly established firms in 2014 or after that filed patents. This is a fetched dataset of firm registration information from the State Administration for Industry and Commerce, including firm name, date of regristration, and business area. While some entries may have been lost in the fetching process, the dataset covers almost the whole population of business in China, including 20 million firms. With the dataset, some previously unmatched patents can be matched with firms regristered in 2014 or later. They account for 437,523 previously unmatched patents, or 5.29% of the total. Similarly, this is a lower bound estimation, considering that our method may not be able to match some of the new firms.

Fourth, the remaining 2,622,752 unmatched patents can be further matched with firms in the new dataset. As a result, 2,064,362 additional patents are matched. Table R20 shows the distribution of the newly matched patents across business sectors, and Table R21 shows the distribution of the matched firms across business sectors. About 56% patents are from non-industrial firms. They account for 1,158,083 previously unmatched patents, or 14% of the total.

Fifth, the remaining 906,279 patents from manufacturing, utility, or mining sectors in Table R20 roughly represent firms not covered by the ASIF because of their smaller size. This is a rough estimation assuming that name entries in the ASIF and the firm registration system is the same. They account for 11% of the total patents. Table R21 indicates that the patents are owned by 81,847 firms, which on average have 11 patents. This is much larger than the average statistics of the matched ASIF firms. Considering that the firms are not included because of their small size (measured by annual revenue), these firms are in fact atypical industrial firms specialized in innovation rather than production. Therefore, they are not related to our focus on the ETS firms and potential candidates to the ETS.

Finally, there are only 558,390 unmatched patents left, after taking into account the newly matched or diagnosed patents from the previous five categories. If the rate of 56% patents from non-industrial firms (and 44% industrial) in the fourth step still applies to the remaining patents, the patent entries unmatched with the ASIF because of matching method would be only about 246,000. This means that our method only failed to match 11% of the patents (246,000 unmatched vs 2,000,120 matched). Even if the limited coverage of the ASIF is also considered, the patents that are unmatched in the merging process are in fact 36% (246,000 unmatched, 2,000,120 matched, and 906,279 newly matched), the majority of which turn out to be not relevant to our research. We can therefore conclude that the reliability of our findings is not likely affected by the merging process.

Table R20. Sectoral distribution of the previously unmatched patents.

industry	frequency	percent	cumulative percent
Manufacturing	861583	41.7%	41.7%
Scientific research and technical services	520607	25.2%	67.0%
Wholesale and retail services	234681	11.4%	78.3%
Information transmission, software and information technology services	143254	6.9%	85.3%
Construction	134087	6.5%	91.8%
Leasing and business services	36796	1.8%	93.5%
Production and supply of electricity, heat, gas and water	35252	1.7%	95.3%
Agriculture, forestry, animal husbandry and fishery	32981	1.6%	96.9%
Business services	12751	0.6%	97.5%
Management of water conservancy, environment and public facilities	12660	0.6%	98.1%
Services to households, repair and other services	9467	0.5%	98.5%
Mining	9444	0.5%	99.0%
Transport, storage and post	7369	0.4%	99.4%
Financial intermediation	6592	0.3%	99.7%
Real estate	2769	0.1%	99.8%
Culture, sports and entertainment	2396	0.1%	99.9%
Hotels and catering services	857	0.0%	100.0%
Health and social work	296	0.0%	100.0%
Education	269	0.0%	100.0%
Pubic management, social security and social organizations	64	0.0%	100.0%
Other	187	0.0%	100.0%

Table R21. Sectoral distribution of the previously unmatched firms.

industry	frequent	percent	cumulative percent
Manufacturing	78160	41.5%	41.5%
Scientific research and technical services	47825	25.4%	66.8%
Wholesale and retail services	29598	15.7%	82.5%
Information transmission, software and information technology services	10619	5.6%	88.2%
Construction	7177	3.8%	92.0%
Agriculture, forestry, animal husbandry and fishery	4284	2.3%	94.2%
Leasing and business services	2905	1.5%	95.8%
Business services	1774	0.9%	96.7%
Management of water conservancy, environment and public facilities	1294	0.7%	97.4%
Services to households, repair and other services	1135	0.6%	98.0%
Production and supply of electricity, heat, gas and water	1117	0.6%	98.6%
Transport, storage and post	782	0.4%	99.0%
Culture, sports and entertainment	471	0.3%	99.3%
Mining	398	0.2%	99.5%
Real estate	396	0.2%	99.7%
Financial intermediation	340	0.2%	99.9%
Hotels and catering services	171	0.1%	99.9%
Education	35	0.0%	100.0%
Health and social work	25	0.0%	100.0%
Pubic management, social security and social organizations	8	0.0%	100.0%
Other	40	0.0%	100.0%

Response to Reviewer #3

Reviewer #3 (Remarks to the Author):

This article uses patent analysis to determine the innovation impact of the seven ETS pilot schemes in China. Methodologically, it builds on comparable studies conducted for the EU ETS – a quasi-experimental design matching comparable ETS and non-ETS firms. The paper finds a positive innovation impact of the pilot ETS, increasing low-carbon innovation by a few percent (even for firms who might be expecting to be included in the ETS in the future). The analysis also demonstrates that the (free) allocation method matters, as only mass-based allocation is associated with a positive innovation impact. This is a critical finding as the national ETS in China has adopted the rate-based approach for which the study does not find an impact on low-carbon innovation, which can be interpreted as a critical shortcoming for the dynamic efficiency of the national ETS in China, and thus the manuscript is of high policy relevance. The paper will be of interest to all academics and policy makers working on emissions trading, both in China and beyond. While the analysis appears sound and the article is well written, the communication could benefit from some improvements to more clearly and more directly get across the main findings and implication (see point 1 below). In addition, the embeddedness of the study into multi-disciplinary studies investigating the innovation impact of the EU ETS should be improved (rather than focusing on environmental economics and quantitative studies only) which would also help with more nuanced interpreting and critically reflecting upon the findings (see point 2). Overall, I see great value of the study to be published in Nature Communications.

Thanks for your nice summary and comments. Below are our point-by-point responses to the detailed comments.

Shortcoming 1: Clearer communication of findings and implications

- As the difference of a positive innovation impact of mass-based allocation vs no impact for rate-based allocation is a key finding with key policy implications for the national ETS, this needs to be communicated more clearly. First, the terms mass-based and rate-based need to be introduced fairly early on in the article, defining them and summarizing expected differences in innovation impact based on theory and other empirical evidence.

We agree that the result of mass-based vs rate-based allocation is a key finding and it helps to deliver the message more clearly by introducing the difference in allocation and associated implications earlier in the article. Several changes are made accordingly.

In introduction where the two terms first appear, they are explained rather than simply

mentioned. The sentence now reads "The effect was driven by firms that were subject to mass-based allowance allocation where the number of allowances was pre-established before a compliance cycle; the effect was not significant among firms subject to rate-based allowance allocation, where the number of allowances was updated according to the actual output."

In the section of potential mechanism when the two terms are introduced again with pricing and other program features, the terms were simply mentioned in one sentence at the beginning and explained later on after the results were presented. In the revised version, the terms are now explained by a separate paragraph toward the beginning of the section. The paragraph reads:

"Allowance allocation scheme is also an important feature of an ETS and may affect the degree of innovation[6,37]. In a typical cap-and-trade system, the amount of allowances is pre-established before a compliance cycle, i.e. mass-based. For political, competitiveness, or equity concerns, however, a rate-based system (also known as tradable performance standard) is often used by updating the number of allowances according to the actual output[38]. The former can achieve the social optimum, at least in theory, by setting an emission level to have the marginal abatement cost equal the marginal cost of the externality. Its efficiency also helps to link national and regional programs for global mitigation efficiency[2]. In comparison, rate-based allocation subsidizes output by allowing additional emissions and compromises cost-effectiveness[39,40], which is exacerbated with heterogeneous benchmarks[41]. The innovation impact from the two methods, however, is likely ambiguous[6,38]. ETS pilots varied in their use of allocation methods, and usually applied different methods to different industries, which may lead to heterogeneous induced-innovation effects."

Second, in your results and discussion you should explain better the mechanisms behind output-updated allowance allocation and why it is creating an additional subsidy (p. 9 lines 225ff), so that the reader has a clearer understanding of the underlying mechanisms.

It takes analytical models and simulations to explain why this is the case – several papers are dedicated to the influences from rate-based (output-updated) allocation. Neither is it easily explained in one or two sentences, nor it is necessary to do so. Instead, we try to revise and make the expression easier to follow and direct the readers to the appropriate references when they need more information. Now it reads: "rate-based allocation subsidizes output by allowing additional emissions and compromises cost-effectiveness[39,40], which is exacerbated with heterogeneous benchmarks[41]."

Finally, and perhaps most importantly, the last sentence of the study/conclusion and abstract shall state much more clearly that based on this study no positive impact on

low-carbon innovation can be expected from China's national ETS, and discuss why this is a problem for long-term climate mitigation, leading to a clearer formulated policy recommendation.

We appreciate the suggestion to further highlight one of our key findings and its policy implication. The sentence about China's nationa ETS has been revised to "China's national carbon market has adopted a rate-based approach with the number of allowances being updated by firms' actual output[47], which had no effect on inducing innovation in the ETS pilots." However, we did not make our final recommendation stronger, as the evidence provided by our research – induced innovation effect via certain allocation method – is one of the dimensions to consider in choosing allocation methods. Political acceptability, competitiveness, and equity, for example, are also important dimensions to consider.

- More generally, you rightly discuss the importance of actual ETS design for its innovation impact (following the line of argument of e.g Vollebergh and Kemp & Pontoglio that the innovation impact is more dependent on design features than instrument types). I would recommend that you should take care in defining your design features and other variables which you introduce on p. 7 on line 200 as program composition, design and operation, and to briefly outline which innovation impacts you would expect of these based on the extant literature.

Some revisions have been made following the suggestion mostly in the previous paragraphs, to indicate potential innovation impacts from a carbon price and output-updated allowance allocation, respectively. Given that these issues are elaborated in the previous paragraphs, the sentence that the reviewer refers to is made more concise to focus on pricing and allocation as two main aspects to test. Now it reads "We evaluated whether the induced-innovation effect was dependent upon pricing, allowance allocation, and related features that vary across programs and industries."

- The wording that the pilot ETS have "significantly induced low-carbon innovation of ETS firms" (in abstract and elsewhere), while technically not wrong, is implicitly making the effect to appear to be grand, while a careful reader will see that indeed it has been quite limited. While the authors later also state that "the overall impact was limited" what sticks with the reader is the impression of a grand effect suggested by the word "significant". To clarify this, the authors should be careful in their wording (to avoid misunderstandings) and should absolutely include the very useful percentage figures they have provided in their analysis in their abstract and conclusion, alluding also there to the additional patents of 4.6%-10.1% (depending on matching method) and to the low share of the low-carbon patents of 1% associated with the ETS. These figures much more clearly demonstrate the actual extent of the innovation impact. The positive impact can be identified through sophisticated matching, but it is really at the

moment still very miniscule overall. This needs to come across very clearly in all of the article, also if a reader were just to read the abstract.

There are several ways to interpret the results:

- for individual ETS firms in our matched sample (which tends to be representative of the whole population as explained by Tables R5-R6), the effect is extremely significant and doubles those firms' patenting;
- for all the ETS firms including the unmatched ones (which are usually outliers with substantially more patents than average firms and therefore unable to be matched), the effect becomes smaller under moderate assumptions that the unmatched firms would not be as responsive. But the effect is still substantial and significant (the number of 4.6%-10.1% as the reviewer indicated);
- for the overall impact, the effect should be compared to the regional total patents, but at the same time spillover effects should be taken into account. The effect on ETS firms contributed to 1% to the regional total without considering spillovers. The spillover effect tends to be of a similar magnitude, but cannot be estimated accurately (see our response to the second minor comment of reviewer #2). As a result, we could not provide a percentage of the overall contribution of the ETS. Instead, we provide different interpretations in the results section with detailed contexts.

As can be seen, the interpretation of the results is context-specific. Accuracy and concision cannot be achieved at the same time to be reflected in the abstract. So we prefer to present both the significant effect on ETS firms and the limited overall effect in the abstract, and explain in details in the main text. Another reviewer also had a comment on the expression but suggested us emphasize substantive significance (page 29). So we think it really depends on whether one looks at the individual firm effect or the overall impact.

- You argue in the conclusion on p. 9 in line 247 that a broader program coverage is needed to increase policy impact, but your earlier findings even more so point to a different allocation approach being needed than the one adopted in the national ETS, so I would mention this alongside program coverage, before you will then, in your last paragraph, unpack the issue of mass-based allocation as driver for low-carbon innovation (not rate-based). Please ensure to state this problem with the national ETS more clearly.

This comment is similar to another one above to suggest us emphasize the issue with rate-based allocation adopted by the national ETS. We explained in response to the other comment that because the choice of allocation methods should consider multiple factors, our result only provides one factor in favor of mass-based allocation. Following that comment and suggestion, we revised the second paragraph of the conclusion

section to make it more explicit that rate-based allocation did not induce innovation according to the experiences from ETS pilots. We would prefer to keep that information in the second paragraph of the conclusion, and not to state the same information in the first paragraph, too. This helps to separately highlight our key findings – main effect, spillover effect, and heterogeneous effect from allocation – as well as their associated implications. A separation of positive findings (induced innovation effect of the ETS, need for expansion) from the heterogeneity seems helpful, as policy recommendations from the latter (switch from rate-based to mass-based) depends on the former.

- In light of these comments, consider changing the title of your manuscript so that the reader will already know: limited, but positive innovation impact, but only for mass-based allocation.

The title has been changed to "Emissions trading induces low-carbon innovation in China". The revised title does not include the previously emphasized price mechanism, as well as mass-based allocation or limited impact. There are a few reasons for which we do not want to stress on the latter two.

The meaning of mass-based allocation takes efforts to let readers understand as the reviewer suggested above, particularly for those unfamiliar with an ETS. Using the specific term directly in the title may therefore cause confusions. In addition, the specific term, when overly emphasized in the title, may cause one to ignore other findings presented in the submission.

The magnitude of impacts, as we explained above, depends on the unit being affected. The effect at the firm-level on an average firm is really signiciant. The overall impact at the regional level is limited, but this is mainly because the program coverage is limited. Too much emphasis on the limited impact might bring readers an inaccurate impression that the ETS would not be a promising policy instrument for low-carbon development.

Shortcoming 2: Broader multi-disciplinary embeddedness in previous literature on innovation impact of emissions trading schemes

- Not only environmental economists but also innovation and transition scholars have investigated – with various qualitative and quantitative methods – the innovation impact of emission trading systems, perhaps mostly for the EU ETS. However, this literature is not included in the current manuscript, which is a major shortcoming as the totality of studies have enabled a very nuanced and deep understanding of how emission trading systems influence innovation activities. For example, for the EU ETS a recent review on studies investigating its innovation impact by Rogge might be helpful in this regard. There are also a number of studies who have investigated the Chinese ETS pilots in terms of innovation impact as well as the design process of the national scheme based

on lessons-learned from the pilots (e.g. Shen, Duan et al). Omitting this broader ETS & innovation literature is problematic – particularly for a multidisciplinary journal as Nature Communications – and leads to shortcomings in the line of argument and in the interpretation of the findings.

We appreciate that the reviewer kindly direct us to the relevant literature. We would be happy to engage the related references broadly, and at the same time make sure that the paper builds on or connects to the cited references. To do so, we evaluated all the papers – thoses cited by the previous version of the submission, suggested by the reviewer, and published recently during our submission process – to see whether each of them should be cited in the revised version.

Particularly, Rogge et al. 2011 has been cited in response to another comment below. Several new papers about China's national ETS published during our submission process have also been cited:
Lin, S., Wang, B., Wu, W. & Qi, S. The potential influence of the carbon market on clean technology innovation in China. Climate Policy 18, 71-89, (2018).
Pang, T., Zhou, S., Deng, Z. & Duan, M. The influence of different allowance allocation methods on China's economic and sectoral development. Climate Policy 18, 27-44, (2018).
Stoerk, T., Dudek, D. J. & Yang, J. China's national carbon emissions trading scheme: lessons from the pilot emission trading schemes, academic literature, and known policy details. Climate Policy 19, 472-486, (2019).

- For example, on p. 3 in line 90ff the authors make a generic statement about the superiority of an ETS in terms of inducing innovation – however, this has been heavily debated in the broader literature, so a more broadly informed manuscript would require a more nuanced formulation of this (theoretically) claimed superiority of ETS.

We agree that the comparative statement in the original submission may not be universally held in all the situations, as reflected in the literature. The innovation effect of an ETS as relative to alternative policy instruments depends on other factors. And the expression does not need to be a comparative one about the relative strength of innovation effects of alternative policy instruments, as its main objective is simply to introduce the innovation effect of an ETS besides other policy effects. Therefore, it has revised as "An ETS is often considered to induce technological innovation[6] and adoption[27], besides the main effect to regulate polluting activities at the efficient level."

- Another example is the following sentence in lines 93f which makes the important statement that empirical evidence has shown that the actual innovation impact of an ETS depends on a range of factors, but no study is cited for this, nor is this further explained. However, as the study later picks up on the design questions it would be

advisable to summarize the state of the art on the empirical findings regarding the evidence for price/stringency, allocation mode (auctioning/types of free allocation). Also, interaction effects with other policy instruments would be worthwhile to report so as to be able to pick up on this in the discussion of your own findings on this matter, but there is no inclusion of references on policy mixes and instrument interactions (e.g. Sorrell, del Rio).

On the empirical side, the related references have been cited to our limited knowledge, i.e. Calel & Dechezleprêtre 2016; Fowlie et al. 2012; Cui et al. 2018; Feng et al. 2017. Of course, we would appreciate suggestions along this line.

On policy design, policy mix, and interactions, several related references have been cited to support the argument. Thanks for the suggestions.
del Río, P. On evaluating success in complex policy mixes: the case of renewable energy support schemes. Policy Sciences 47, 267-287, (2014).
Rogge, K. S., Schneider, M. & Hoffmann, V. H. The innovation impact of the EU Emission Trading System — Findings of company case studies in the German power sector. Ecological Economics 70, 513-523, (2011).
Sorrell, S. & Sijm, J. Carbon Trading in the Policy Mix. Oxford Review of Economic Policy 19, 420-437, (2003).

- Another example for this is the argumentation on p.7 on the expected influence of the carbon price on innovation activities of ETS firms (lines 193-197) which only argues with spillovers, while neglecting evidence that has pointed to threshold effects (e.g. 30 Euro EUA prices in the EU ETS) or differences in paying for permits vs receiving revenue from freed ones. You later say that a higher permit price was not a reason for ETS-induced innovation (lines 206ff) but we read too little about whether this finding can be expected to be generic or whether it is likely arising from (too) low prices, too little differences between the pilots, and a lack/neglect of auctioning in the pilots designs.

Our intention is not to interpret the lack of a price effect as a generic one potentially applicable to other ETS. Rather, it is very likely specific for the Chinese ETS pilots because of the low-level carbon price and limited trading (lack of liquidity). The explanations were given after the result was presented in the original submission.

To avoide confusion, we add the point in discussion of the price effect before presenting the results. So after the discussion of the evidence from spillovers, it reads "The [price] mechanism may also be compromised by the overall low price and limited transaction."

Overall, I find your study very interesting from an academic viewpoint and politically highly relevant, and thus my comments are limited to these two main points, and are

meant to improve the embeddedness in the wider literature which has investigated the innovation impact of ET schemes, and finetune the clarity of how you communicate your findings and their implications

Thanks for the nice suggestions. We are happy to address further comments and suggestions.

References

Autor, D., Dorn, D., Hanson, G.H., Pisano, G., Shu, P., 2019. Foreign Competition and Domestic Innovation: Evidence from US Patents. American Economic Review: Insights forthcoming.

Calel, R., Dechezleprêtre, A., 2016. Environmental Policy and Directed Technological Change: Evidence from the European Carbon Market. Review of Economics and Statistics 98, 173-191.

Caliendo, M., Kopeinig, S., 2008. SOME PRACTICAL GUIDANCE FOR THE IMPLEMENTATION OF PROPENSITY SCORE MATCHING. Journal of Economic Surveys 22, 31-72.

Cui, J., Zhang, J., Zheng, Y., 2018. Carbon Pricing Induces Innovation: Evidence from China's Regional Carbon Market Pilots. AEA Papers and Proceedings 108, 453-457.

Feng, C., Shi, B., Kang, R., 2017. Does Environmental Policy Reduce Enterprise Innovation?—Evidence from China. Sustainability 9.

Fowlie, M., Holland, S.P., Mansur, E.T., 2012. What do emissions markets deliver and to whom? Evidence from Southern California's NOx trading program. The American economic review 102, 965-993.

He, Z.-L., Tong, T.W., Zhang, Y., He, W., 2018. A database linking Chinese patents to China’s census firms. Scientific Data 5.

Reviewers' comments:

Reviewer #4 (Remarks to the Author):

Reviewer 3 (Substitute)

Note: This second review focuses on how the authors acknowledge and incorporate the first review by the original reviewer (Reviewer 3). Therefore, this is not a full review and instead is meant to be a focused evaluation of the following specific points raised by Reviewer 3. The original reviewer's main concerns relate to

- Clearer communication of the results; and
- Broader multi-disciplinary embeddedness in previous literature and global experience on the innovation impact of emissions trading schemes.

More specifically, Reviewer 3 has firstly requested more clarifications regarding the methodology, including the source of variation in the selected method and how such selection affects the study results. Secondly, the reviewer suggests that more compelling evidence be given for the results from the interaction between ETS status and multiple programme design characteristics. Thirdly, the review calls for a greater discussion regarding the potential underestimation in results resulting from the unregulated firms and improved results interpretation specifically regarding spill over estimates. And lastly, it is requested that the study be better framed in a wider literature context on EU ETS and China low-carbon innovation.

The authors have addressed most of these concerns. Some further refinements in how clearly the results are conveyed may still be possible. For example, Reviewer 3's suggestions such as clarifying the limited innovation impact of the pilot ETS in the title, abstract and elsewhere to avoid the impression that the innovation impact has been significant, or 'grand', are still eluded in the revised version.

The authors have adequately situated their discussion within the wider context of ETS analysis and policy literature of the EU as well as those literatures that discuss the interactive effects of ETS with other instruments.

Reviewer #5 (Remarks to the Author):

This paper investigates the innovation effect from China’s pilot emissions trading with the adoption of patents and firm-level data. A a quasi-experimental design has been adopted which is quite similar to the work done by Calel and Dechezleprêtre (2016). The authors are worth credit as a substantial work has been done to conduct this research, however, there are still a list of limitations that prevent the work to go further.

1. The work is new in the ETS practice in China but lack of novelty among literature. Not only compare to Calel and Dechezleprêtre (2016), but also to a list of previous literature (e.g. Hanley et.al., 2018, working paper. The latest) that investigate the impacts of environmental regulations on firms’ green innovation. Just by adopting the data in a developing country can not be said as “innovative”. A more detailed explanation may need to be added to make the innovation point clear to the readers. Especially, the authors need to state the difference between their work and the work done by Cui et.al., (2018), literature 20.

Douglas Hanley, Chengying Luo, Mingqin Wu. 2018. Environmental Regulation and Enterprises' Green Innovation: Evidence from a Quasi-natural Experiment. https://chengyingluo.weebly.com/uploads/1/1/3/8/113805497/regulation_and_innovation.pdf

2. The second concern is the distinguishment of low-carbon inventory of patents. IPC green inventory is kind of insufficient as the classification is much broader. The author claim that they subjectively select the areas relating to climate mitigation. I do not think such selection is reasonable enough. A deeper review of previous literature on the definition of low-carbon inventory patents would be helpful. And to improve the patent selection process, Veefkind et.al., (2012), and Hongxiu Li (2016).

V. Veefkind, J. Hurtado-Albir, S. Angelucci, K. Karachalios, N. Thumm. 2012. A new EPO classification scheme for climate change mitigation technologies. World Patent Information 34: 106-111.

Hongxiu Li. 2016. Innovation as Adaptation to Natural Disasters. Working paper. https://uwaterloo.ca/economics/sites/ca.economics/files/uploads/files/disaster_innovation.pdf

3. Policy overlapping. The authors match the ETS firm to one or more non-ETS firms so to compare the effect of ETS. Beside the trial of ETS pilots, China also launched the low-carbon pilots (See details on the website of NDRC). So it is possible that a ETS firm is paired with the firm that belongs to low-carbon pilots. The results could be biased as both of the firms are affected by emissions control measures. So the matching can be revised with the consideration of policy overlapping.

In general, this is a good work, but it is hard to see any inspired points.

Response to Reviewer #4

Reviewer #4 (Remarks to the Author):

Reviewer 3 (Substitute)
Note: This second review focuses on how the authors acknowledge and incorporate the first review by the original reviewer (Reviewer 3). Therefore, this is not a full review and instead is meant to be a focused evaluation of the following specific points raised by Reviewer 3. The original reviewer's main concerns relate to
• Clearer communication of the results; and
• Broader multi-disciplinary embeddedness in previous literature and global experience on the innovation impact of emissions trading schemes.

More specifically, Reviewer 3 has firstly requested more clarifications regarding the methodology, including the source of variation in the selected method and how such selection affects the study results. Secondly, the reviewer suggests that more compelling evidence be given for the results from the interaction between ETS status and multiple programme design characteristics. Thirdly, the review calls for a greater discussion regarding the potential underestimation in results resulting from the unregulated firms and improved results interpretation specifically regarding spill over estimates. And lastly, it is requested that the study be better framed in a wider literature context on EU ETS and China low-carbon innovation.

Thanks for the nice summary of the previous reviewer's comments and the evaluation of the authors' revisions and responses.

The authors have addressed most of these concerns. Some further refinements in how clearly the results are conveyed may still be possible. For example, Reviewer 3's suggestions such as clarifying the limited innovation impact of the pilot ETS in the title, abstract and elsewhere to avoid the impression that the innovation impact has been significant, or 'grand', are still eluded in the revised version.
The authors have adequately situated their discussion within the wider context of ETS analysis and policy literature of the EU as well as those literatures that discuss the interactive effects of ETS with other instruments.

We appreciate the opportunities to further discuss how to interpret the size of the estimated policy effect. In what follows we review the discussions made in the previous revisions and highlight the changes made in this round of revisions.

Reviewer #4 suggests that one of reviewer #3's comments – limited innovation effect being not clearly conveyed – was not fully addressed. In the previous response letter, the authors responded to the comment by explaining that there were different ways of interpretation: the effect was extremely large at the individual firm level, smaller but still large and significant for all ETS firms (which include outliers with a lot more

patenting than average firms), and small relative to the overall regional patenting (excluding contribution from spillover effects, which cannot be accurately estimated). The authors then concluded that the interpretation of the size of the policy effect is context-specific, depending on whether the effect is for an average firm (not including outliers that were not matched in the sample for estimation), all ETS firms (including outliers), or for the whole region (including non-ETS firms but not considering policy spillovers). So a simple statement of limited innovation impact may not be preferred. Instead, detailed interpretation was provided in the main article (line 123-138, page 4-5).

The authors agree that some statistics could be presented in the abstract instead of the qualitative wording, as other articles usually do. Previous revision did not include such changes because with limited space of the abstract, 1) the rich context of interpretation as explained above could not be presented, and 2) the spillover effect, which also contributed to the overall impact, could not be accurately estimated and explained. Considering the limited space, the revised version added the increase from ETS firms while leaving spillover effects not included in the overall impact. Now the related statement in the abstract reads: "… China's pilots increased low-carbon innovation of ETS firms by 5-10% without crowding out their other technology innovation. The increase from ETS firms led to about 1% increase of the regional low-carbon patents, while a similar increase from large non-ETS firms was also induced by the ETS."

Reviewers #4 and #3 also suggest some changes to the title to reflect limited innovation effect. As explained above, simply claiming that the innovation effect was limited without providing relevant contexts may also mislead readers. But the authors acknowledge the fact that the title, as a statement, may suggest a grand effect. Therefore, the title is changed as phrase without any conclusive statement. Now the title reads "Low-carbon innovation induced by emissions trading in China." It only indicates the topic of the article but not the conclusion of substantial (or limited) innovation.

Response to Reviewer #5

Reviewer #5 (Remarks to the Author):

This paper investigates the innovation effect from China's pilot emissions trading with the adoption of patents and firm-level data. A a quasi-experimental design has been adopted which is quite similar to the work done by Calel and Dechezleprêtre (2016). The authors are worth credit as a substantial work has been done to conduct this research, however, there are still a list of limitations that prevent the work to go further.

Thanks for the summary.

1. The work is new in the ETS practice in China but lack of novelty among literature. Not only compare to Calel and Dechezleprêtre (2016), but also to a list of previous literature (e.g. Hanley et.al., 2018, working paper. The latest) that investigate the impacts of environmental regulations on firms' green innovation. Just by adopting the data in a developing country can not be said as "innovative". A more detailed explanation may need to be added to make the innovation point clear to the readers. Especially, the authors need to state the difference between their work and the work done by Cui et.al., (2018), literature 20.

Douglas Hanley, Chengying Luo, Mingqin Wu. 2018. Environmental Regulation and Enterprises' Green Innovation: Evidence from a Quasi-natural Experiment. https://chengyingluo.weebly.com/uploads/1/1/3/8/113805497/regulation_and_innovation.pdf

We appreciate the opportunity to discuss the novelty of our research again. We will briefly summarize similar explanations in the previous response letter to avoid redundancy and add some new thoughts. Similar explanations in details can be seen on page 2-5 of the previous response letter.

Reviewer #5 mentions three articles on a similar topic or in a similar context. Our discussions regarding the articles are intended to show the differences in research design and questions to be addressed, rather than to serve as critiques of the articles.

Calel and Dechezleprêtre (2016) provide a solution to policy evaluation using count data (e.g. patents commonly used in innovation research) and show that emissions trading can increase low-carbon innovation without crowding out other technology innovation. It is on this basis the authors raise two scientific questions: 1) whether an ETS (as a market-based instrument) has an effect in the institutional context without much experience of market instruments and with other low-carbon policies that may cause distortions and interact with an ETS; 2) whether policy spillovers and design features matter to the induced-innovation effect. Both questions call for findings that advance our understanding of the mechanism, scope of influences, and specific design

in the application of a policy instrument. They do not solicit a specific developmental stage of a research context.

The two research questions differ this research from Hanley et al. (2018) and Cui et al. (2018). Hanley et al. (2018) focus on one version of the Porter Hypothesis – whether stringent environmental regulations promote innovations – and test it in a developing country context, i.e. China. Cui et al. (2018) focus on whether a policy announcement promotes innovation (explained below). While this research investigates an ETS and the Chinese context, it focuses on the effect of the policy instrument out of specific ETS program designs and interactions with other policy distortions. The policy effect from policy interaction and specific program design have attracted theoretical discussions but lack empirical evidence. The empirical evidence provided by this research help to advance our understanding and future application of the ETS as a market-based instrument.

This research also differs from Cui et al. (2018) in research design. While they are not explicitly about the question asked, the research design of Cui et al. (2018) suggests that they focus on the effect of the announcement of ETS in 2011 rather than of the actual ETS program. In their difference-in-difference-in-differences, they use a small dataset of publicly-listed firms, who are business elites with a lot more patents than average ETS and non-ETS firms (the majority of ETS firms are not publicly-listed and therefore not included in their sample); they compare before and after the year of announcing policy experimentation of ETS in 2011, not the actual launch of the individual pilots in 2013-2014; they use the *union* of individual programs' sectoral coverage at two-digit industry level as the sectoral treatment dummy for the whole country, but the sectoral coverage varies across programs and the actual treatment assignment is on specific firms in certain four-digit sectors, not on two-digit sectoral level (only a portion of firms in an ETS sector are actually included in the ETS); they use ETS pilot regions as the regional treatment dummy but exclude the Shenzhen ETS, because the Shenzhen ETS has firms from all the two-digit sectors, and would make the sectoral treatment dummy equal 1 for all the firm observations when being included. A result of this research design is that the majority of firms assigned to the treatment group are not actually in any ETS. This briefly explains why the research design may be able to test an effect of policy announcement, but not the actual effect of an ETS, let alone our focus of policy interaction and program design. To avoid redundancy, this is a brief summary of the previous explanations, which can be found on page 3-4 of the previous response letter. Our research design avoided all these issues to directly test the ETS effect.

In response to the comment, the authors tried to make the scientific questions of this research more explicit by revising the last sentence of one of the introductory paragraphs (page 2, line 51-53). Now it reads "But not much is known about the scope of ETS effects among regulated and unregulated firms and influences from market design, especially when there are other policy influences."

2. The second concern is the distinguishment of low-carbon inventory of patents. IPC green inventory is kind of insufficient as the classification is much broader. The author claim that they subjectively select the areas relating to climate mitigation. I do not think such selection is reasonable enough. A deeper review of previous literature on the definition of low-carbon inventory patents would be helpful. And to improve the patent selection process, Veefkind et.al., (2012), and Hongxiu Li (2016).

V. Veefkind, J. Hurtado-Albir, S. Angelucci, K. Karachalios, N. Thumm. 2012. A new EPO classification scheme for climate change mitigation technologies. World Patent Information 34: 106-111.

Hongxiu Li. 2016. Innovation as Adaptation to Natural Disasters. Working paper. https://uwaterloo.ca/economics/sites/ca.economics/files/uploads/files/disaster_innovation.pdf

This comment concerns the measurement of low-carbon innovation using some categorization of patent data. In general, there are two strategies: one uses the classes and subclasses from existing classification systems, and the other applies some search criteria and keywords to the textual information of patents. The first is the common choice in environmental research (e.g. Calel and Dechezleprêtre 2016; Cui et al. 2018), thanks to the available green inventories and classification schemes. The second is the only choice when the first is not available, as in the case of Li (2016) mentioned by the reviewer above.

When using the first strategy, there are also alternative choices of green inventories and associated classification systems. Two major efforts were made for the inventories and classification to be available. The first was the International Patent Classification (IPC) Green Inventory launched by the World Intellectual Property Organization (WIPO) in 2010 based on the technologies identified by the United Nations Framework Convention on Climate Change and the existing IPC code. The second was a new Y02 patent classification developed by the European Patent Office (EPO) in 2010 jointly with the United Nations Environmental Program and the International Centre on Trade and Sustainable Development. Both efforts were done by the leading institutions and experts, of high quality, and widely acknowledged. Veefkind et al. (2012), as the reviewer mentions above, suggest that the Y02 is superior because directly as a classification scheme, it is more convenient for users to find green technologies than an inventory, by which a user has to go back to the IPC code. There is no comparison of the accuracy of the two efforts in categorizing low-carbon technologies.

In empirical sense, the IPC is developed by the WIPO and widely used by more than 100 patent offices worldwide, while the Cooperative Patent Classification System, which includes the Y02, is used by the EPO and the United States Patent and Trademark Office. This explains why research in the Chinese context mostly uses the IPC and the IPC Green Inventory when possible (e.g. Cui et al. 2018), while research in the EU

context may use the CPC and Y02 (e.g. Calel and Dechezleprêtre 2016).

While the background information above explains why the IPC Green is a reasonable choice in this research context, the authors would like to highlight two challenges to use the alternative classification – availability and reliability – and the efforts made to make the findings more reliable.

The first issue is that the IPC code is the only available classification information for patents filed at China's State Intellectual Property Office (SIPO). The SIPO hires experts to review every patent filed and assign IPC codes to it. This is the original and most reliable source of SIPO patents, and is publicly accessible. Therefore, results based on the selected classes in the IPC Green Inventory, as done in this research, can be easily reproduced by any researcher using publicly available data.

As a commercial database provider, the Clarivate Analytics's Derwent Innovation (Originally the Thomson Innovation) hosts SIPO patents with CPC codes. But it is unclear the process by which the database provider collects patent entries from the SIPO and assigns CPC codes to each entry. It is therefore unclear to the authors the reliability of the classification. In addition, most researchers, including the authors, have no access to the Derwent Innovation patent database.

Having said all these, some efforts have been done. Firstly, an alternative, narrower scope of low-carbon technology, including only low-carbon power generation and energy conservation in manufacturing in the IPC Green, was used for robustness check. The point estimate and upper bound estimate were the same as that of the main estimation, while the lower bound estimate increased from 0.5 to 1.

Secondly, in response to the comment, the authors managed to get a Derwent Innovation's version of the SIPO data from a source with data access, only for robustness checks. The data was merged with other datasets and estimated using the main estimation strategy, with the dependent variable created according to the Y02 classification. The estimated ETS effect was 1 (1, 1.9) low-carbon patent, i.e. a smaller point estimate and larger lower bound than the main estimation. The estimated effect on non-low-carbon technologies was 0.75 (-0.9, 1.9), i.e. a smaller lower bound than the main estimation. The general findings are not affected.

The results based on Y02 classification are not reported in the main article or the supplementary information, because the authors do not have access to the Derwent Innovation data in principal. Instead, revision in the main article was made following the reviewer's comment. Additional explanations have been added to the Methods section (page 11, line 295-297) "An alternative categorization of low-carbon technologies, Y02 classification, was not feasible, because the SIPO patent entries do not contain related classification codes."

3. Policy overlapping. The authors match the ETS firm to one or more non-ETS firms so to compare the effect of ETS. Beside the trial of ETS pilots, China also launched the low-carbon pilots (See details on the website of NDRC). So it is possible that a ETS firm is paired with the firm that belongs to low-carbon pilots. The results could be biased as both of the firms are affected by emissions control measures. So the matching can be revised with the consideration of policy overlapping.

We appreciate the opportunity to highlight different matching strategies that have been used and the motivation behind each strategy. The main strategy intentionally matches ETS firms with non-ETS firms in low-carbon pilot regions (using an exact match condition for low-carbon pilot region), which include both ETS pilot regions and non-ETS low-carbon pilot regions. The strategy addresses one of the main research questions: whether the ETS has any effect on top of the existing climate policies and associated distortions or interactions. Matching the ETS firms with firms outside the low-carbon pilot regions would not be able to answer this question.

Alternatively, in Table S10 of the supplementary information, the ETS firms are matched with non-ETS firms in ETS pilot regions, to rule out any other location-specific influences that may affect firm innovation. Table S11 uses different baseline periods in matching to rule out unobservable selection bias from firm or government discretion. Table S8 and Table S16 test other matching specifications and method.

In the previous response letter, the ETS firms are matched with non-ETS firms strictly in a different location, to separate two margins of the identification strategy (page 9). The ETS firms are matched with firms in the non-ETS low-carbon pilot region, to avoid influences from policy spillovers (page 26). The ETS firms are matched with firms likely subject to spillovers to compare the direct policy effect from the spillover effect (page 29).

In general, this is a good work, but it is hard to see any inspired points.

It is important not only to confirm whether a policy scheme is effective in inducing directed innovation, but also to understand how it works in different policy contexts and policy designs. We believe that our efforts to directly confront the policy interaction and investigate alternative ETS program designs (especially mass-based and rate-based allocation) are significant in advancing the scholarly understanding of emissions trading, and are informative to future market instrument designs.

We hope that the responses above address your concerns and clarify our contribution, and are happy to respond to further comments.

REVIEWERS' COMMENTS:

Reviewer #4 (Remarks to the Author):

Thank you for addressing the points raised in the second round of reviews.

Reviewer #5 (Remarks to the Author):

The authors present a lot of the spaces in responding the comments but avoid to answer the comments diectly. And little changes have been made to the manuscript. I still hold my opinion that this is a good work, but it is hard to see any inspired points. So I would suggest another reviewer to review the paper.

Reviewer #6 (Remarks to the Author):

The points evidenced by the reviewers have been properly discussed by the author(s). Thus, the paper can be accepted for publication.

Response Letter for

“Low-carbon innovation induced by emissions trading in China”

Reviewer #4 (Remarks to the Author):

Thank you for addressing the points raised in the second round of reviews.

Thank you for your review.

Reviewer #5 (Remarks to the Author):

The authors present a lot of the spaces in responding the comments but avoid to answer the comments diectly. And little changes have been made to the manuscript. I still hold my opinion that this is a good work, but it is hard to see any inspired points. So I would suggest another reviewer to review the paper.

Thank you for your review. We made extensive explanations in response to your previous comments on novelty, measurement, and policy interaction, and quoted in the previous response letter the revisions in the manuscripts.

Reviewer #6 (Remarks to the Author):

The points evidenced by the reviewers have been properly discussed by the author(s). Thus, the paper can be accepted for publication.

Thank you for your review.
